# Immunomagnetic‐MXene Bifunctional Probes for Ultrasensitive and Specific Terahertz Metasensing of Tumor‐Derived Exosomes

**DOI:** 10.1002/advs.78093

**Published:** 2026-09-30

**Authors:** Weidong Jin, Jiyue Chen, Fengxin Xie, Huiyan Tian, Xuechen Dou, Yanqi Han, Lu Zhang, Jining Li, Xiang Yang

**Affiliations:** ^1^ Department of Laboratory Medicine Southwest Hospital Army Medical University (Third Military Medical University) Chongqing China; ^2^ Institute of Laser and Optoelectronics School of Precision Instrument and Optoelectronics Engineering Tianjin University Tianjin China

**Keywords:** bifunctional probes, MXene, pancreatic cancer, terahertz metamaterials, tumor‐derived exosomes

## Abstract

Tumor‐derived exosomes (tExos) are promising biomarkers for early pancreatic cancer (PC) diagnosis, yet current detection methods are limited by cumbersome implementation, high sample consumption, and complex matrix interference. Herein, we developed immunomagnetic‐MXene bifunctional probes coupled with terahertz (THz) metamaterials to synergistically increase the sensitivity and specificity of PC‐derived tExos detection. Antibodies against ZIP4 and glypican‐1 (GPC‐1) were separately conjugated to magnetic beads (MBs) and MXene nanosheets, which yielded two probes that simultaneously targeted PC‐derived tExos, enabling highly specific sandwich‐type capture of target exosomes. Moreover, MXene increased the tExo‐induced THz metamaterial resonance and outperformed AuNPs in signal amplification, endowing the method with a limit of detection (LOD) of 647 particles mL^−1^. The developed THz metasensor achieved accurate detection of early tumor formation and evaluation of therapeutic responses in PC mouse models. In a 60‐subject clinical cohort, it distinguished all‐stage PC patients from healthy controls with an area under the curve (AUC) of 0.997. Compared with that of the conventional CA19‐9 assay, our THz metasensor demonstrated superior diagnostic accuracy in 11 early‐stage PC patients (AUC: 0.991 vs. 0.809), with a simplified workflow, minimal sample requirement (10 µL), and low cost (< $5), highlighting its potential for clinical translation in early PC diagnosis.

## Graphical abstract

An immunomagnetic‐MXene bifunctional probe integrated with THz metamaterials enables ultrasensitive and specific detection of pancreatic cancer‐derived exosomes. Dual biomarker recognition ensures high specificity, while MXene signal amplification achieves a detection limit of 647 particles mL^−1^. Clinical validation demonstrates superior diagnostic accuracy over conventional CA19‐9 assays, offering a promising tool for early pancreatic cancer screening.

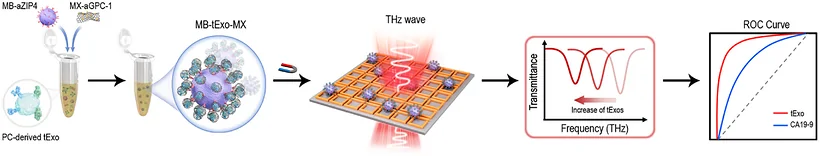

## 1 Introduction

Pancreatic cancer (PC) is a highly malignant neoplasm with a poor prognosis. Most PC patients are diagnosed at advanced or metastatic stages, which underscores the urgent need for early diagnosis to improve survival outcomes [1, 2]. To date, histopathological biopsy remains the gold standard for PC diagnosis [3]. However, owing to the deep anatomical location of the pancreas, the biopsy procedure is associated with a risk of unavoidable procedural trauma to patients, limiting its utility for routine screening. As the most widely used serum biomarker in the clinical management of PC, carbohydrate antigen 19‐9 (CA19‐9) has poor specificity, as elevated CA19‐9 expression is frequently observed in benign pancreatic diseases such as pancreatitis [4]. Conventional imaging modalities, including ultrasound, computed tomography (CT), and magnetic resonance imaging (MRI), lack sufficient sensitivity for detecting small early‐stage tumors [5, 6]. Collectively, these limitations highlight the need to develop accurate and noninvasive methods for early PC diagnosis.

Exosomes are lipid bilayer vesicles with sizes ranging from 40 to 160 nm that have emerged as promising biomarkers for PC screening [7, 8, 9]. Accumulating evidence has demonstrated that tumor‐derived exosomes (tExos) play critical roles in PC initiation, progression, and metastasis and that their circulating levels are closely correlated with tumor stage and malignant potential [10, 11, 12]. Therefore, the quantitative detection of tExo levels in body fluids represents a promising minimally invasive strategy for early PC diagnosis [13, 14, 15]. Conventional approaches for exosome detection mainly include enzyme‐linked immunosorbent assays (ELISAs), nanoflow cytometry, nanoparticle tracking analysis (NTA), and dynamic light scattering (DLS). However, their widespread clinical application is hampered by cumbersome operational procedures, large sample volume requirements, and insufficient detection specificity [16, 17, 18]. Thus, developing highly sensitive and specific technologies for tExo detection is critical for early PC diagnosis.

Recently, terahertz (THz) spectroscopy has become a powerful tool for exosome detection. This potential derives from three intrinsic features of the THz band: (1) exosomal biomolecules (DNA, RNA, proteins, and lipids) exhibit rotational and collective vibrational modes within 0.1–10 THz [19, 20, 21]; (2) the dielectric contrast between the exosomal lipid bilayer and aqueous core provides a basis for spectral discrimination [22]; and (3) non‐ionizing THz photons [23] enable non‐destructive, repeated measurement of intact exosomes. However, these merits do not ensure high detection sensitivity in free‐space configurations, as the wavelength mismatch between THz radiation and nanoscale exosomes inherently weakens wave–particle interactions, yielding spectral perturbations that are too subtle to resolve reliably [24, 25]. To overcome this fundamental limitation, THz metamaterials (MMs) have been utilized as effective solutions to increase the interactions between THz waves and biological samples. Nevertheless, conventional MMs suffer from inherent ohmic and radiative losses, resulting in low *Q* factors that limit the sensitivity for low‐abundance biomarkers [26]. For example, Wang et al. demonstrated exosome recognition using antibody‐functionalized MMs for colorectal cancer detection, but such conventional designs lack the detection capability required for early‐stage diagnosis [27]. Recently, quasi‐bound states in the continuum (QBICs) with ultrahigh *Q* factors have revolutionized THz MM design, enabling significantly increased sensitivity for exosome detection. In a recent study, a QBIC‐based THz MM achieved robust dynamic responses across a wide range of gastric cancer‐derived exosome concentrations, ranging from 1 × 10^4^ to 1 × 10^8^ particles mL^−1^, with a limit of detection (LOD) as low as 1 × 10^4^ particles mL^−1^ [28]. Nonetheless, this method may still be insufficient for accurately capturing the subtle disturbances induced by trace amounts of target exosomes in the early stage of disease. Moreover, this strategy of separately modifying antibodies on both sides of the sensor increases operational complexity and can result in antibody cross‐contamination, which leads to nonspecific binding and compromises target recognition specificity [29]. Overcoming these interrelated obstacles requires a facile integrated strategy that synergistically increases both sensitivity and specificity.

As emerging two‐dimensional (2D) nanomaterials, MXenes possess superior metallic conductivity, a large specific surface area, and abundant surface functional groups [30]; they have been extensively applied in optical sensing fields such as infrared, surface enhanced Raman scattering (SERS) [31], fluorescence [32], and surface plasmon resonance (SPR) [33, 34] for the ultrasensitive detection of various biomolecules. Our previous study demonstrated that MXene‐functionalized THz MMs achieved ultrasensitive detection of thrombosis components via the interfacial charge transfer effect between the MXene and target molecules [35], demonstrating that MXenes are effective THz metasurface modifiers with excellent signal amplification capabilities. A previous study demonstrated that MXenes trigger SPR in the THz region and generate local field enhancement through coupling with metasurfaces [36]. Moreover, MXenes possess a high refractive index in the low‐frequency THz range, which greatly disturbs the local dielectric environment and further induces frequency shifts and profile changes in THz resonant peaks [37]. Additionally, the surface terminal groups of MXenes produce polarization relaxation under alternating THz electric fields, endowing the material with strong THz absorption and scattering loss properties [38]. Therefore, MXene labeling could serve as a robust strategy to amplify the THz sensing response of tExos. However, previous MXene‐based THz studies have primarily exploited MXenes as metasurface modifiers [35], absorbers [39], or shielding materials [40]. In contrast, the use of MXenes as signal amplification tags in THz biosensing has, to the best of our knowledge, not previously been reported.

Another challenge lies in the highly specific isolation of tExos from complex serum matrices. Immunomagnetic separation has become a mainstream technique for the specific isolation of exosomes because of its advantages of simple operation, high efficiency, and favorable specificity [41, 42]. The zinc transporter ZIP4 is highly expressed in PC‐derived tExos [43, 44, 45, 46] and is therefore an excellent target for immunocapture. Nevertheless, the inherent heterogeneity of exosomes limits the specific recognition of tExos by a single biomarker. Glypican‐1 (GPC‐1), another promising biomarker for PC diagnosis, is also highly enriched in PC‐derived tExos [47, 48]. Accordingly, a dual‐biomarker‐based bifunctional probe strategy could provide significant advantages. Through the synergistic recognition of both biomarkers, such probes could enable more efficient and specific enrichment of PC‐derived tExos, while the aforementioned optoelectronic properties of MXene probes could simultaneously increase the THz sensing response.

In this study, we developed an integrated platform that combined immunomagnetic‐MXene bifunctional probes with QBIC‐based THz MMs for the ultrasensitive and specific detection of PC‐derived tExos to enable accurate early PC diagnosis. To achieve high‐specificity tExo isolation, anti‐ZIP4 and anti‐GPC‐1 antibodies were conjugated separately to magnetic beads (MBs) and Ti_3_C_2_ MXene nanosheets, which yielded specific MB‐aZIP4 and MX‐aGPC‐1 probes, respectively. These two probes simultaneously recognized and captured tExos, forming sandwich‐structured bifunctional MB‐tExo‐MXene (MB‐tExo‐MX) probes that were readily isolated via magnetic separation. To maximize the detection sensitivity of tExos, rationally designed asymmetric QBIC‐based THz MMs with high interfacial dielectric responsiveness were constructed. The MXene probes anchored to MB‐tExo‐MX led to a dramatic increase in the tExo‐induced THz signals. Combined with the high performance of the QBIC MM, this method enabled the ultrasensitive quantitative detection of tExos over a broad linear range (10^3^–10^9^ particles mL^−1^) with an ultralow LOD of 647 particles mL^−1^. By monitoring dynamic changes in circulating tExo levels, the developed platform enabled the sensitive detection of early tumor formation and assessment of the therapeutic response in PC xenograft mouse models. In human clinical samples, the THz metasensor accurately distinguished PC patients at all stages from healthy controls; notably, it exhibited promising diagnostic performance in 11 early PC patients, with an area under the curve (AUC) of 0.991, and outperformed the conventional CA19‐9 assay (AUC = 0.809). This work establishes a powerful platform for early PC diagnosis and progression monitoring and demonstrates the potential of MXene‐enhanced QBIC‐THz sensing for widespread application in liquid biopsy and precision medicine.

## 2 Results and Discussion

### 2.1 Principle of the THz Metasensor for the Quantitative Analysis of tExos

The working principle of the THz metasensor for the quantitative detection of PC‐derived tExos is illustrated in Figure 1a. Anti‐ZIP4 and anti‐GPC‐1 antibodies were conjugated separately on MBs and MXene nanosheets to fabricate the MB‐aZIP4 and MX‐aGPC‐1 probes, respectively. On the basis of the simultaneous recognition and capture of PC‐derived tExos by the dual probes, sandwich‐structured bifunctional MB‐tExo‐MX complexes were formed through a one‐step process within complex serum matrices. The resulting complexes were then magnetically separated and transferred onto the surface of the QBIC MM for THz measurement. Owing to the high dielectric constant and excellent THz absorption capability of MXene [36, 49], MXene probes greatly amplified the tExo‐triggered THz signal, resulting in a redshift of the resonance frequency and an increase in the transmittance at the resonance minimum; in contrast, exosomes from normal cells and those expressing only one target protein did not induce the THz signal associated with MXene (Figure S1). Clinically, the proposed THz metasensor enabled the accurate early diagnosis of PC and the assessment of PC progression by analyzing the THz signals induced by tExos in patient serum (Figure 1b). Compared with conventional biomarker detection methods, this strategy has prominent advantages, such as higher diagnostic accuracy, simple operation, minimal sample consumption and lower detection costs; thus, it has excellent potential for clinical translation for the early screening of PC.

**FIGURE 1:**
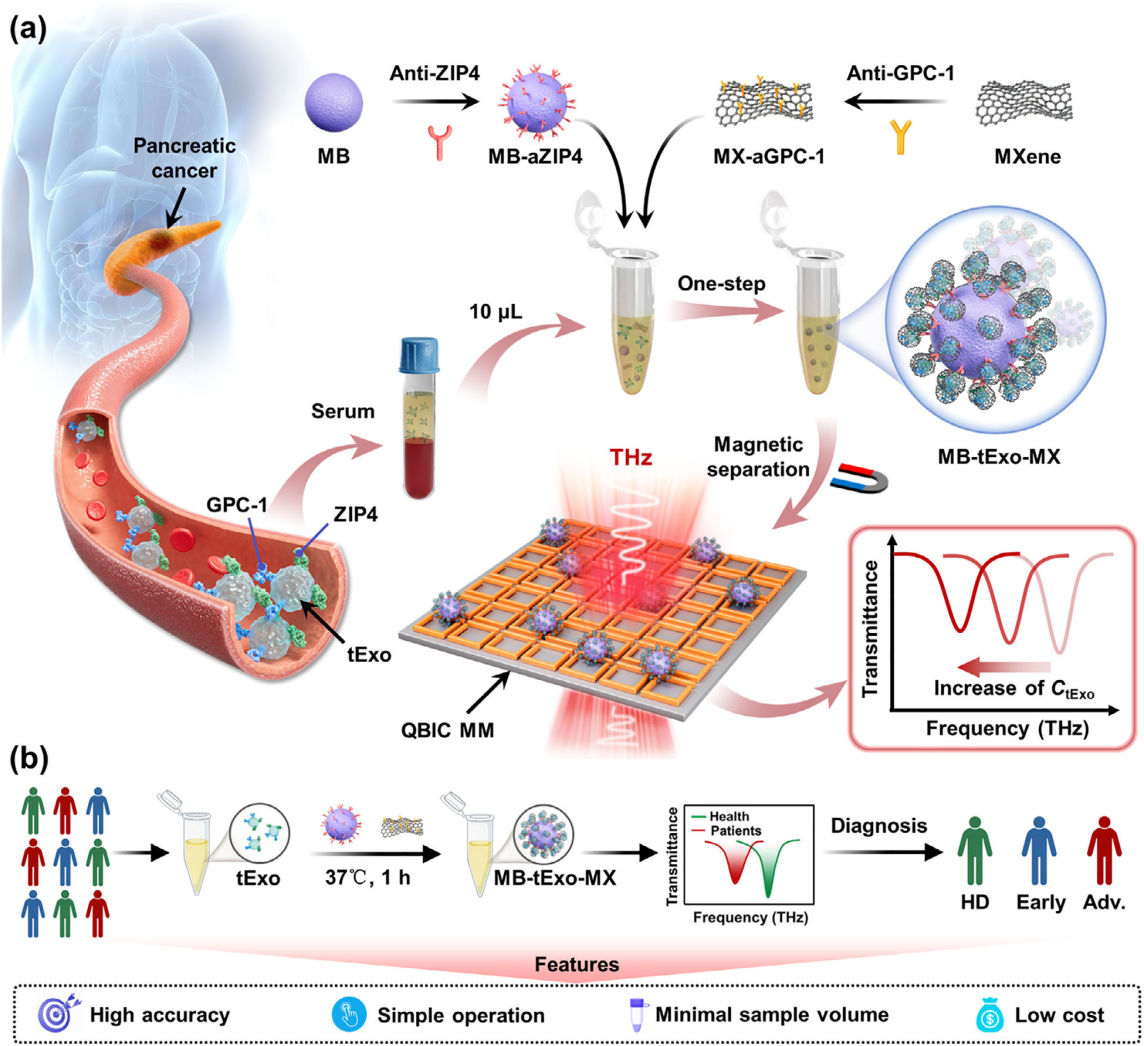
Schematic diagram of immunomagnetic‐MXene bifunctional probes integrated with THz metasensing for accurate pancreatic cancer diagnosis. (a) Schematic illustration of the working principle of the THz metasensor for the quantitative detection of tExos from PC. (b) Workflow and features of the THz metasensor for early pancreatic cancer diagnosis and progression evaluation. MB, magnetic bead; MX, MXene; tExo, tumor‐derived exosome; QBIC MM, QBIC‐based metamaterial; HD, healthy donor; Early, early stage; Adv., advanced stage.

### 2.2 Synthesis and Characterization of Immunomagnetic‐MXene Bifunctional Probes

A schematic of the dual probes strategy in which MB‐aZIP4 and MX‐aGPC‐1 simultaneously recognize ZIP4 and GPC‐1 on target tExos to form sandwich‐structured bifunctional MB‐tExo‐MX complexes is presented in Figure 2a. PANC‐1‐derived tExos were used as model tExos and were isolated by ultracentrifugation [50]. Transmission electron microscopy (TEM) revealed that the tExos exhibited a typical cup‐shaped membrane morphology (Figure 2b), confirming their successful isolation [13]. NTA revealed that the particle size of the tExos was 145.3 ± 3.6 nm (Figure 2c). To evaluate the specificity of PC‐associated biomarkers for tExo detection, we isolated exosomes from PANC‐1, MDA‐MB‐231 and HPNE cells and compared their protein profiles by Western blot. Exosomal identity was verified by the positive expression of canonical markers (CD9, CD63, CD81 and TSG101) in isolated exosomes and the absence of calnexin in exosomal preparations from all three cell lines (Figure 2d and Figure S2). Among the PC‐associated candidates, both GPC‐1 and ZIP4 were highly expressed in PANC‐1‐derived tExos. However, GPC‐1 was also markedly expressed in MDA‐MB‐231‐derived tExos, indicating that GPC‐1 alone lacks sufficient specificity for distinguishing PC‐derived tExos from other cancer‐derived exosomes. In contrast, ZIP4 was specifically enriched in PANC‐1‐derived tExos but absent from both MDA‐MB‐231 and HPNE exosomes, demonstrating that the combination of GPC‐1 and ZIP4 can substantially improve detection specificity. To further verify the co‐expression of both biomarkers at the single‐particle level, nano‐flow cytometry was performed. As shown in Figure 2e, the GPC‐1^+^/ZIP4^+^ double‐positive tExos accounted for 41.9% of the total tExo population, confirming that a substantial fraction of PANC‐1‐derived tExos simultaneously express both biomarkers.

**FIGURE 2:**
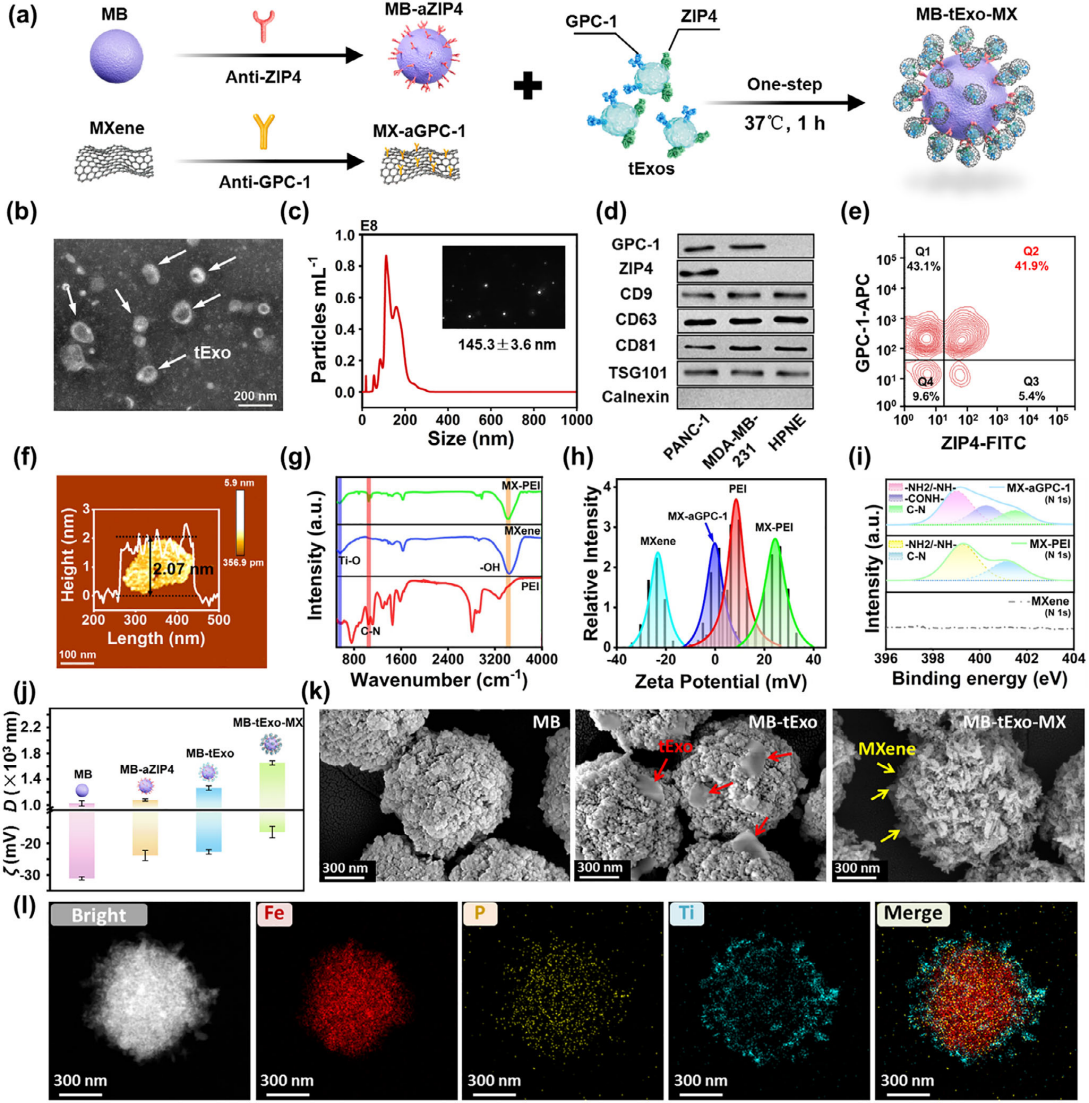
Synthesis and characterization of immunomagnetic‐MXene bifunctional probes. (a) Schematic diagram of the construction of bifunctional probes and one‐step sandwich formation of MB‐tExo‐MX. (b) TEM of PANC‐1‐derived tExos. Scale bar: 200 nm. (c) NTA results for the size distribution of tExos; the particle size is presented as the mean ± SD of three independent measurements. (d) Western blot analysis of exosomal markers (CD9, CD63, CD81, and TSG101), the endoplasmic reticulum‐resident protein calnexin, and PC‐associated biomarkers (GPC‐1 and ZIP4) in exosomes isolated from PANC‐1, MDA‐MB‐231, and HPNE cells. (e) Nano‐flow cytometry analysis of PANC‐1‐derived tExos. The Q2 population represents GPC‐1^+^/ZIP4^+^ double‐positive tExos. (f) AFM image of an MXene nanosheet. (g) FT‐IR spectra of MXene‐PEI (green), MXene (blue), and PEI (red). (h) Zeta potential analysis of MXene, PEI, MX‐PEI, and MX‐aGPC‐1. (i) High‐resolution XPS spectra of the N 1s peaks of MXene, MX‐PEI and MX‐aGPC‐1. (j) Zeta potentials and diameters of MB, MB‐aZIP4, MB‐tExo and MB‐tExo‐MX. *ζ*, zeta potential; *D*, diameter. Data are presented as the mean ± SD of *n* = 3 replicates. (k) SEM images of MB, MB‐tExo and MB‐tExo‐MX. Scale bar: 300 nm. (l) Elemental mapping analysis of MB‐tExo‐MX. Scale bar: 300 nm.

The characterization of the fabricated MXene probes was performed via Fourier transform infrared spectroscopy (FT‐IR), zeta potential measurements, and X‐ray photoelectron spectroscopy (XPS). As shown in Figure S3a, the Ti_3_C_2_ MXene solution displayed a uniform dark green color with good dispersibility. TEM and DLS confirmed that the MXene nanosheets existed in monolayer dispersion states (Figure S3b), with an average size of 268.20 nm (Figure S3c). Atomic force microscopy (AFM) revealed a thickness of 2.07 nm for the MXene nanosheets (Figure 2f), which is consistent with previous reports [51, 52]. This thickness indicates that the nanosheets were predominantly single‐layered. Prior to antibody modification, polyethyleneimine (PEI) was bound to the surface of the MXene nanosheets to provide abundant binding sites for antibodies. The FT‐IR results verified the successful conjugation of PEI with MXene, as characteristic peaks corresponding to C─N bonds (∼1075 cm^−1^) of PEI, as well as −OH (∼3440 cm^−1^) and Ti─O bonds (∼559 cm^−1^) of MXene, were simultaneously observed in the MX‐PEI spectrum (Figure 2g). The surface engineering of MXene nanosheets also mitigates their oxidative degradation in aqueous environments. The pristine MXene faded markedly after 20 days (Figure S4), whereas the MX‐PEI retained its original color, confirming the antioxidation protection provided by the PEI coating. Zeta potential measurements further confirmed the successful immobilization of anti‐GPC‐1 antibodies on the MXene surface, as a distinct shift in the zeta potential of MX‐PEI was observed following antibody conjugation (Figure 2h). Wide‐scan XPS survey spectra revealed that compared with pristine MXene, MX‐PEI exhibited a distinct N 1s peak at 400.46 eV, confirming the successful immobilization of PEI on the MXene surface (Figure S5a,b). The markedly intensified N 1s signal of MX‐aGPC‐1 verified successful antibody modification (Figure S5c). High‐resolution N 1s XPS data revealed that compared with MX‐PEI, MX‐aGPC‐1 exhibited a characteristic amide bond peak at 400.25 eV (Figure 2i), confirming the functionalization of the antibody on the MXene surface [53]. Following bovine serum albumin (BSA) depletion from the anti‐GPC‐1 antibody solution, the anti‐GPC‐1 antibody binding ratio on MXene was determined by bicinchoninic acid (BCA) assay with a total protein standard curve (Figure S6a), yielding a measured value of 83.33% (Figure S6b). This efficiency compares favorably with the typical immobilization yields reported for antibody conjugation on solid supports [54], which may be attributed to the large specific surface area of the ultrathin MXene nanosheets and the abundant primary amine groups provided by the branched PEI coating.

Finally, the characterization of the formed sandwich‐structured MB‐tExo‐MX complexes was performed via DLS, zeta potential analysis and microscopy. As shown in Figure 2j, both the particle size and the zeta potential of the MBs increased after anti‐ZIP4 antibody modification, indicating the successful construction of the immunomagnetic probe. The binding ratio of the anti‐ZIP4 antibody on the MB surface was 91.32% (Figure S6c), and functional validation of this high antibody loading via NTA quantification revealed that the capture efficiency of the MB‐aZIP4 probes was 75.03%, as calculated from the total particle numbers of the input and the unbound supernatant (Figure S7a–c), demonstrating effective tExo enrichment by the MB‐aZIP4 probes. The subsequent loading of tExos and MXene probes onto the MB surface further increased both the particle size and the zeta potential (Figure 2j, right), which is consistent with the stepwise assembly of the sandwich structure. High‐resolution SEM images clearly revealed the capture of tExos by immunomagnetic probes, to which the MXene probes were bound (Figure 2k). Moreover, elemental mapping analysis of MB‐tExo‐MX verified the characteristic distributions of Fe, P, and Ti (Figure 2l), indicating the successful integration of MBs, tExos, and MXene probes and confirming the formation of the MB‐tExo‐MX complex.

### 2.3 Simulation and Fabrication of QBIC‐Based THz MMs

To achieve ultrasensitive detection of MB‐tExo‐MX, QBIC‐based THz MMs were designed and fabricated via standard micro‐nano processing techniques, with a maximum geometric deviation of 0.2 µm that falls well within the acceptable tolerance. An optical photograph and local microscopy image of a fabricated QBIC MM, in which the resonant units are arranged periodically, are shown in Figure 3a. A schematic diagram and structural parameters of the unit are shown in Figure 3b. Asymmetry *α* was introduced by adjusting the split position. Schematic diagrams of the six asymmetric structures are shown in Figure S8. To elucidate QBIC formation, the THz transmission spectra of the MMs under different *α* values were simulated. As shown in Figure 3c, the resonance in the transmission spectrum disappeared at *α* = 0, at which point the structure was in the symmetry‐protected BIC state with an infinite *Q* factor, and the energy was bound here without leaking into the free space [55]. As *α* increased from 0 to 1.0, a typical resonance characteristic peak appeared, with a redshift and increased linewidth, implying that the symmetry‐protected BIC state began to transform into a QBIC state. The experimental results were generally in good agreement with the simulation results (Figure 3d). The variations in the QBIC resonance and dipole modes under different *α* values are shown in Figure 3e. Clearly, the asymmetric perturbation disrupted the symmetrically protected BIC resonance (star position), which evolved into a QBIC resonance. Furthermore, the relationship between the resonance *Q* factor and the asymmetry *α* was investigated. The transmission spectrum of a typical Fano lineshape can be fitted using the formula T = $|a_{1}+ja_{2}+\frac{b}{\omega -\;\omega_{0}+j\gamma }|$, where *a*_1_, *a*_2_ and *b* are real constant factors, *ω* is the frequency, and *ω_0_* and *γ* represent the resonance frequency and damping rate, respectively [56]. The *Q* factor was calculated as *Q* = *ω_0_*/(2*γ*). Because the simulations incorporated gold with finite conductivity, the extracted *Q* represents the total *Q* factor, which includes both radiative and nonradiative (ohmic) contributions. In the ideal lossless limit, the radiative *Q* factor diverges as *α* approaches zero, giving rise to the symmetry‐protected BIC state, whereas the total *Q* factor remains limited by material losses. Consistently, the total *Q* factor in our simulations decreased monotonically from ≈ 980 at *α* = 0.1 to ≈ 80 at *α* = 1.0 (Figure S9a), reflecting the increased radiative leakage at larger asymmetry.

**FIGURE 3:**
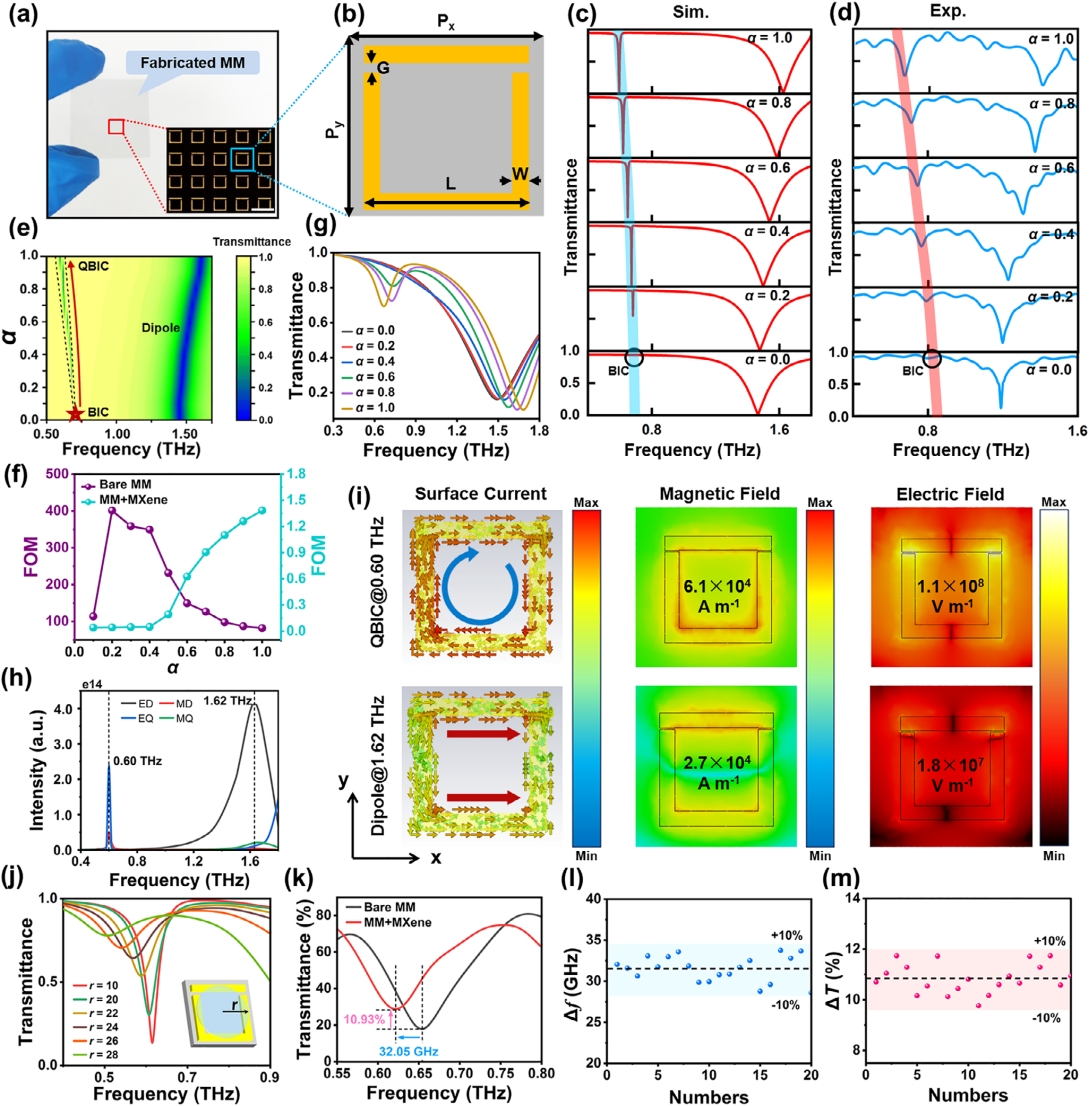
Simulation and fabrication of a QBIC MM. (a) Optical photograph and local magnification of the fabricated THz MM. Scale bar: 100 µm. (b) Structural parameters of one unit in (a). P_x_ = 100 µm, P_y_ = 100 µm, L = 60 µm, W = 8 µm, and G = 1 µm. (c,d) Simulated and measured THz transmission spectra of the QBIC MM with different asymmetries *α*. (e) Simulated mapping of the QBIC MM by varying the parameter *α* value. (f) FOM values of the bare MM and MM loaded with MXene under different degrees of asymmetry *α*. (g) Simulated THz transmission spectra of the QBIC MM with different asymmetry parameter *α* values in the presence of MXene. (h) Multipole expansion results for the QBIC MM. ED, electric dipole; MD, magnetic dipole; EQ, electric quadrupole; MQ, magnetic quadrupole. (i) Surface current, magnetic field and electric field distributions of the THz MM structures for 0.60 and 1.62 THz incident light, respectively. (j) Simulated THz transmission spectra of the QBIC MM with varying radii (*r*) from 10 to 28 µm of MXene coverage. (k) Measured THz transmission spectra of the bare MM and the MXene‐loaded MM. (l, m) Δ*f* and Δ*T* distributions at 20 randomly selected locations on a single chip for the detection of MXene.

Next, the metasurface performance parameters based on the evolution of the QBIC were optimized. The parameter I = |T1 − T2| was introduced to characterize the resonance intensity of the QBIC resonance, where T1 and T2 corresponded to the transmission dip and the transmission peak of the Fano resonance, respectively. Owing to the mutual constraint between the resonance intensity and linewidth, the evolution trend of I was opposite that of the *Q* factor (Figure S9b). To achieve an optimal trade‐off between the *Q* factor and I, a figure of merit (FOM, defined as FOM = *Q* × I) was introduced [57]. As depicted in Figure 3f, the bare MM achieved its maximum FOM with *α* = 0.2, which corresponded to the optimal sensing performance under this structural parameter. However, the QBIC mode at *α* = 0.2 exhibited high sensitivity to electromagnetic absorption from MXene, readily leading to resonant quenching (Figure 3g). Therefore, it was necessary to further determine the optimal structural parameters of the MMs in the presence of MXene. Both the *Q* factor and the resonance intensity I increased as the asymmetry parameter *α* increased (Figure S9c). Moreover, the FOM gradually reached its maximum value as *α* increased from 0 to 1.0 (Figure 3f). Consequently, *α* = 1.0, which yielded the maximum FOM, was determined as the optimal structural parameter for MB‐tExo‐MX biosensing. On this basis, we explored the sensing performance of the QBIC and dipole resonant modes for the optimized configuration. The QBIC resonance peak at 0.60 THz mainly originated from the electric quadrupole (EQ), whereas the dipole peak at 1.62 THz was primarily attributed to the electric dipole (ED) (Figure 3h). The intrinsic differences between these two modes arose from the distinct arrangements of surface currents (Figure 3i). Furthermore, both the magnetic and electric field intensities of the QBIC mode were markedly greater than those of the dipole mode (Figure 3i), demonstrating its stronger local field enhancement and superior sensing performance. In this mode, the MMs exhibited a distinct response to subtle changes in the dielectric constant of the surrounding medium (Figure S10a), with a sensing sensitivity of 287 GHz/RIU (Figure S10b), indicating its promising potential for sensing applications. Compared with previously reported metasurface biosensors (Table S1), the proposed QBIC MM achieved a favorable balance between the *Q* factor and sensitivity in the low‐frequency THz regime relevant to exosome sensing.

To investigate the response of the constructed THz MMs to MXene, numerical simulations were performed by tuning the coverage radius to emulate varying MXene loading densities on the metasurface. As depicted in Figure 3j, an obvious redshift in the QBIC resonance occurred after the introduction of MXene, accompanied by remarkable intensity attenuation, effects that were validated by experimental measurements (Figure 3k). Notably, single‐layer MXene was utilized in these simulations and experimental validations to achieve a balance between signal amplification and resonance retention. To quantify the variations in the THz signals of the MMs, the frequency shift and transmittance change were defined as Δ*f* = |*f*_S_ − *f*_0_| and Δ*T* = |*T*_S_ − *T*_0_|, respectively, where *f*_0_ and *T*_0_ denote the resonance frequency and transmittance of the bare MM, respectively, and *f*_S_ and *T*_S_ represent the corresponding values upon analyte introduction. The spatial uniformity of the metasurface was evaluated by measuring Δ*f* and Δ*T* at 20 randomly selected locations on a single chip. The relative variations were both within 10% (Figure 3l,m), indicating good within‐chip spatial uniformity. Intrabatch reproducibility was also evaluated using five chips from the same batch (three positions per chip), with RSDs of 2.54% for Δ*f* (Figure S11a) and 3.22% for Δ*T* (Figure S11b), confirming good intrabatch consistency and supporting the applicability of the proposed QBIC MM for MXene detection.

### 2.4 Performance of the THz Metasensor for Detecting tExos

After the QBIC MM was fabricated, the as‐synthesized MB‐tExo‐MX complex was dropped onto its surface and dried prior to THz characterization (Figure 4a). To improve tExo detection performance, the synthesis conditions of MB‐tExo‐MX, including the concentrations of anti‐GPC‐1 and anti‐ZIP4 antibodies, incubation duration, and reaction temperature, were optimized (Figure S12a–d). Under optimized experimental conditions, the THz transmission spectra of MB‐tExo‐MX with varying tExo concentrations ranging from 10^1^ to 10^9^ particles mL^−1^ were measured, as shown in Figure 4b. As the tExo concentration increased, the resonance frequency decreased, whereas the transmittance at the resonance minimum increased. Δ*f* was linearly related to the logarithm of the tExo concentration in the range of 10^3^ to 10^9^ particles mL^−1^ (Figure 4c). The weighted least‐squares linear fitting equation was Δ*f* = 4.397Lg*C* – 2.065, with a coefficient of determination R^2^ = 0.996. The LOD was 647 particles mL^−1^ on the basis of the mean blank signal plus three times its standard deviation (µ + 3σ). Moreover, Δ*T* had a favorable linear relationship with the logarithm of the tExo concentration in the range of 10^4^–10^9^ particles mL^−1^ (Figure 4d). The linear regression equation was Δ*T* = 1.388Lg*C* + 0.343 (R^2^ = 0.994), yielding a corresponding LOD of 1,010 particles mL^−1^. These results compare favorably with those of most previously reported studies (Table S2), reflecting synergistic contributions from immunomagnetic enrichment, the high THz conductivity of MXene, and the inherent advantages of fluorophore‐ and enzyme‐free THz readout: physical transduction of dielectric properties, minimal photothermal stress on exosomes, and high‐*Q* subwavelength confinement for enhanced light‒matter interactions. A systematic mechanistic comparison is provided in Table S3. Subsequently, the selectivity of the THz metasensor for PC‐derived tExos was investigated. As shown in Figure 4e,f, Δ*f* and Δ*T* for PANC‐1 exosomes were significantly greater than those for HPNE, MDA‐MB‐231, and HepG2 exosomes and the blank control. Additionally, mixing PANC‐1 exosomes with the other three types of exosomes clearly altered the Δ*f* and Δ*T*. To assess nonspecific adsorption, several controls were tested: bare MBs with tExos and MX‐aGPC‐1 probes, bare MXene with tExos and MB‐aZIP4 probes, and MB‐aZIP4 mixed with MX‐aGPC‐1 in the absence of tExos. All of these showed blank‐level Δ*f* and Δ*T* signals (Figure S13a–c), indicating negligible nonspecific adsorption. Moreover, disruption of tExos with 1% Triton X‐100 markedly attenuated both signals relative to those of intact tExos (Figure S13d–f), demonstrating that the THz response depends on vesicle integrity. These results collectively demonstrated that the THz metasensor was highly specific for PC‐derived tExos and could distinguish them from nontarget exosomes, supporting its potential for further development in clinical sample analysis. Compared with Δ*T*, Δ*f* was a better parameter for assessing the detection performance of the THz metasensor, as it represents a wider detection range and a lower LOD. Therefore, Δ*f* was adopted as the sensing parameter in subsequent experiments.

**FIGURE 4:**
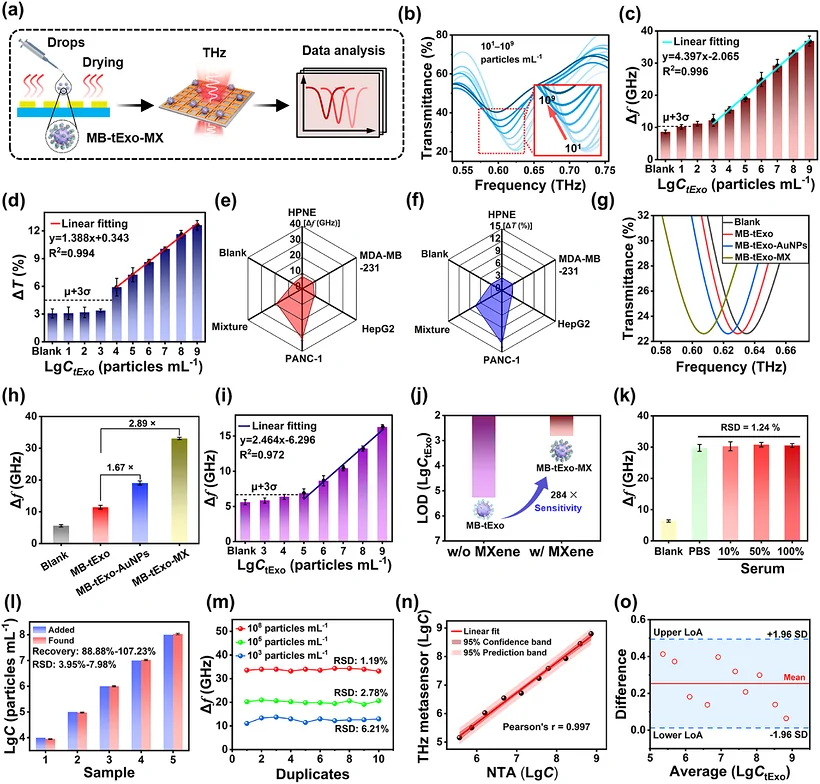
Analytical performance assessment of the THz metasensor for tExo detection. (a) Schematic diagram of the THz MM detection of MB‐tExo‐MX. (b) Measured THz transmission spectra of tExos at different concentrations. (c, d) The corresponding linear relationships between (c) Δ*f* and (d) Δ*T* and the logarithm of the tExo concentration. Data are presented as the mean ± SD of *n* = 3 replicates. (e, f) Measurement of Δ*f* and Δ*T* showing the specificity of the THz metasensor for PC‐derived tExos. Data are presented as the mean ± SD of *n* = 3 replicates. (g) Peak‐normalized THz transmission spectra of the blank control, MB‐tExo, MB‐tExo‐AuNPs, and MB‐tExo‐MX. (h) Δ*f* of the blank control, MB‐tExo, MB‐tExo‐AuNPs and MB‐tExo‐MX. Data are presented as the mean ± SD of *n* = 3 replicates. (i) The corresponding linear relationship between Δ*f* and the logarithm of the tExo concentration in the absence of MXene. Data are presented as the mean ± SD of *n* = 3 replicates. (j) LODs of tExo detection with (w/) and without (w/o) MXene. (k) Detection of tExos in different environments. Data are presented as the mean ± SD of *n* = 3 replicates. (l) Detection of tExos in spiked serum samples. Data are presented as the mean ± SD of *n* = 3 replicates. (m) Repeatability detection of tExos at three different concentrations (10^3^, 10^5^, and 10^8^ particles mL^−1^). (n) Correlations between THz metasensor results and NTA results for tExo detection. (o) Bland‒Altman analyses of the THz metasensor results and NTA results.

To verify the THz signal amplification efficacy of the MXene probes, we compared them with conventional nanoparticle‐based amplification tags. Gold nanoparticles (AuNPs) were selected as a reference nanomaterial because of their well‐established surface chemistry and electromagnetic responsiveness in biosensing. Specifically, AuNP probes were prepared from 20‐, 50‐, and 100‐nm particles using the same surface‐functionalization and antibody‐conjugation procedure, and their successful synthesis was characterized using UV–vis absorption spectroscopy, DLS, and zeta potential measurements (Figure S14a–c). Under identical assay conditions, the 50‐nm AuNP‐aGPC‐1 probe produced the greatest THz response (Figure S14d) and was therefore used as the optimized AuNP control for comparison with the MXene probes. As shown in Figure 4g,h, the Δ*f* value of MB‐tExo‐MX was 2.89 times that of MB‐tExo, which was greater than the 1.67‐fold increase achieved by MB‐tExo‐AuNPs. These findings indicated that compared with the AuNP probes, the MXene probes possessed a stronger signal amplification capability, which may be attributed to their superior electromagnetic coupling performance [58]. In contrast, the sensor without MXene probes generated much smaller frequency shifts at the same tExos concentration. As depicted in Figure 4i, Δ*f* had a linear correlation with the logarithm of the tExo concentration across a narrower range of 10^5^–10^9^ particles mL^−1^. The weighted least‐squares linear fitting equation was Δ*f* = 2.464Lg*C* − 6.296, with a coefficient of determination R^2^ = 0.972, and the LOD was calculated as 1.84 × 10^5^ particles mL^−1^. Benefiting from the outstanding signal enhancement of the MXene probes, the proposed THz metasensor demonstrates a 284‐fold reduction in the LOD (Figure 4j), which translates to substantially improved sensitivity compared with conventional nanomaterial‐based signal enhancers such as AuNPs [59] and graphene [60]. To evaluate the clinical application potential of the THz metasensor, the anti‐interference performance of the THz metasensor was analyzed by adding the same concentration of tExos into PBS and different dilutions of human serum. As shown in Figure 4k, the Δ*f* induced by tExos in the PBS system was almost identical to that in serum (10%, 50%, and 100%). This excellent anti‐interference capability arises from magnetic separation, which removes the serum matrix before the complexes are deposited onto the metasurface. Furthermore, recovery analysis was performed by spiking tExos at given concentrations into 10% serum. As displayed in Figure 4l, the recovery rate fluctuated within the narrow range of 88.88% to 107.23%, and the relative standard deviation (RSD) value of the measured concentration ranged from 3.95% to 7.98%. Parallel tests were conducted at different concentrations (10^3^, 10^5^, and 10^8^ particles mL^−1^), yielding RSD values ranging from 1.19% to 6.21% (Figure 4m), confirming that the THz metasensor provided excellent reproducibility. To assess the quantitative agreement between the THz metasensor and a reference particle‐counting method, the constructed tExo samples were measured in parallel by NTA. The THz and NTA results were strongly correlated (Pearson's r = 0.997; Figure 4n), and Bland–Altman analysis performed on log_10_‐transformed concentrations revealed a mean bias of 0.253 log_10_ units (95% CI: 0.165–0.341) between the THz metasensor and NTA, corresponding to a geometric mean NTA/THz ratio of approximately 1.79. The 95% limits of agreement ranged from 0.012 to 0.495 log_10_ units (Figure 4o), indicating good agreement between the two methods across the tested concentration range. We next compared the THz metasensor with established and emerging exosome‐detection platforms in terms of the detection limit, assay time, and operational complexity (Table S4). Relative to these platforms, the combination of ZIP4/GPC‐1 immunomagnetic enrichment with an MXene‐enhanced QBIC THz readout enabled reporter‐free detection, eliminating the need for fluorescent or enzymatic labels required by conventional immunoassays, while dual‐marker recognition improved selectivity for PC‐derived tExos. Future miniaturization of the THz device and integration with automated liquid handling will facilitate high‐throughput clinical screening.

### 2.5 Monitoring of Tumor Burden and Therapeutic Assessment With the THz Metasensor

Real‐time monitoring of tumor burden and evaluations of therapeutic efficacy have become core components of precision cancer diagnosis and treatment [61]; however, conventional detection methods are not ideal because the responses of the measured biomarkers are delayed and the procedures are highly invasive. On the basis of its excellent sensing performance, the feasibility of the THz metasensor for monitoring tumor burden and evaluating therapeutic response was investigated. As shown in Figure 5a, PC xenograft tumor (PXT) models were established by inoculating PANC‐1 cells into the right flank of BALB/c nude mice. Tumor volume and body weight were measured weekly, and blood samples were collected weekly for serum isolation. A separate cohort of healthy mice served as the healthy control group. At the fifth week post‐inoculation, tumor‐bearing mice were randomly allocated into two groups: one group received weekly intraperitoneal injections of PBS (tumor bearing group), and the other group received weekly intraperitoneal injections of cisplatin (10 mg/kg; tumor bearing & chemo group). The mice in the healthy control group received weekly intraperitoneal injections of PBS identical to those in the tumor bearing group. MRI revealed variations in tumor growth among individual mice under the different treatment regimens at the experimental endpoint (week 8) (Figure 5b). As shown in Figure 5c,d, the mean weight and volume of resected tumors in the tumor bearing chemotherapy group were significantly lower than those in the tumor bearing group. Only a slight reduction in body weight was observed in the tumor bearing & chemo group (Figure S15), and no obvious toxicity symptoms, such as drastic weight loss or abnormal behaviors, were detected. These results demonstrated the antitumor activity and tolerability of the chemotherapy regimen during the observation period, serving as important prerequisites for validating the therapeutic‐response assessment performance of the THz metasensor.

**FIGURE 5:**
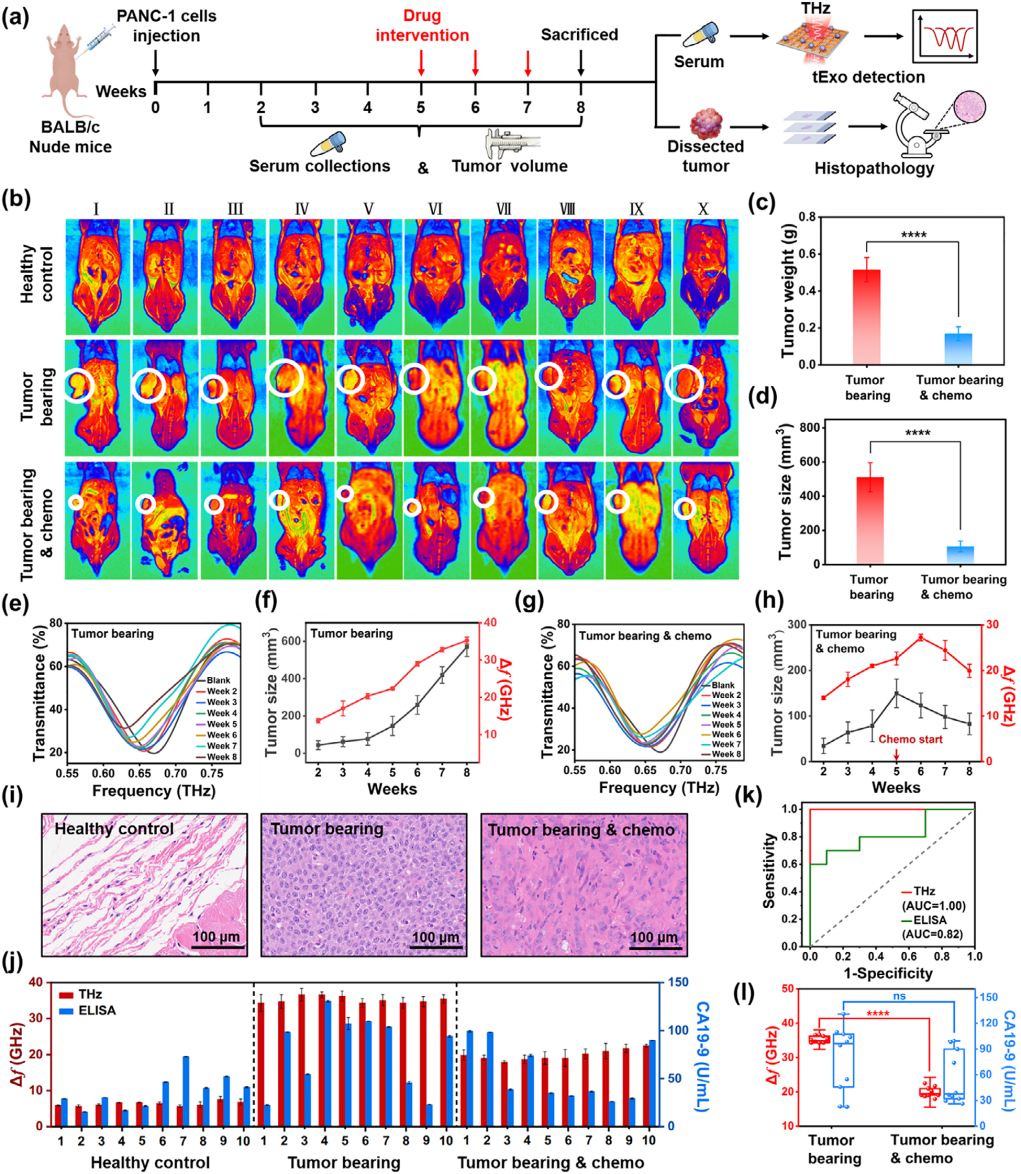
Monitoring of tumor burden and therapeutic assessment using the THz metasensor. (a) Schematic diagram of the treatment and detection protocol for the PXT mouse model. (b) MRI of each mouse in the healthy control group, tumor bearing group, and tumor bearing & chemo group at week 8 (experimental endpoint). Tumors are circled in white. (c,d) The mean tumor weight (c) and tumor volume (d) of the mice in the tumor bearing group (*n* = 10) and tumor bearing & chemo group (*n* = 10) at week 8. (e) THz transmission spectra of tumor bearing mice at different time points. (f) Relationship between tumor size and Δ*f* in tumor bearing mice over 2–8 weeks. Data are presented as the mean ± SD of *n* = 10 mice. (g) THz transmission spectra for mice in the tumor bearing & chemo group at different time points. (h) Relationship between tumor size and Δ*f* in mice in the tumor bearing & chemo group over 2–8 weeks. Data are presented as the mean ± SD of *n* = 10 mice. (i) H&E staining of subcutaneous tissues from healthy mice and tumor tissues from mice in the tumor bearing and tumor bearing & chemo groups. Scale bar: 100 µm. (j) Δ*f* and CA19‐9 levels in individual mice from the healthy control, tumor bearing, and tumor bearing & chemo groups, as measured via the THz metasensor and ELISAs, respectively. Each value represents the mean of 3 replicates per mouse, and the error bars indicate the SDs. (k) ROC curve analysis of the THz metasensor results and CA19‐9 ELISA results for discriminating tumor bearing mice from healthy mice. (l) Comparison of Δ*f* and CA19‐9 levels between mice in the tumor bearing and tumor bearing & chemo groups (*n* = 10 per group). Statistical significance in (c, d) and (l) was determined by a two‐tailed independent‐samples Student's t test (^****^ *p* < 0.0001; ns, not statistically significant).

To assess the dynamic monitoring of the tumor burden by the THz metasensor, tExos in serum samples from tumor bearing mice at different time points were detected. As shown in Figure 5e,f, the resonance frequency shift Δ*f* increased steadily with the continuous growth and progression of subcutaneous tumors in model mice (Figure S16, top), indicating that the serum tExo level increased with increasing tumor burden and that the THz metasensor accurately captured this subtle change. In contrast, tExos extracted from the serum of healthy mice triggered minimal variation in Δ*f* signals (Figure S17a,b), confirming that the THz metasensor effectively distinguished between normal physiological states and tumor states. To further elucidate the response characteristics of tExo levels to clinical chemotherapy intervention, the variations in Δ*f* after chemotherapy were recorded. As shown in Figure 5g,h, Δ*f* tended to decrease with decreasing tumor volume after chemotherapy in model mice bearing subcutaneous tumors (Figure S16, bottom). Notably, Δ*f* exhibited a transient abnormal increase one week after the initiation of chemotherapy. To investigate whether this phenomenon was associated with chemotherapy‐induced tumor cell damage, histopathological analysis was performed. Compared with those in the healthy control and tumor bearing groups, tumor cells in the tumor bearing & chemo group displayed obvious structural and morphological damage (Figure 5i), accompanied by increased apoptosis‐positive cells and cleaved caspase‐3 expression (Figure S18), confirming that chemotherapy triggered tumor cell apoptosis. We next examined whether this apoptotic response was accompanied by an increased abundance of circulating tExos. Serum‐derived exosomes were isolated from tumor bearing & chemo mice immediately before chemotherapy (week 5) and one week after treatment initiation (week 6). NTA revealed an approximately 4.80‐fold increase in the total exosome concentration (Figure S19a,c), whereas nano‐flow cytometry revealed that the GPC‐1^+^/ZIP4^+^ double‐positive population increased from 7.6% to 10.1% (Figure S19b,d), corresponding to an approximately 6.38‐fold increase in their estimated circulating concentration. These findings suggested that the transient Δ*f* increase was likely associated with chemotherapy‐induced apoptosis of tumor cells and the subsequent release of GPC‐1^+^/ZIP4^+^ tumor‐associated exosomes into the peripheral circulation [62]. Together, these results demonstrated that the THz metasensor could not only effectively monitor tumor progression but also respond rapidly to therapeutic interventions.

Finally, the detection performance of the developed THz metasensor was compared with that of the conventional CA19‐9 assay to validate its clinical translational potential. The corresponding Δ*f* values for each mouse in the healthy control group, tumor bearing group and tumor bearing & chemo group (detailed in Figures S20–S22), as well as the CA19‐9 levels measured by ELISA, are shown in Figure 5j. In this xenograft model, the THz metasensor achieved perfect discrimination between tumor bearing mice and healthy controls (AUC = 1.00), outperforming the CA19‐9 assay (AUC = 0.82) in the receiver operating characteristic (ROC) curve analysis (Figure 5k). With respect to the mice in the tumor bearing & chemo group, the Δ*f* significantly changed following therapeutic intervention, whereas no obvious alterations in CA19‐9 levels were observed (Figure 5l). These results confirmed that the THz metasensor performed better than the CA19‐9 assay in the diagnosis and therapeutic‐response assessment in this mouse model of PC.

### 2.6 Clinical Applicability of the THz Metasensor for PC Diagnosis

The results of the animal experiments validated the feasibility of the THz metasensor for longitudinal tumor monitoring under controlled xenograft conditions. Building upon this proof‐of‐concept, we assessed its clinical utility in human specimens. Serum samples from 30 healthy donors (HDs), 11 early‐stage PC patients, and 19 advanced‐stage PC patients were collected at the First Affiliated Hospital of Army Medical University; demographic and clinical information for all participants is provided in Table S5. The complete workflow of the THz metasensor for clinical sample analysis is shown in Figure 6a. The detection performance of the THz metasensor was compared with that of the ELISA‐based CA19‐9 assay. The heatmaps for the THz metasensor and ELISA signals from HDs, early‐stage PC patients and advanced‐stage PC patients are shown in Figure 6b, in which darker hues correspond to higher signal intensities; visually distinct distribution patterns of the two detection signals can be clearly observed among different populations. As illustrated in Figure 6c,g, both the Δ*f* values and CA19‐9 levels gradually increased with PC progression (as detailed in Tables S6 and S7). Statistical analyses confirmed significant differences in Δ*f* between patients with early‐stage PC and HDs, as well as between early‐ and advanced‐stage PC patients (Figure 6d). In contrast, serum CA19‐9 levels differed significantly between early‐ and advanced‐stage PC patients but did not significantly differ between early‐stage PC patients and HDs (Figure 6h). Subsequent ROC curve analysis demonstrated that the THz metasensor effectively distinguished all‐stage PC patients (*n* = 30) from HDs (*n* = 30), with an AUC of 0.997 (Figure 6e), indicating that the diagnostic performance of the THz metasensor was superior to that of the CA19‐9 assay (AUC = 0.926; Figure 6i).

**FIGURE 6:**
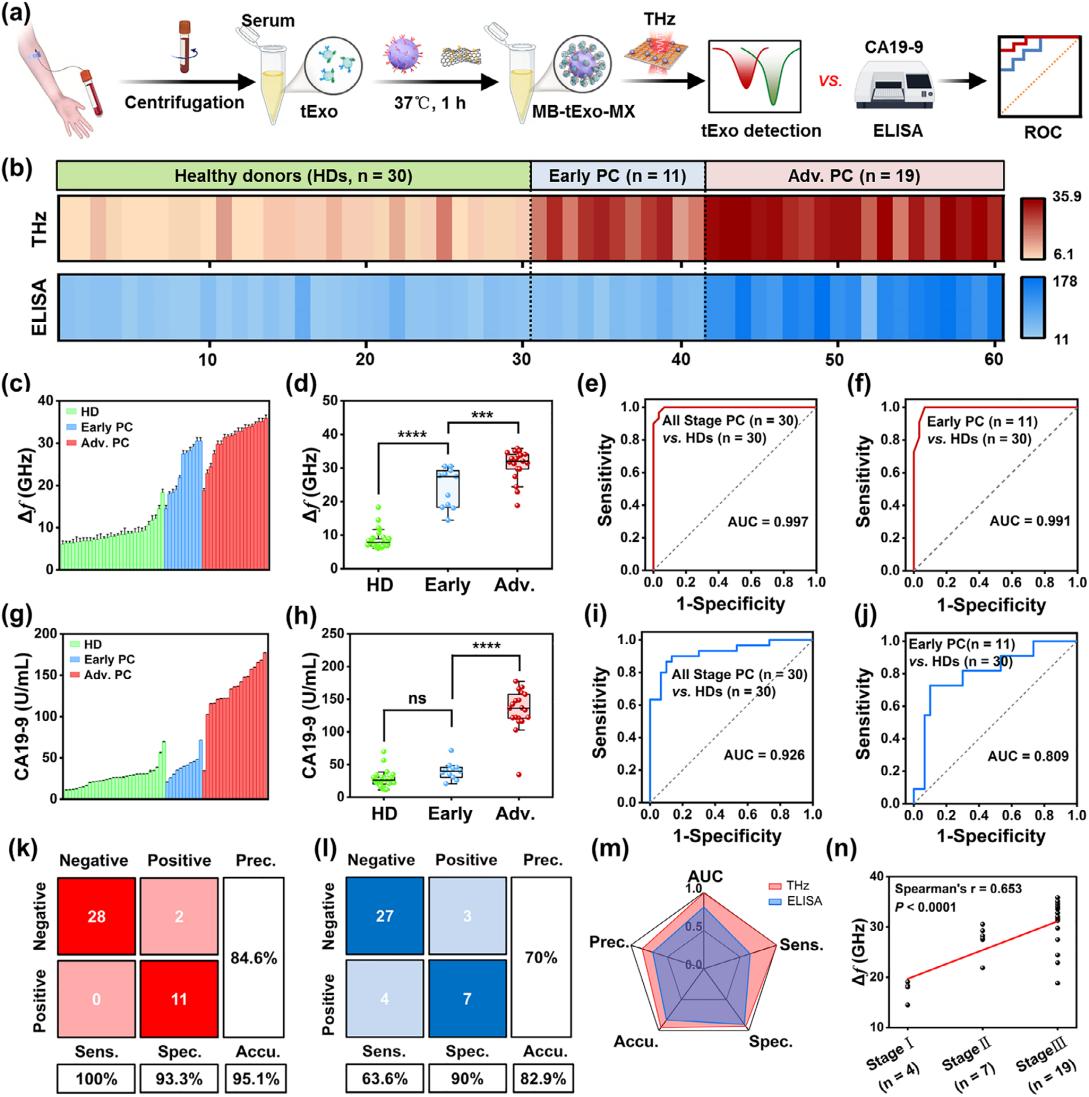
Clinical sample analysis using the THz metasensor. (a) Illustration of the procedure for analyzing clinical blood samples. (b) Representative THz and ELISA signal heatmaps of serum samples from HDs (n = 30), early‐stage PC patients (*n* = 11) and advanced‐stage PC patients (*n* = 19). (c) Δ*f* values for serum samples from HDs, early‐stage PC patients, and advanced‐stage PC patients; each value represents the mean of three replicates per sample, and error bars indicate the SDs. (d) Box plot of Δ*f*, as determined by the THz metasensor, for HDs, early‐stage PC patients, and advanced‐stage PC patients. (e) ROC curve of the THz metasensor for discriminating all‐stage PC patients from HDs. (f) ROC curve of the THz metasensor for discriminating early‐stage PC patients from HDs. (g) CA19‐9 levels in serum samples from HDs, early‐stage PC patients, and advanced‐stage PC patients; each value represents the mean of three replicates per sample, and error bars indicate the SDs. (h) Box plot of CA19‐9 levels, as determined using ELISA, in HDs, early‐stage PC patients, and advanced‐stage PC patients. (i) ROC curve of the ELISA‐based CA19‐9 assay for distinguishing all‐stage PC patients from HDs. (j) ROC curve of the ELISA‐based CA19‐9 assay for distinguishing early‐stage PC patients from HDs. (k,l) Confusion matrices of the THz metasensor (k) and ELISA‐based CA19‐9 assay (l) for the diagnosis of early‐stage PC patients. The abbreviations Sens., Spec., Accu. and Prec. stand for sensitivity, specificity, accuracy, and precision, respectively. (m) Performance comparison of the THz metasensor and the ELISA‐based CA19‐9 assay. The parameters were derived from the experimental data in this study. (n) Stage‐specific distributions of Δ*f* in PC patients with Stage I (*n* = 4), Stage II (*n* = 7), and Stage III (*n* = 19). Each point represents one individual patient, and the red line indicates the correlation trend. The association between Δ*f* and clinical stage was assessed using Spearman's rank correlation analysis. Statistical significance in (d) and (h) was determined by one‐way ANOVA followed by Tukey's post hoc test (^***^ *p* < 0.001; ^****^ *p* < 0.0001; ns, not statistically significant).

Early‐stage PC is characterized by insidious symptoms and a low tumor burden, representing the most challenging clinical diagnostic stage with a high missed diagnosis rate, which also constitutes the major limitation of conventional CA19‐9 detection [63]. For early‐stage PC (Stage I–II), the THz metasensor also demonstrated excellent distinguishing ability, with an AUC of 0.991 (Figure 6f), outperforming the CA19‐9 assay (AUC = 0.809; Figure 6j). Using a diagnostic cutoff of Δ*f* = 13.35 GHz (determined by ROC analysis with the Youden index), the THz metasensor correctly identified all 11 patients with early‐stage PC as positive, whereas only 7 patients were identified as positive using the CA19‐9 assay with a clinical cutoff of 37 U/mL. These findings indicated that the THz metasensor could effectively reduce missed diagnoses of early lesions, which is highly clinically important for improving the 5‐year survival rate of PC patients [64]. The key diagnostic indicators of the THz metasensor and CA19‐9 assay for early PC screening, including sensitivity, specificity, accuracy, and precision, are summarized in Figure 6k,l. These results indicated that the THz metasensor achieved better comprehensive diagnostic performance than the CA19‐9 assay did (Figure 6m). Beyond binary classification, we investigated whether the sensor could stratify disease severity among confirmed PC patients. The distributions of Δ*f* for PC patients with Stage I (*n* = 4), Stage II (*n* = 7), and Stage III (*n* = 19) are shown in Figure 6n. Spearman's rank correlation analysis revealed a significant positive correlation between Δ*f* and clinical stage (r = 0.653, *p* < 0.0001). Pairwise ROC analyses between adjacent stages yielded AUCs of 1.00 (Stage I vs. Stage II) and 0.786 (Stage II vs. Stage III) (Figure S23a,b). These results suggested that Δ*f* correlated with disease progression and had preliminary potential for stage‐based assessment in diagnosed PC patients, although validation in larger cohorts is needed before clinical implementation. Additionally, we compared this method with currently available biomarker detection approaches, further clarifying its advantages for clinical application. As summarized in Table S8, CEA detection uses a relatively small sample volume (50 µL); however, its low diagnostic accuracy (AUC = 0.68) greatly limits its reliability. In contrast, miRNA and ctDNA assays demonstrate relatively high diagnostic efficacy, but they are restricted by large sample demands, higher testing costs, and more complex procedures, severely hindering their widespread application in rapid clinical practice. Compared with these methods, the THz metasensor offers superior diagnostic accuracy, fewer operational steps, minimal sample consumption, and a low cost, making it a more practical tool for routine clinical screening of early PC.

## 3 Conclusion

In this study, we developed a THz metasensor that integrates dual‐probe cooperative recognition with a QBIC‐based THz metamaterial for the highly specific and ultrasensitive detection of PC‐derived tExos. Compared with AuNP probes, the MXene probes exhibited superior signal amplification capability, substantially improving the detection sensitivity with a limit of detection as low as 647 particles mL^−1^. In a PC xenograft mouse model, the THz metasensor enabled sensitive dynamic monitoring of tumor burden and therapeutic response. In clinical validation using human samples, the THz metasensor effectively distinguished patients with early‐stage PC from healthy individuals, with a diagnostic accuracy outperforming conventional serum CA19‐9 ELISA. Despite these promising results, several limitations must be addressed before clinical translation. First, while dynamic therapeutic monitoring was validated in mouse models, the current clinical data are cross‐sectional and lack longitudinal tracking during treatment. A prospective cohort with serial sampling throughout therapy is being established to validate the performance of real‐world monitoring. Second, the clinical cohort was small, single‐center, and lacked benign pancreatic disease controls (e.g., chronic pancreatitis). Large‐scale, multicenter trials incorporating such confounders are needed to confirm diagnostic robustness. Overall, our findings demonstrate that the developed THz metasensor represents a promising tool for accurate early diagnosis of PC and establishes a generalizable sensing framework that could be adapted to other cancer types upon identification and validation of corresponding dual‐specific biomarkers.

## 4 Experimental Methods

### 4.1 Materials

The reagents and instruments used in this study are described in the Supporting Information.

### 4.2 Preparation of MB‐aZIP4, AuNP‐aGPC‐1 and MX‐aGPC‐1 Probes

MB‐aZIP4 and AuNP‐aGPC‐1 were prepared via 1‐ethyl‐3‐(3‐dimethylaminopropyl) carbodiimide hydrochloride (EDC)/N‐hydroxysulfosuccinimide (Sulfo‐NHS)‐mediated covalent coupling. For MB‐aZIP4, carboxylated MBs (1 mg/mL, 100 µL) were activated with 10 mM EDC and 20 mM Sulfo‐NHS in 100 mM 2‐(N‐morpholino)ethanesulfonic acid (MES) buffer (pH 6.0) for 15 min at room temperature. After magnetic separation to remove excess reagents, the beads were incubated with 100 µL of anti‐ZIP4 antibody (500 nM) in PBS (pH 7.4) at 37°C for 4 h. For AuNP‐aGPC‐1, AuNPs (0.01 mg/mL, 100 µL) were prefunctionalized with 1 mM 11‐mercaptoundecanoic acid (MUA) in the dark at 37°C for 24 h and then centrifuged (12,000 rpm, 10 min), after which the precipitate was activated with EDC/Sulfo‐NHS following the same protocol. After centrifugation (12,000 rpm, 10 min) to remove excess activators, the product was conjugated to 100 µL of anti‐GPC‐1 antibody (200 nM). MX‐aGPC‐1 was synthesized via glutaraldehyde‐mediated Schiff base crosslinking. Briefly, MXene (0.01 mg/mL, 3 mL) was mixed with PEI (1 mg/mL, 5 µL) and 2 mL of deionized water, stirred for 1 h, and centrifuged (12,000 rpm, 10 min) to obtain MXene‐PEI; the dispersion was treated with 0.5% (v/v) glutaraldehyde for 1 h to generate surface aldehyde groups. After centrifugation (12,000 rpm, 10 min) and washing with PBS, the product was incubated with 100 µL of anti‐GPC‐1 antibody (200 nM) at 37°C for 4 h, followed by reduction with 5 mM sodium borohydride (NaBH_4_) for 30 min to stabilize covalent linkages. All probes were blocked with 1% BSA for 40 min at room temperature, washed thoroughly, and stored in PBS (pH 7.4) at 4°C until use.

### 4.3 Cell Line Culture and Exosome Isolation From Cell Culture Medium

The human pancreatic cancer cell line PANC‐1 (Beyotime Biotechnology, Shanghai, China; cat: C6725; RRID: CVCL_0480), the normal human pancreatic duct epithelial cell line HPNE (iCell Bioscience, Shanghai, China; cat: iCell‐h102; RRID: CVCL_C466), the human breast cancer cell line MDA‐MB‐231 (Beyotime Biotechnology, Shanghai, China; cat: C6550; RRID: CVCL_0062), and the human hepatoblastoma cell line HepG2 (Beyotime Biotechnology, Shanghai, China; cat: C6346; RRID: CVCL_0027) were purchased from the respective commercial suppliers. All the cell lines were authenticated by short tandem repeat (STR) profiling prior to use. Mycoplasma contamination was routinely monitored every two months using a mycoplasma detection kit according to the manufacturer's instructions, and all the cell lines were consistently confirmed to be negative. All the cell lines were cultured in Dulbecco's modified Eagle's medium (DMEM) supplemented with 10% (v/v) exosome‐depleted fetal bovine serum (FBS) and 1% (v/v) penicillin‒streptomycin in a humidified 37°C incubator with 5% CO_2_. When the cells reached 80%–90% confluence, the culture supernatant was harvested for exosome isolation via differential centrifugation. Briefly, the supernatant was first centrifuged at 300 × g for 10 min to remove dead cells, followed by centrifugation at 3,000 × g for 30 min to eliminate cell debris and apoptotic bodies. After centrifugation at 10,000 × g for 60 min to deplete large granular vesicles, the supernatant was ultracentrifuged at 100,000 × g for 70 min to obtain the exosomes. The purified exosomes were resuspended in 100 µL of 1 × PBS to prepare a stock suspension, which was stored at ‐80°C.

### 4.4 Nano‐Flow Cytometry (nFCM)

Exosomes were immuno‐stained and analyzed under standardized conditions using nFCM (Flow NanoAnalyzer, U30E). Before antibody incubation, the particle concentration of each sample was adjusted to 1.0 × 10^8^ particles mL^−1^ in PBS (100 µL) to maintain a consistent particle‐to‐antibody ratio and standardize the staining conditions across samples. Exosomes were simultaneously incubated with APC‐conjugated anti‐GPC‐1 antibody and FITC‐conjugated anti‐ZIP4 antibody for 30 min at 37°C in the dark. Unbound antibodies were removed by two consecutive rounds of ultracentrifugation, each performed at 110,000 × g for 70 min at 4°C. The pellets were gently resuspended in 50 µL of PBS. Exosomes stained with APC‐ or FITC‐conjugated isotype‐matched IgG antibodies were processed in parallel as negative controls.

### 4.5 Nanoparticle Tracking Analysis (NTA)

The isolated exosomes were appropriately diluted in 1 × PBS buffer, and their particle size and concentration were determined by using a NanoSight NS300 instrument (Malvern Panalytical, UK) equipped with NTA software version 2.3. The measurements were conducted at 25°C.

### 4.6 Western Blots

Exosome samples were lysed on ice for 30 min in radioimmunoprecipitation assay (RIPA) buffer supplemented with a protease inhibitor cocktail, and the protein concentration was determined using a BCA assay. Equal amounts of exosomal protein (20 µg per lane) were separated by sodium dodecyl sulfate‒polyacrylamide gel electrophoresis (SDS‒PAGE), followed by transfer to a polyvinylidene fluoride (PVDF) membrane. The membranes were blocked with 5% nonfat dry milk in Tris‐buffered saline containing Tween‐20 (TBST) for 1 h at room temperature. The blocked membranes were then incubated overnight at 4°C with primary antibodies targeting GPC‐1, ZIP4, CD9, CD63, CD81, TSG101, and calnexin. Detailed information on these antibodies is provided in Table S9. After three 10‐min washes with TBST buffer, the membranes were incubated with the corresponding horseradish peroxidase (HRP)‐conjugated secondary antibodies for 1 h at room temperature, followed by three additional 10‐min washes with TBST buffer. The protein bands were visualized using an enhanced chemiluminescence (ECL) substrate and imaged with a Tanon 5200 imaging system (Tanon, Shanghai, China).

### 4.7 Numerical Simulation

Numerical calculations were carried out using the finite integration technique (FIT) of the time‐domain solver CST Microwave Studio. The parameter settings of the QBIC MM and modeling of MXene are provided in the Supporting Information (SI).

### 4.8 THz Spectroscopy System Measurements

Aliquots of MB‐aZIP4 and MX‐aGPC‐1 probes (100 µL each) were mixed with 10 µL of tExo‐containing solution and incubated at 37°C for 1 h. The resulting complexes were isolated by magnetic separation and resuspended in 50 µL of deionized water. Subsequently, 20 µL of the suspension was dropped onto the metasurface and dried before THz analysis. All measurements were conducted in transmission mode using a THz time‐domain spectroscopy system (TAS7500SP; Advantest Co., Tokyo, Japan) over an effective bandwidth of 0.1–4.0 THz, with air used as the reference. The blank control was processed identically in the absence of tExos. Each sample was measured three times in transmission mode. To ensure dependable sensing results and mitigate the effects of the surrounding environment, an air dryer was used to dry the experimental environment, ensuring that the relative humidity remained below 5%.

### 4.9 Establishment of the Mouse Model of PC

Male BALB/c nude mice aged 6–8 weeks (weighing 18–22 g) were purchased from Charles River Laboratories and housed in individually ventilated cages. The rearing environment was controlled at 23°C–25°C, 40%–50% relative humidity, and a light–dark cycle of 12 h light/12 h dark. A PC xenograft model was established by subcutaneous inoculation of 100 µL of PBS containing 2 × 10^6^ PANC‐1 cells into the right flank of mice. A separate cohort of healthy mice (n = 10) served as the healthy control group and received a subcutaneous injection of 100 µL of PBS without tumor cells. The tumor volume was calculated according to the formula V = (L × W^2^)/2. At the fifth week post‐inoculation, the tumor‐bearing mice were randomly allocated into two groups: the tumor bearing group (*n* = 10; weekly intraperitoneal injection of PBS) and the tumor bearing & chemo group (*n* = 10; weekly intraperitoneal injection of 10 mg/kg cisplatin). Mice in the healthy control group received weekly intraperitoneal injections of PBS identical to those in the tumor bearing group. Humane endpoints were predefined as follows: tumor volume exceeding 2,000 mm^3^, tumor ulceration, body weight loss > 20% relative to the initial weight, or significant signs of distress (e.g., lethargy or inability to eat or drink). Mice that reached any humane endpoint were euthanized immediately by CO_2_ inhalation followed by cervical dislocation. At the end of week 8 (the experimental endpoint), all the mice were euthanized by CO_2_ inhalation followed by cervical dislocation for subsequent analysis. All animal experiments were conducted in strict accordance with the laboratory animal care and ethical guidelines of Army Medical University.

### 4.10 Magnetic Resonance Imaging (MRI) of Mice

MRI was performed using an Aspect M7 small‐animal MRI system (Aspect Imaging) operating at 1.0 T and equipped with a mouse body coil (inner diameter ≥ 30 mm, length ≥ 50 mm) for radiofrequency transmission and signal reception. During imaging, the animals were maintained under gas anesthesia and placed in the prone position, with the head secured using a tooth bar to minimize motion artifacts. Respiration was continuously monitored, and body temperature was maintained at 36°C–37°C using a warm‐air heating system. T2‐weighted images were acquired in the coronal plane using a two‐dimensional fast spin‒echo (2D FSE) sequence with the following parameters: echo time (TE) = 84 ms, repetition time (TR) = 3500 ms, field of view (FOV) = 39.37 mm × 71.46 mm, matrix = 160 × 160, slice thickness = 1.2 mm with a slice interval of 3.0 mm, and number of excitations (NEX) = 10.

### 4.11 ELISA

Human CA19‐9 was quantified using a commercial ELISA kit in accordance with the manufacturer's protocols. Gradient standard samples and prepared serum samples were added to microplate wells and incubated at 37°C for 60 min. After the supernatant of each well was removed, 100 µL of biotinylated detection antibody working solution was added, followed by incubation at 37°C for another 60 min. Then, each well was washed three times, and 100 µL of HRP‐conjugate reagent was added, followed by incubation at 37°C for 30 min. After the solution was discarded, each well was washed five times. Subsequently, 90 µL of TMB substrate was added to each well and incubated at 37°C for 15 min in the dark. Finally, the reaction was terminated by the addition of 50 µL of stop solution to each well, and the optical density (OD) at 450 nm was immediately measured using a microplate reader.

### 4.12 Ethics Statement

The animal study underwent rigorous review by the Ethics Committee of Army Medical University, China, and was approved (ethics number: AMUWEC20252039). The collection of human serum samples during this study strictly adhered to ethical standards and relevant laws and regulations. Our study was approved by the Ethics Committee of the First Affiliated Hospital of Army Medical University (approval no. (A)KY2025239). All the samples were residual blood samples collected after routine clinical testing, with no interference with their original diagnostic purpose. Written informed consent was obtained from each participant.

### 4.13 Statistical Analysis

All the quantitative data are presented as the mean ± standard deviation (SD) of at least three independent measurements. Comparisons between two groups were performed using a two‐tailed independent‐samples Student's t test, and comparisons among three groups were performed using one‐way analysis of variance (ANOVA) followed by Tukey's post hoc test. The correlation between Δ*f* and the PC stage was assessed using Spearman's rank correlation coefficient, and the correlation between the THz metasensor and the NTA results was determined using Pearson's correlation coefficient, with agreement between the two methods evaluated by Bland–Altman analysis. Receiver operating characteristic (ROC) curve analysis was performed to assess diagnostic performance. All the statistical analyses were conducted using SPSS 22.0 (IBM Corp., Armonk, NY, USA). Statistical significance was defined as *p* < 0.05. Significance levels are denoted as ^*^ *p* < 0.05, ^**^ *p* < 0.01, ^***^ *p* < 0.001, and ^****^ *p* < 0.0001; ns, not statistically significant.

### 4.14 Schematic Illustrations

All schematic illustrations and the Table of Contents graphic were prepared using Microsoft PowerPoint and Blender (v4.0).

## Author Contributions

Weidong Jin and Jiyue Chen contributed equally to this work. Weidong Jin: Conceptualization, Data curation, Investigation, Methodology, and Writing – original draft. Jiyue Chen: Formal analysis, Validation, and Writing – original draft. Fengxin Xie: Data curation. Huiyan Tian: Investigation. Xuechen Dou: Formal analysis. Yanqi Han: Formal analysis. Lu Zhang: Formal analysis. Jining Li: Conceptualization, Supervision, and Writing – review & editing. Xiang Yang: Conceptualization, Funding acquisition, Project administration, and Writing – review & editing.

## Conflicts of Interest

The authors declare no conflicts of interest.

## Use of Generative AI and AI‐Assisted Technologies in the Writing Process

During the preparation of this work, the authors used Kimi for language polishing. After using this tool, the authors reviewed and edited the content as needed and take full responsibility for the content of the publication.

## Supporting information




**Supporting File**: advs78093‐sup‐0001‐SuppMat.pdf.

## Data Availability

The data that support the findings of this study are available from the corresponding author upon reasonable request.
