# Supplementary material for: Immunomagnetic‐MXene Bifunctional Probes for Ultrasensitive and Specific Terahertz Metasensing of Tumor‐Derived Exosomes
Source: Adv Sci (Weinh). 2026 Sep 30:e78093. Online ahead of print. doi: 10.1002/advs.78093 (PMC13626167; doi:10.1002/advs.78093)
Supplement: Supplementary file 1 [file ADVS-9999-e78093-s001.pdf]

**Supporting Information**

# Immunomagnetic-MXene Bifunctional Probes for Ultrasensitive and Specific Terahertz Metasensing of Tumor-Derived Exosomes

Weidong Jin[1#], Jiyue Chen[2#], Fengxin Xie[1], Huiyan Tian[1], Xuechen Dou[1], Yanqi Han[1], Lu Zhang[1], Jining Li[2*], and Xiang Yang[1*]

[1] Department of Laboratory Medicine, Southwest Hospital, Army Medical University (Third Military Medical University), Chongqing 400038, China.

[2] Institute of Laser and Optoelectronics, School of Precision Instrument and Optoelectronics Engineering, Tianjin University, Tianjin 300072, China.

## Corresponding Authors:

* Jining Li; E-mail: jiningli@tju.edu.cn

* Xiang Yang; E-mail: yangxiang@tmmu.edu.cn

## Author Contributions:

[#] Weidong Jin and Jiyue Chen contributed equally to this work.

## Reagents and Materials

Carboxylated magnetic beads (cat: 65011) were purchased from Invitrogen (Carlsbad, CA, USA). $Ti_3C_2$ MXene nanosheets (cat: 102654) were purchased from XFNANO (Nanjing, China). Gold nanoparticles (cat: JK-02-001) were purchased from Jike Biotechnology (Nanjing, China). The anti-GPC-1 antibody (cat: ab199343; RRID: AB_3676756), APC-conjugated anti-GPC-1 antibody (cat: ab319305), anti-CD9 antibody (cat: ab236630; RRID: AB_2922400), anti-CD63 antibody (cat: ab134045; RRID: AB_2800495), anti-CD81 antibody (cat: ab109201; RRID: AB_10866464), anti-TSG101 antibody (cat: ab125011; RRID: AB_10974262) and anti-calnexin antibody (cat: ab112995; RRID: AB_10860712) were purchased from Abcam (Cambridge, UK). An anti-ZIP4 antibody (cat: 20625-1-AP; RRID: AB_10700008) was purchased from Proteintech Group, Inc. (Chicago, IL, USA). A FITC-conjugated anti-ZIP4 antibody (cat: CSB-PA747413LC01HU) was obtained from CUSABIO Technology (Wuhan, China). Bovine serum albumin (BSA, fraction V; cat: 4240GR100) was purchased from BioFROXX (Einhausen, Germany). Dulbecco's modified Eagle's medium (DMEM; cat: C11995500BT) and phosphate-buffered saline (PBS; cat: C10010500BT) were purchased from Gibco (Waltham, MA, USA). Exosome-depleted fetal bovine serum (cat: UR50202) was purchased from Umibio Bioscience Inc. (Shanghai, China). A Mycoplasma Detection Kit (cat: C0296) was purchased from Beyotime Biotechnology (Shanghai, China). 1-(3-(Dimethylamino) propyl)-3-ethylcarbodiimide hydrochloride (EDC; cat: C600433) was purchased from Sangon Biotech (Shanghai, China). N-Hydroxysulfosuccinimide (Sulfo-NHS; cat: N1500148), polyethyleneimine (PEI; cat: E498565), 11-mercaptoundecanoic acid (MUA; cat: W137344), 2-(N-morpholino) ethanesulfonic acid (MES; cat: M163013), glutaraldehyde (cat: G105906) and $NaBH_4$ (cat: S432204) were all purchased from Aladdin Biochemical Technology (Shanghai, China). Cisplatin (cat: BS27371) was purchased from Chongqing Boer Biotechnology (Chongqing, China). Normal human serum (cat: 100-512) was obtained from GeminiBio (West Sacramento, CA, USA). A human CA19-9 ELISA kit (cat: JL15203) was purchased from Jianglai Biotechnology (Shanghai, China).

## Instrumentation

The morphologies of the exosomes and MXene nanosheets were characterized using a Talos F200S transmission electron microscope (FEI, USA), and energy-dispersive X-ray spectroscopy (EDS) elemental mapping of the MB-tExo-MX complexes was performed on the same instrument operated in scanning transmission electron microscopy (STEM) mode. Atomic force microscopy measurements were performed using a Dimension ICON instrument (Bruker, USA) to determine the thickness of the MXene nanosheets. Fourier transform infrared spectroscopy (Nicolet iS20; Thermo Fisher Scientific, USA) was used to characterize the PEI coating on the MXene surface, and X-ray photoelectron spectroscopy (K-Alpha; Thermo Fisher Scientific, USA) was used to verify the subsequent antibody functionalization. The

hydrodynamic diameter and zeta potential were measured using a Zetasizer Nano ZSP instrument (Malvern Panalytical, UK) to evaluate antibody functionalization on the MB surface. Scanning electron microscopy images of MBs, MB-tExos, and MB-tExo-MX complexes were acquired using a Sigma 360 scanning electron microscope (Zeiss, Germany). Metamaterials were fabricated using a Discovery 14 magnetron sputtering system (Denton, USA) and an MA8 contact lithography system (SUSS MicroTec, Germany). Terahertz time-domain spectroscopy measurements were performed using a TAS7500SP system (Advantest, Tokyo, Japan).

**Parameter Setting of the QBIC MM and Modeling MXene in the Numerical Simulation**

Identical boundary conditions were applied throughout the simulations, with periodic boundaries defined along the x and y axis and perfectly matched layer (PML) domains arranged along the z axis to accommodate incident wave propagation. In the selection of simulation materials, structural gold was directly adopted from the software material library, which has an electrical conductivity of $4.561 \times 10^7$ S $m^{-1}$. The substrate material was fused quartz, which was also sourced from the software material library, and has a relative dielectric constant of 3.75 and is lossless. The multipole expansions were calculated with the following formula:

$$ED: p_\alpha = -\frac{1}{i\omega}\left\{\int d^3\,\boldsymbol{r}\boldsymbol{J}_\alpha^\omega j_0(kr) + \frac{k^2}{2}\int d^3\,\boldsymbol{r}[3(\boldsymbol{r}\cdot\boldsymbol{J}_\omega)r_\alpha - r^2 J_\alpha^\omega]\frac{j_2(kr)}{(kr)^2}\right\} \quad (1)$$

$$MD: m_a = \frac{3}{2}\int d^3\,\boldsymbol{r}(\boldsymbol{r}\times\boldsymbol{J}_\omega)_\alpha \frac{j_1(kr)}{kr} \quad (2)$$

$$EQ: Q_{\alpha\beta}^e = -\frac{3}{i\omega}\left\{\int\left[3\left(r_\beta J_\alpha^\omega + r_\alpha J_\beta^\omega\right) - 2(\boldsymbol{r}\cdot\boldsymbol{J}_\omega)\delta_{\alpha\beta}\right]\frac{j_1(kr)}{kr}d^3\boldsymbol{r} + 2k^2\int\left[5r_\alpha r_\beta(\boldsymbol{r}\cdot\boldsymbol{J}_\omega) - \left(r_\alpha J_\beta + r_\beta J_\alpha\right)r^2 - r^2(\boldsymbol{r}\cdot\boldsymbol{J}_\omega)\delta_{\alpha\beta}\right]\frac{j_3(kr)}{(kr)^3}d^3\boldsymbol{r}\right\} \quad (3)$$

$$MQ: Q_{\alpha\beta}^m = 15\int d^3\,\boldsymbol{r}\left\{r_\alpha(\boldsymbol{r}\times\boldsymbol{J}_\omega)_\beta + r_\beta(\boldsymbol{r}\times\boldsymbol{J}_\omega)_\alpha\right\}\frac{j_2(kr)}{(kr)^2} \quad (4)$$

The THz conductivity of MXene can be phenomenologically described using a Drude [1] response:

$$\sigma_v(\omega) = \frac{\varepsilon_0\omega_p^2\tau}{1-i\omega\tau} \quad (5)$$

where $\sigma_v(\omega)$ is the complex volumetric conductivity, $\omega_p$ is the effective plasma frequency, $\tau$ is the carrier relaxation time, and $\omega$ is the angular frequency.

For an ultrathin conductive film, its electromagnetic response can equivalently be represented by a surface current characterized by the sheet conductivity:

$$\sigma_s(\omega) = t\sigma_v(\omega) \quad (6)$$

where $t$ denotes the effective film thickness.

Accordingly, the Drude sheet conductivity can be written as:

$$\sigma_s(\omega) = \frac{\sigma_{s,0}}{1-i\omega\tau} \quad (7)$$

where $\sigma_{s,0} = t\varepsilon_0\omega_p^2\tau$ represents the zero-frequency sheet conductivity.

The experimentally deposited MXene layer has a thickness of approximately 2 nm, which is several orders of magnitude smaller than the terahertz wavelength. Therefore, it was treated in the electromagnetic simulations as an infinitesimally thin conductive sheet.

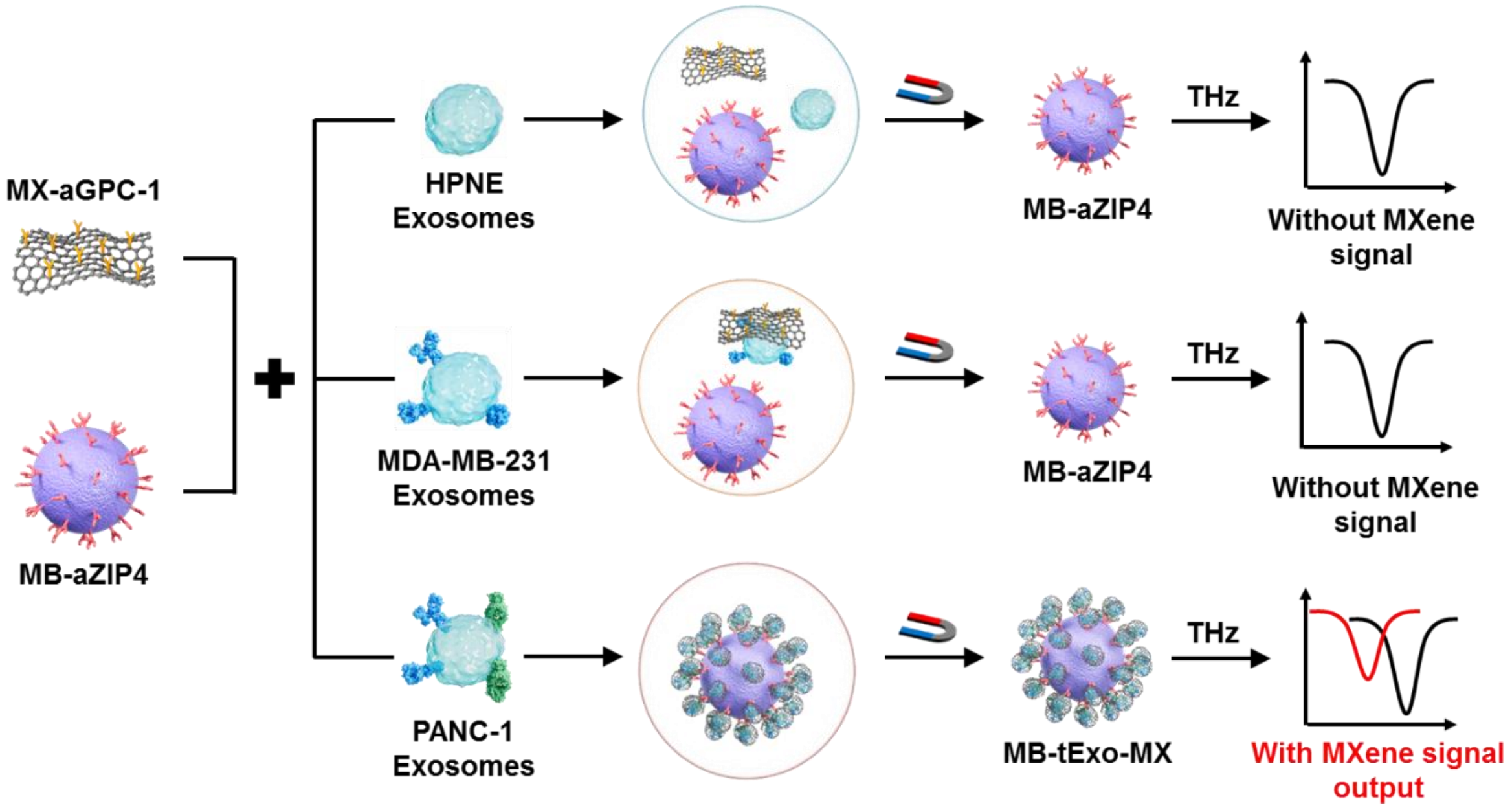


Figure S1. Schematic illustration of the dual-probe strategy for discriminating PANC-1 cell-derived exosomes from MDA-MB-231 and HPNE cell-derived exosomes. HPNE cell-derived exosomes express neither GPC-1 nor ZIP4; thus they cannot bind to MXene probes or be captured by MB probes for magnetic enrichment, resulting in no detectable MXene-derived THz signal. MDA-MB-231 cell-derived exosomes express only GPC-1; they can bind to MXene probes but cannot be recovered by magnetic separation and thus produce no measurable MXene-associated THz response. In contrast, PANC-1 cell-derived exosomes coexpress GPC-1 and ZIP4, which enables simultaneous capture by both probes to form MB-tExo-MX sandwich complexes. These complexes are retained after magnetic enrichment and generate pronounced MXene-characteristic THz signals. MX: MXene; MB: magnetic bead; tExo: tumor-derived exosome.

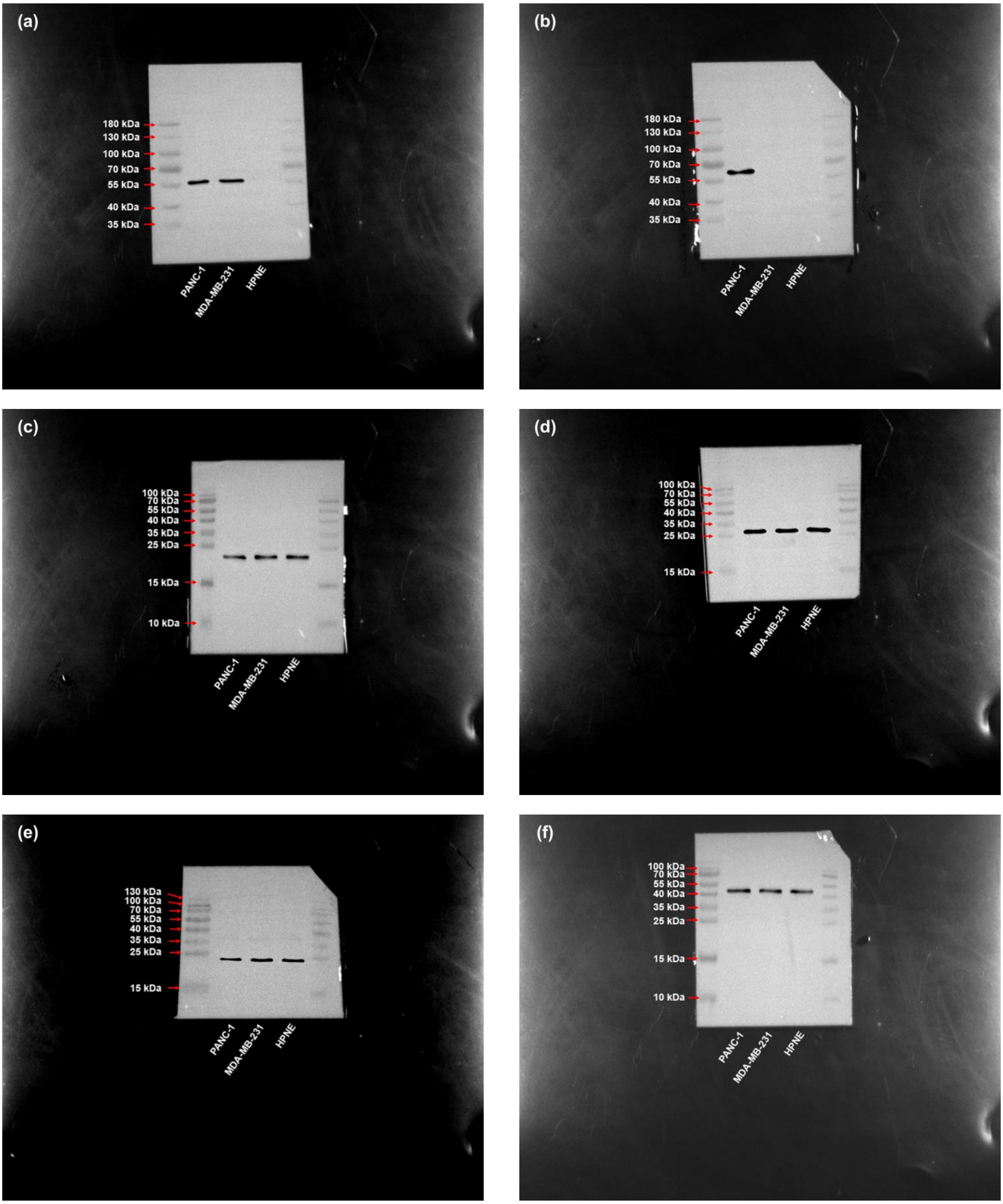

(a)
180 kDa
130 kDa
100 kDa
70 kDa
55 kDa
40 kDa
35 kDa
PANC-1
MDA-MB-231
HPNE
(b)
180 kDa
130 kDa
100 kDa
70 kDa
55 kDa
40 kDa
35 kDa
PANC-1
MDA-MB-231
HPNE
(c)
100 kDa
70 kDa
55 kDa
40 kDa
35 kDa
25 kDa
15 kDa
10 kDa
PANC-1
MDA-MB-231
HPNE
(d)
100 kDa
70 kDa
55 kDa
40 kDa
35 kDa
25 kDa
15 kDa
PANC-1
MDA-MB-231
HPNE
(e)
130 kDa
100 kDa
70 kDa
55 kDa
40 kDa
35 kDa
25 kDa
15 kDa
PANC-1
MDA-MB-231
HPNE
(f)
100 kDa
70 kDa
55 kDa
40 kDa
35 kDa
25 kDa
15 kDa
10 kDa
PANC-1
MDA-MB-231
HPNE

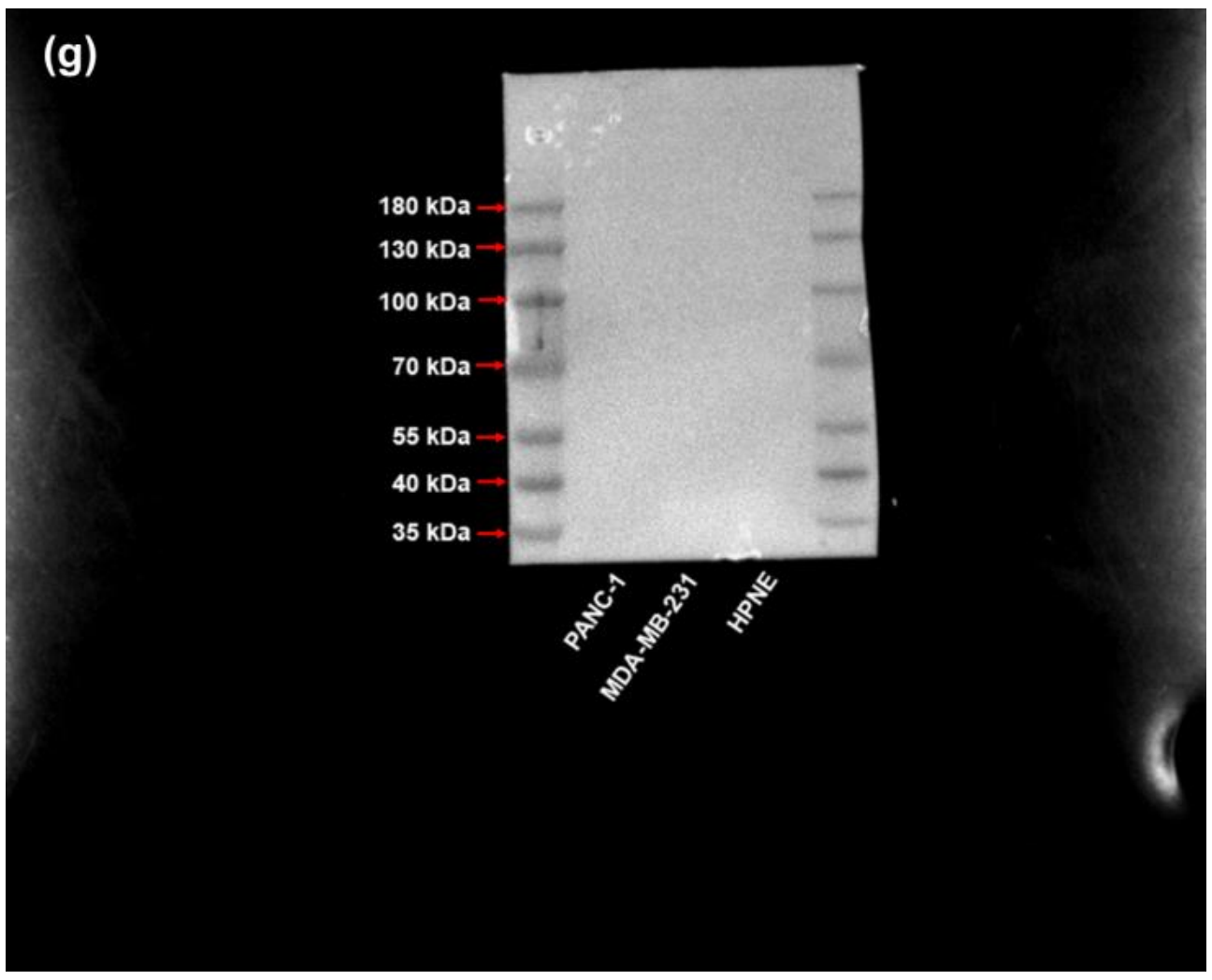


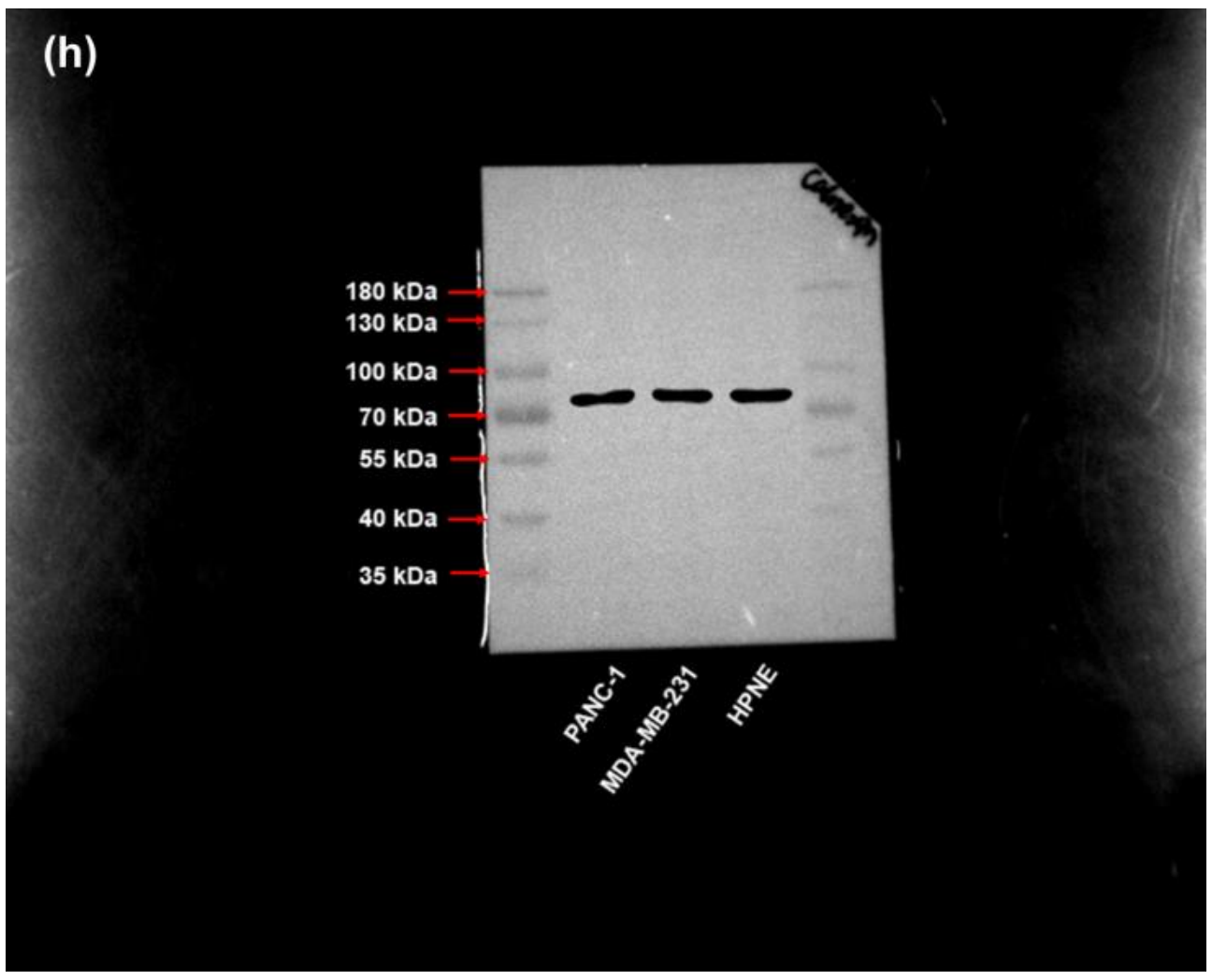


Figure S2. Uncropped Western blot images for (a) GPC-1, (b) ZIP4, (c) CD9, (d) CD63, (e) CD81, (f) TSG101 and (g) calnexin levels in exosomes derived from PANC-1, MDA-MB-231 and HPNE cells. (h) Calnexin levels in whole-cell lysates of PANC-1, MDA-MB-231 and HPNE cells. Molecular weight markers (kDa) are shown on the left.

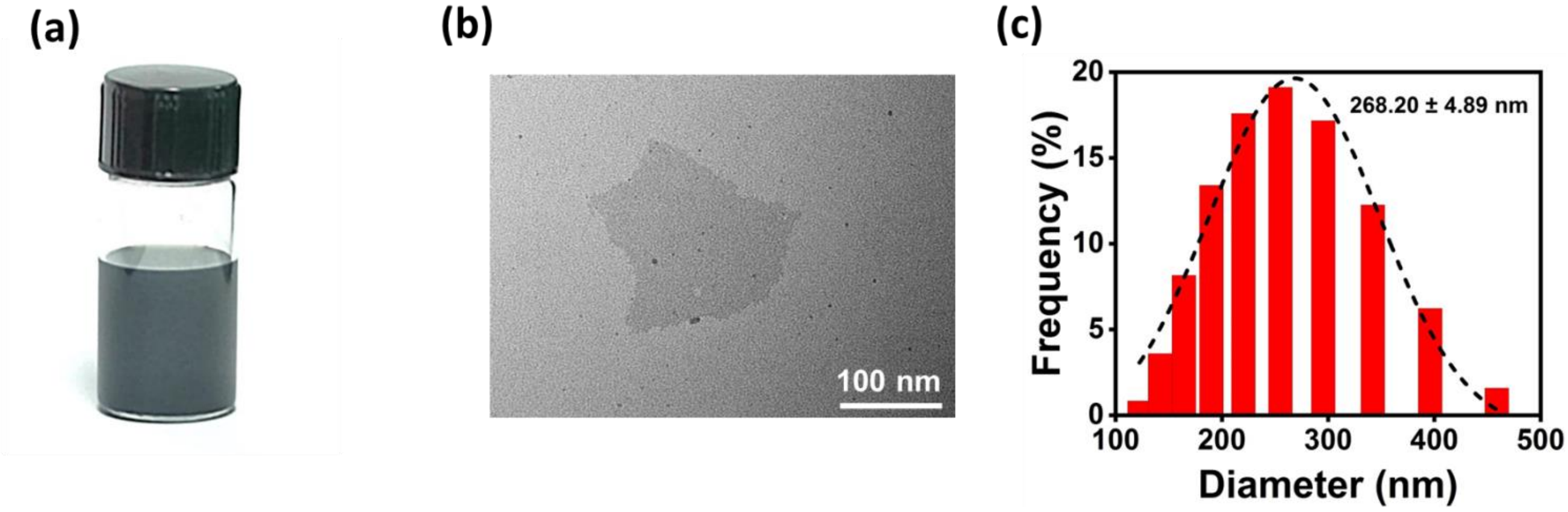


Figure S3. (a) Digital photograph of $Ti_3C_2$ MXene in aqueous solution. (b) TEM image of an MXene nanosheet. Scale bar: 100 nm. (c) DLS data of $Ti_3C_2$ MXene nanosheets.

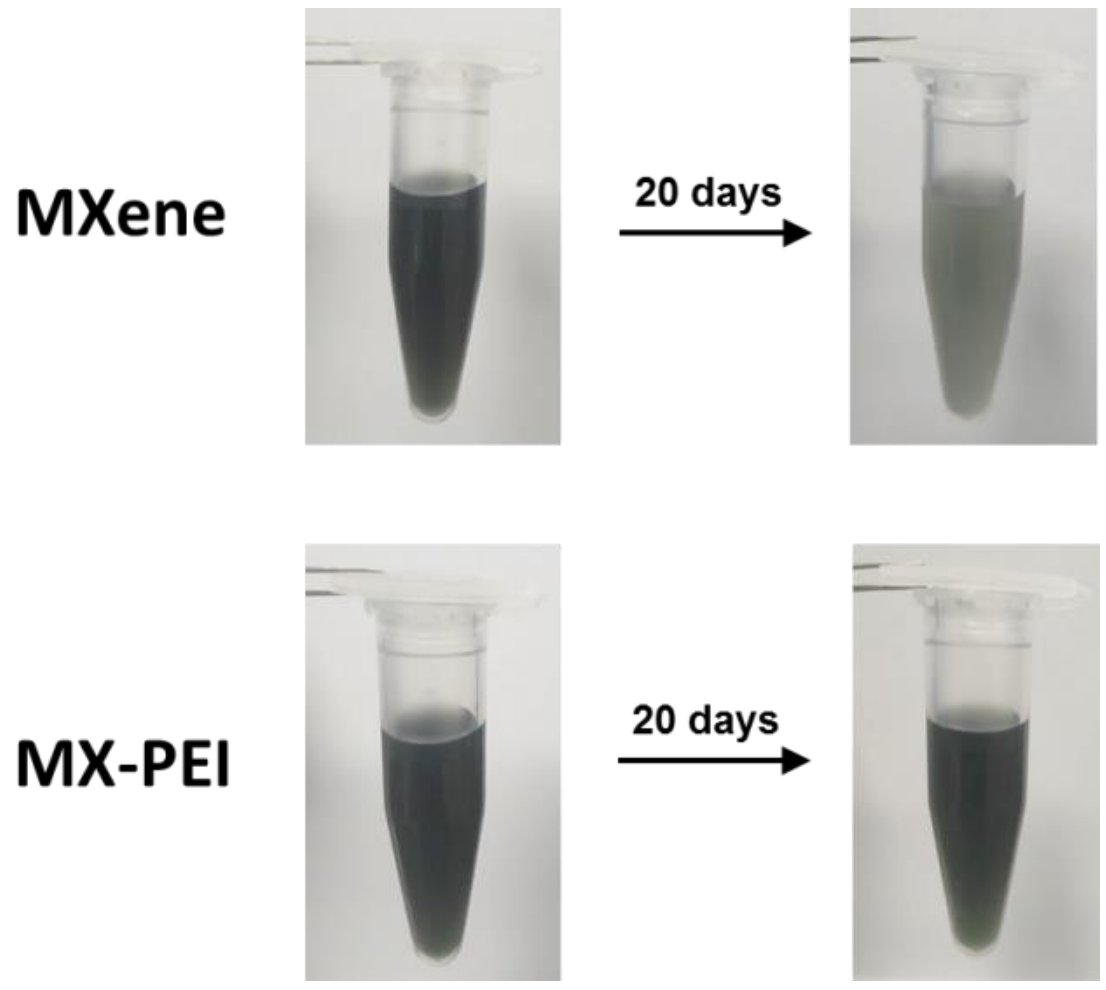

Figure S4. Photographs of pristine MXene (top) and MX-PEI (bottom) aqueous dispersions before and after storage at room temperature for 20 days.

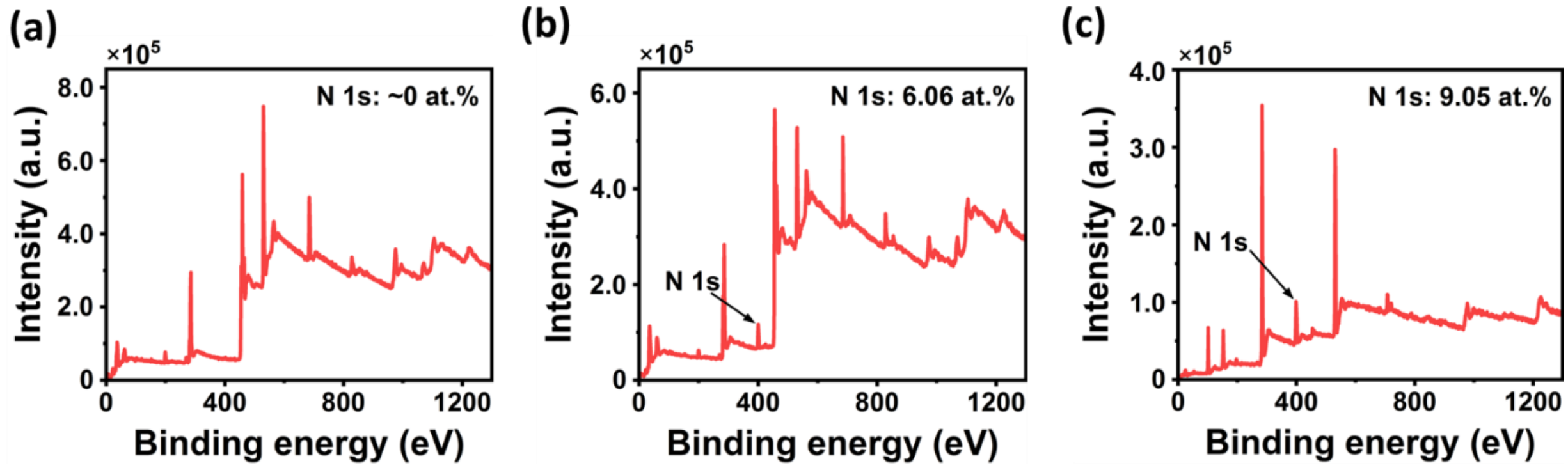


Figure S5. Wide-scan XPS survey spectra of (a) pristine MXene, (b) MX-PEI, and (c) MX-aGPC-1. The arrows represent the characteristic peaks of N 1s.

(a)

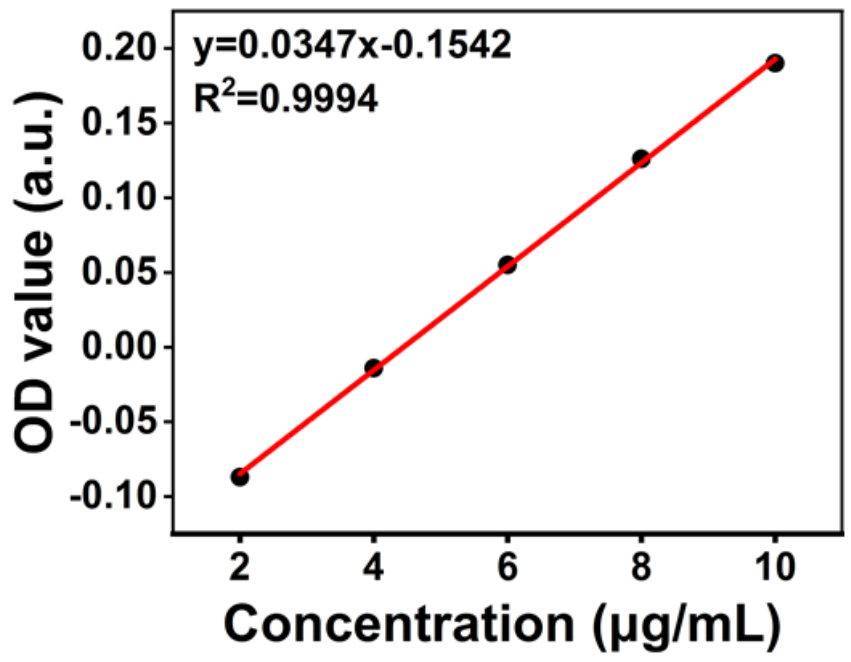


(b)

| GPC-1 antibody | |
|---|---|
| Added (μg) | 3.00 |
| Loading (μg) | 2.50 |
| Binding rate | 83.33% |

(c)

| ZIP4 antibody | |
|---|---|
| Added (μg) | 7.50 |
| Loading (μg) | 6.85 |
| Binding rate | 91.32% |

Figure S6. (a) Standard curve of total protein. Quantitative evaluation of the antibody was conducted on the basis of the standard curve of total protein measured using a BCA protein assay kit. (b) The binding ratio of the anti-GPC-1 antibody to the MXene probes. (c) The binding ratio of the anti-ZIP4 antibody to the MB probes.

(a) (b) (c)

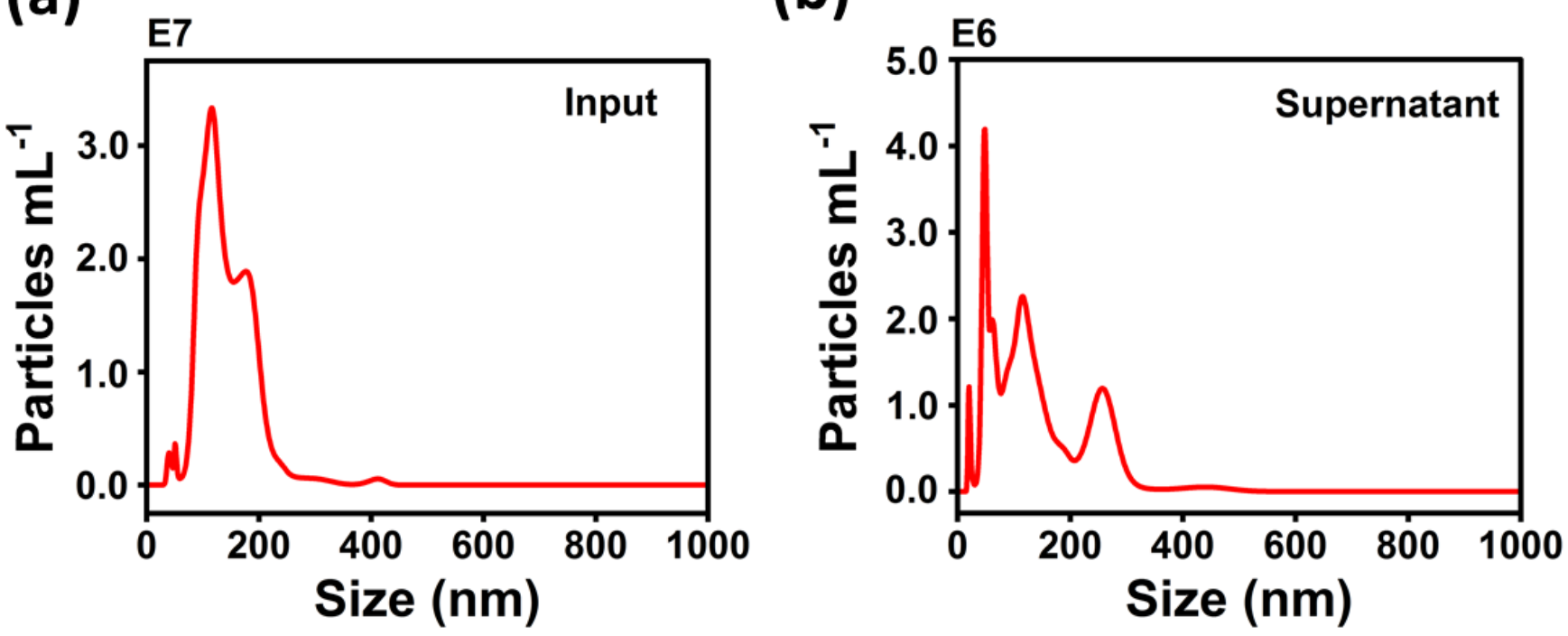


| Sample | Concentration (particles mL$^{-1}$) |
| --- | --- |
| Input | $1.67 \times 10^8$ |
| Supernatant | $4.17 \times 10^7$ |
| **Capture efficiency** | **75.03 %** |

Figure S7. Quantification of the immunomagnetic capture efficiency of MB-aZIP4 probes. (a, b) Representative NTA profiles of the initial input PANC-1 exosomes (a) and the postcapture supernatant (b) after incubation with MB-aZIP4 probes and magnetic separation. (c) Calculated capture efficiency, defined as $(C_{\mathrm{Input}} - C_{\mathrm{Supernatant}})/C_{\mathrm{Input}} \times 100\%$, where $C_{\mathrm{Input}}$ and $C_{\mathrm{Supernatant}}$ denote the input exosome concentration and the exosome concentration remaining in the supernatant, respectively. The input and supernatant PANC-1 exosomes were quantified by NTA on the same volume.

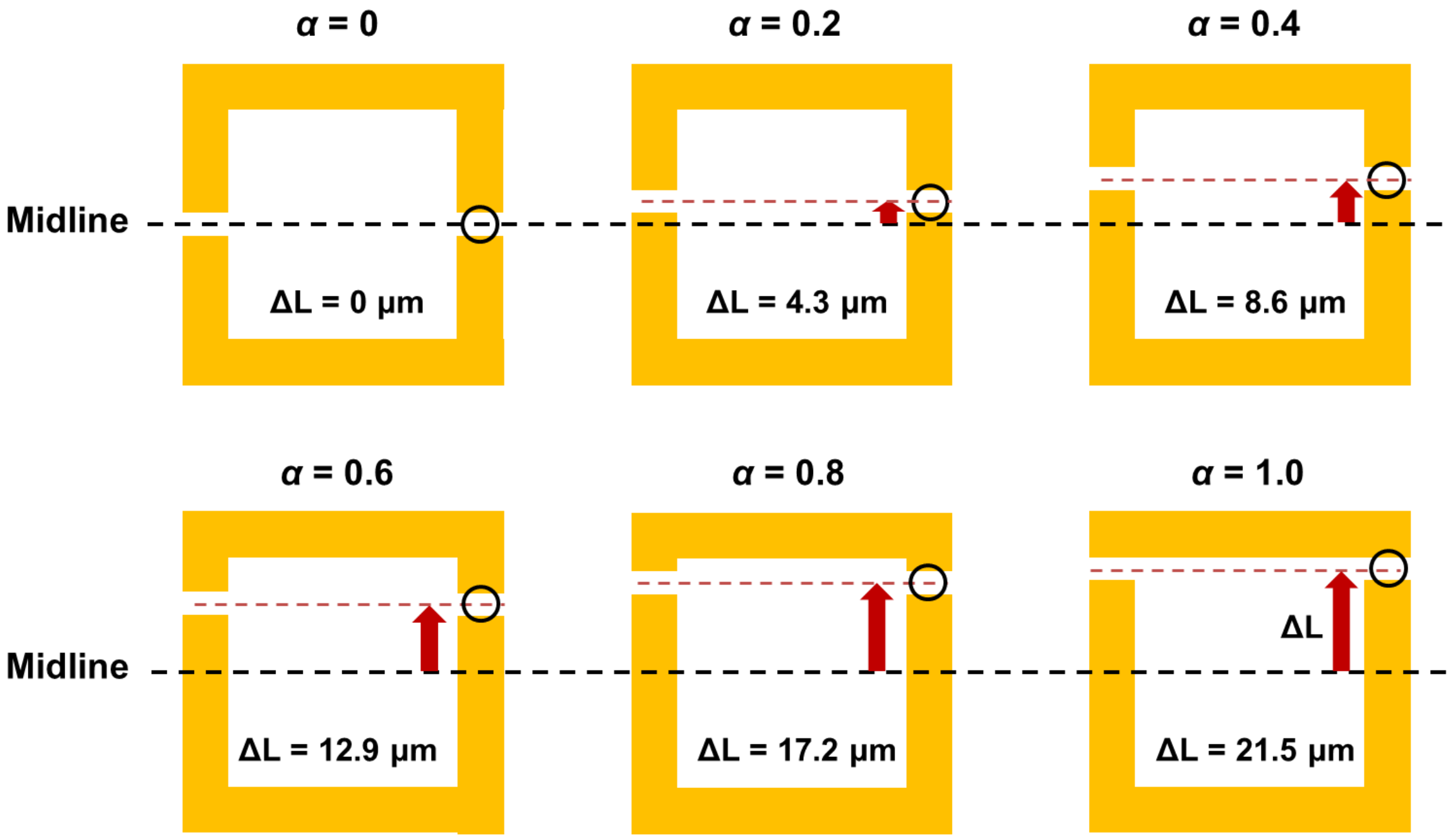


Figure S8. Schematic diagram of the asymmetry parameter $\alpha$ regulated by adjusting the distance ($\Delta L$) between the split and the midline. The degree of asymmetry $\alpha$ increases gradually with the increase in $\Delta L$.

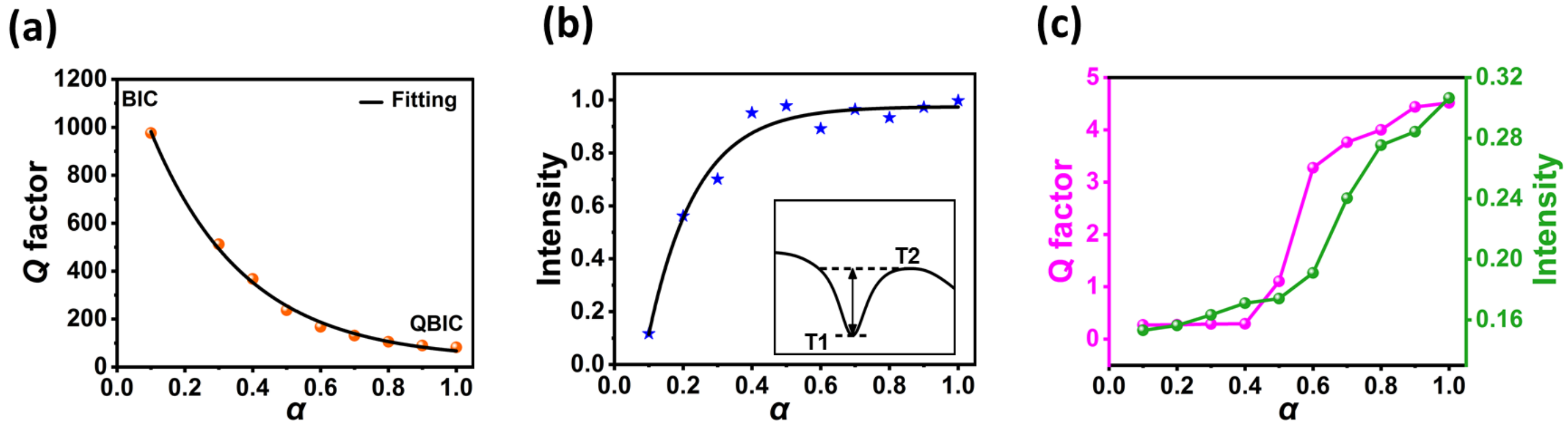


Figure S9. (a) Simulated $Q$ factors of the QBIC mode at different asymmetric parameters $\alpha$. (b) Calculated resonance intensity I of QBIC modes corresponding to different asymmetry parameter $\alpha$ values, where $\mathrm{I} = |\mathrm{T1} - \mathrm{T2}|$. (c) Simulated $Q$ factors and resonance intensity of MM loaded with MXene under different values of the asymmetry parameter $\alpha$.

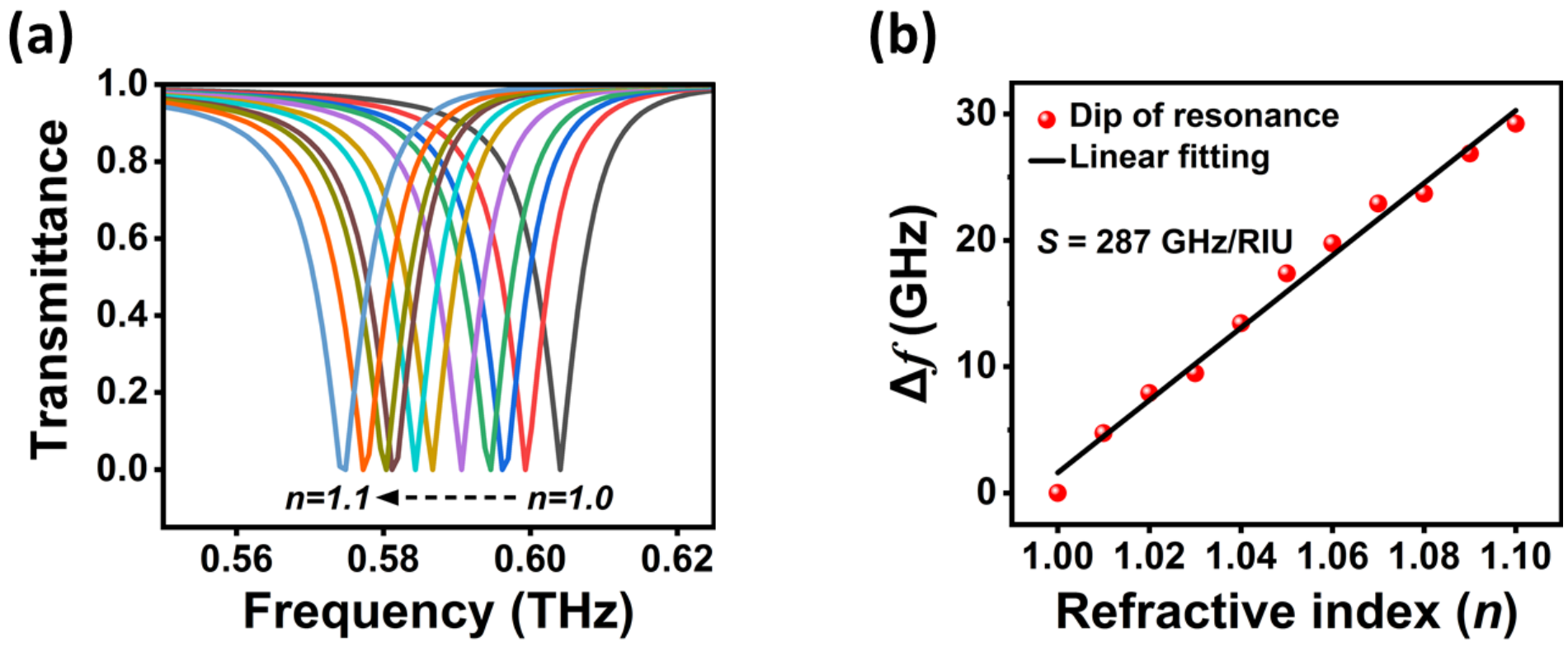


Figure S10. (a) Simulations of transmission spectra of the QBIC MM coated with equivalent dielectric films of different refractive indices with refractive indices varying from 1.0 to 1.1. The black dashed arrow denotes the frequency shift as the refractive index increases. (b) Dip-to-dip resonance frequency shift versus the change in the refractive indices of the analyte. The sensitivity (*S*) of the metasensor reached 287 GHz/RIU.

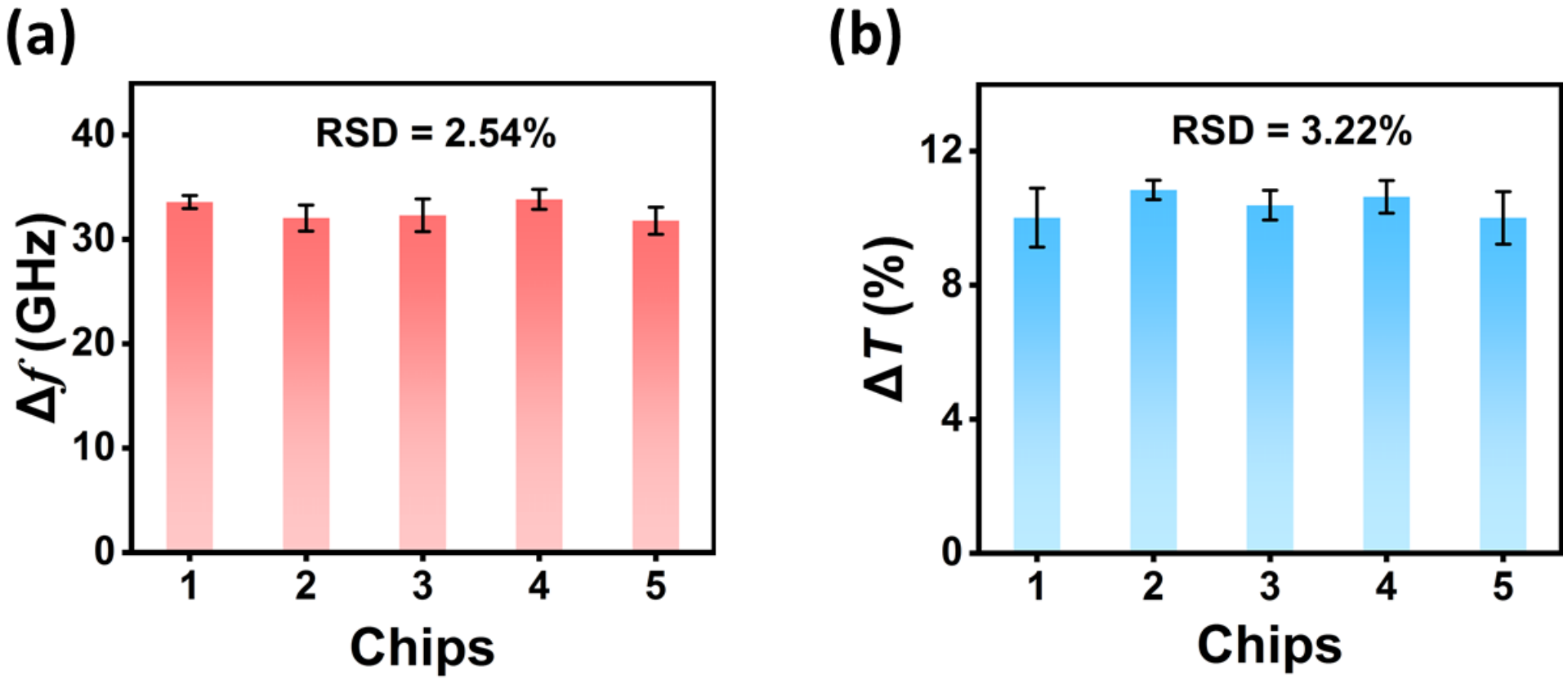


Figure S11. (a) $\Delta f$ responses and (b) $\Delta T$ responses of different chips for the detection of MXene. Data are presented as the mean ± SD of n = 3 replicates.

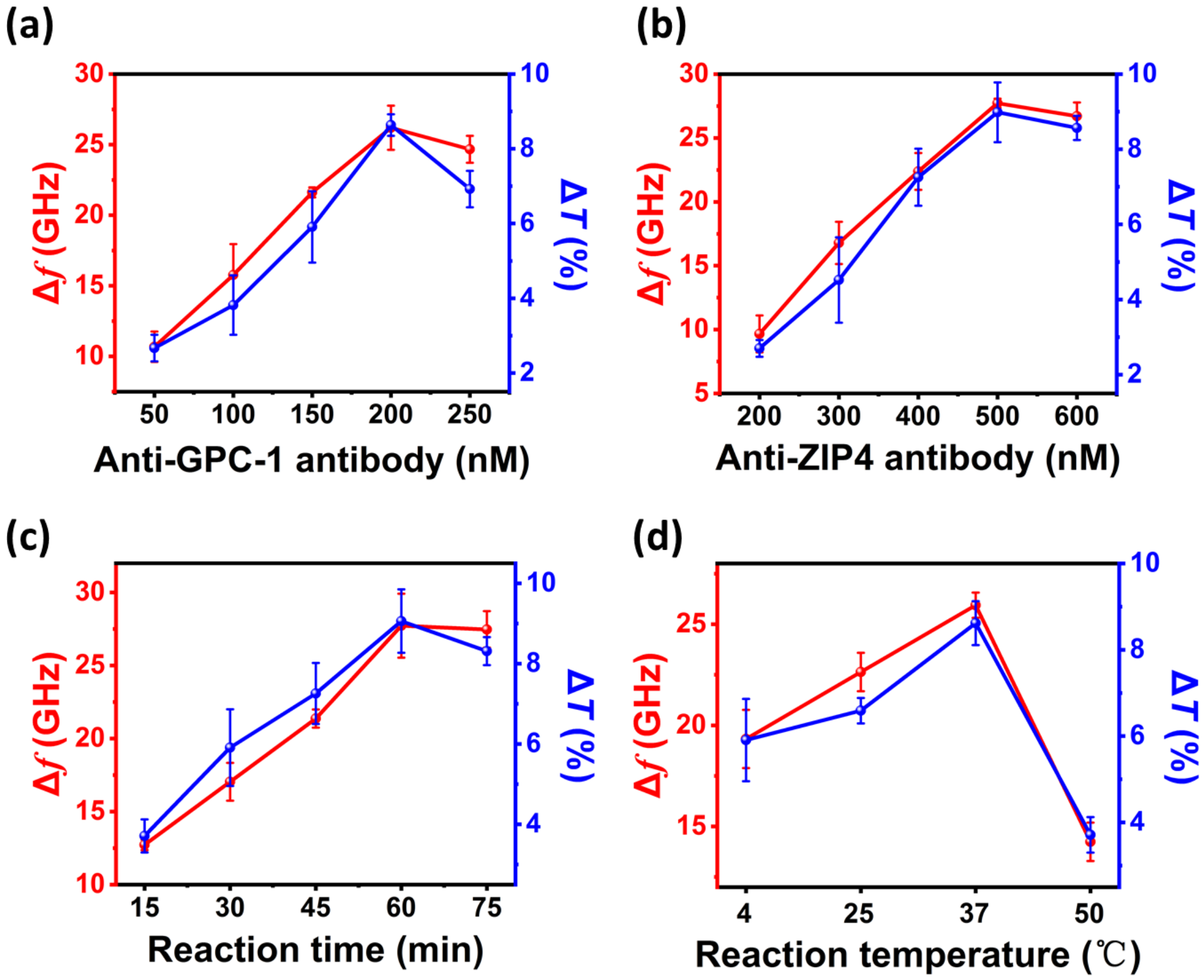


Figure S12. Optimization of assay parameters on the basis of $\Delta f$ and $\Delta T$ responses. The (a) anti-GPC-1 antibody concentration, (b) anti-ZIP4 antibody concentration, (c) coincubation time, and (d) coincubation temperature were optimized using $\Delta f$ and $\Delta T$ as readouts. Data are presented as the mean ± SD of n = 3 replicates.

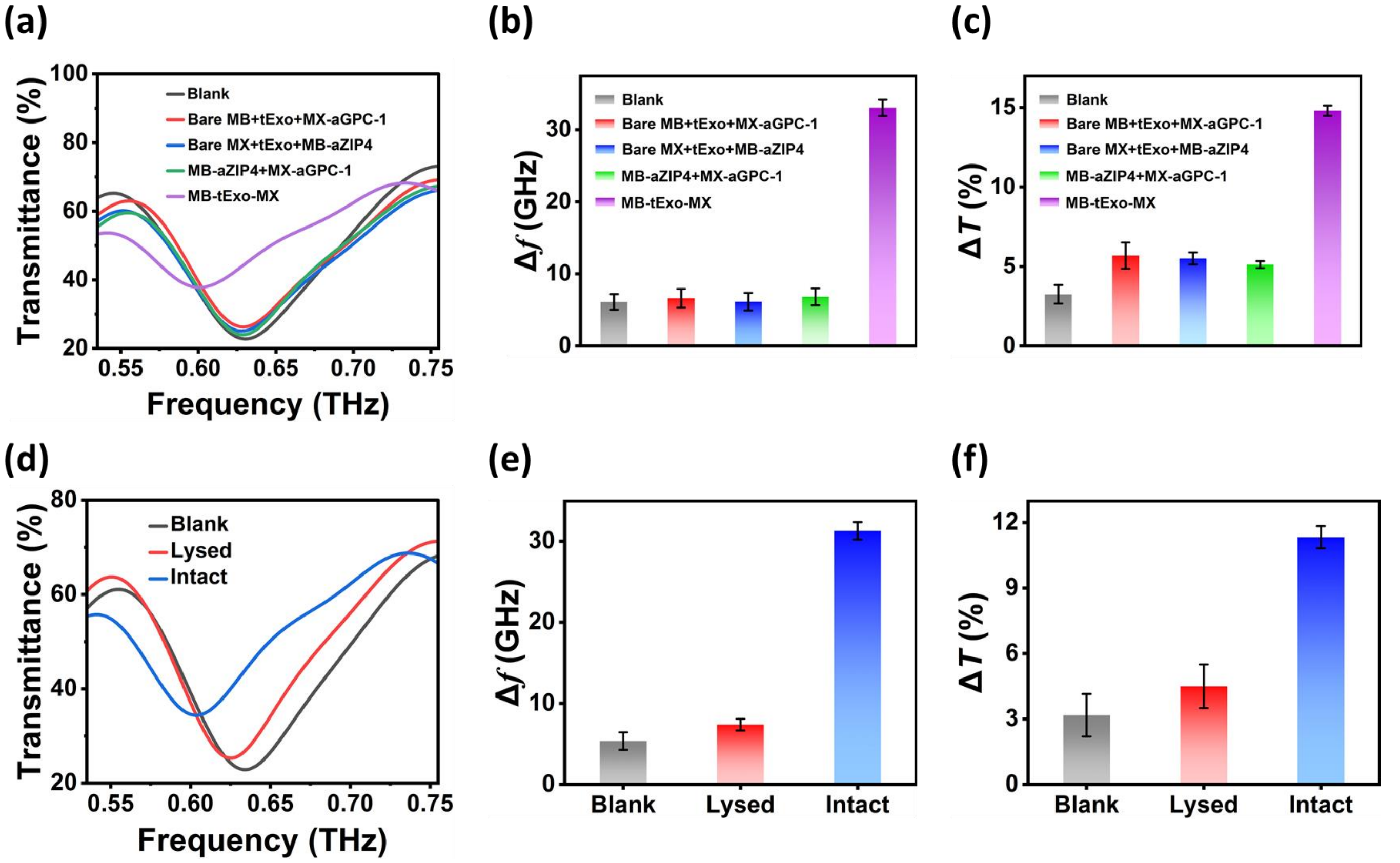


Figure S13. (a) THz transmission spectra of the blank control, nonspecific adsorption controls (bare MBs with tExo and MX-aGPC-1, bare MXene with tExo and MB-aZIP4, and MB-aZIP4 mixed with MX-aGPC-1 without tExo), and the specific capture complex (MB-tExo-MX). (b, c) Corresponding $\Delta f$ and $\Delta T$ for the groups shown in (a). (d) THz transmission spectra of the blank control, Triton X-100-lysed tExos, and intact tExos after probe incubation. (e, f) Corresponding $\Delta f$ and $\Delta T$ for the groups shown in (d). All the quantitative data are presented as the mean $\pm$SD of n = 3 replicates.

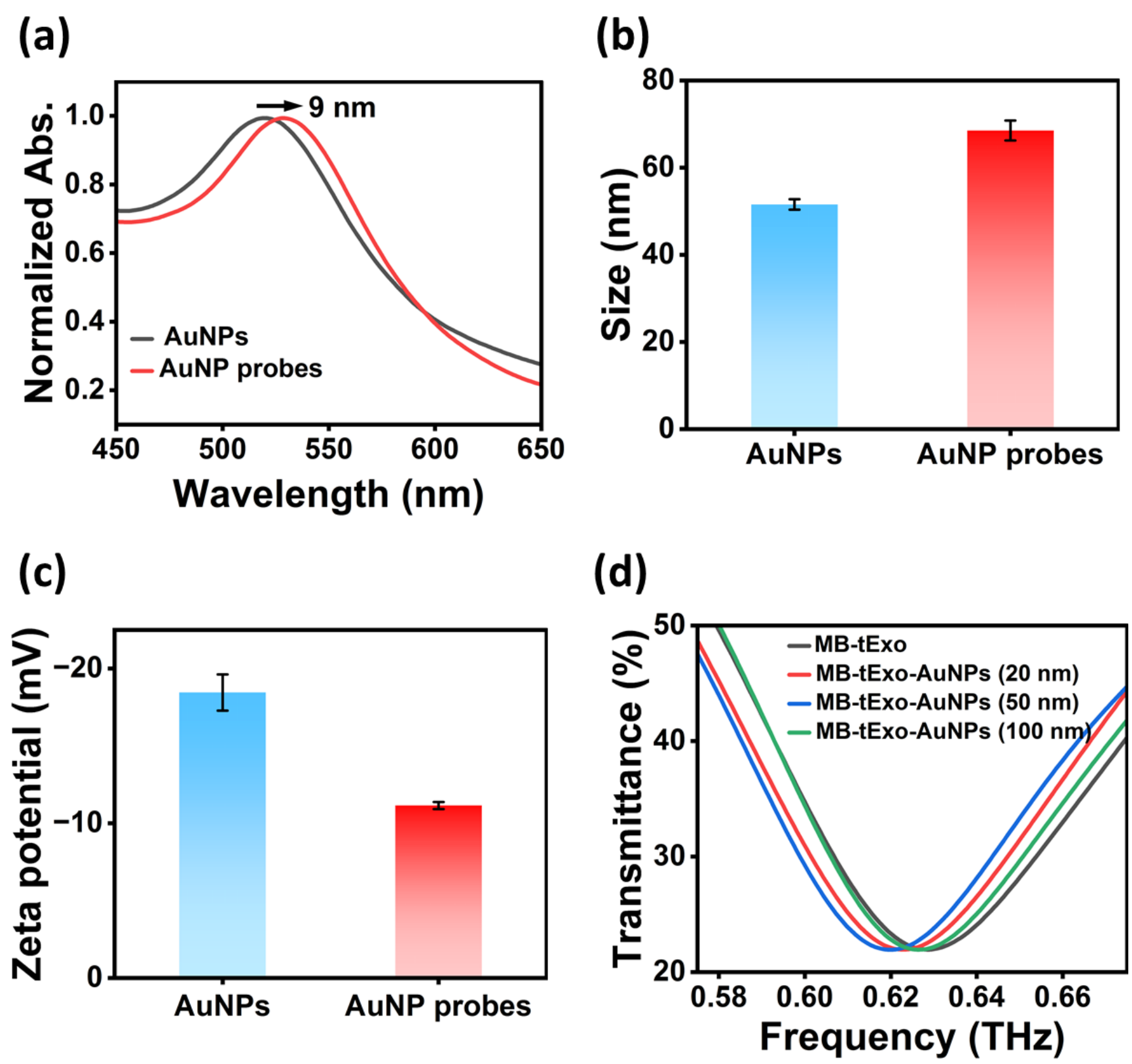


Figure S14. Characterization and size optimization of AuNP-aGPC-1 probes. (a–c) UV–vis absorption spectra, hydrodynamic diameters and zeta potentials of AuNPs and the corresponding AuNP-aGPC-1 probes. After binding of the antibody to the AuNPs, a 9 nm redshift of the absorption peak was observed in the UV–vis absorption spectra, as well as increases in particle size and a shift of the zeta potential toward less negative values, demonstrating the successful conjugation of the AuNP probes. (d) THz signal amplification performance of AuNP-aGPC-1 probes prepared using 20-, 50-, and 100-nm AuNPs, among which the 50-nm AuNP-aGPC-1 probes produced the strongest THz response. All quantitative data are presented as the mean ± SD of n = 3 replicates.

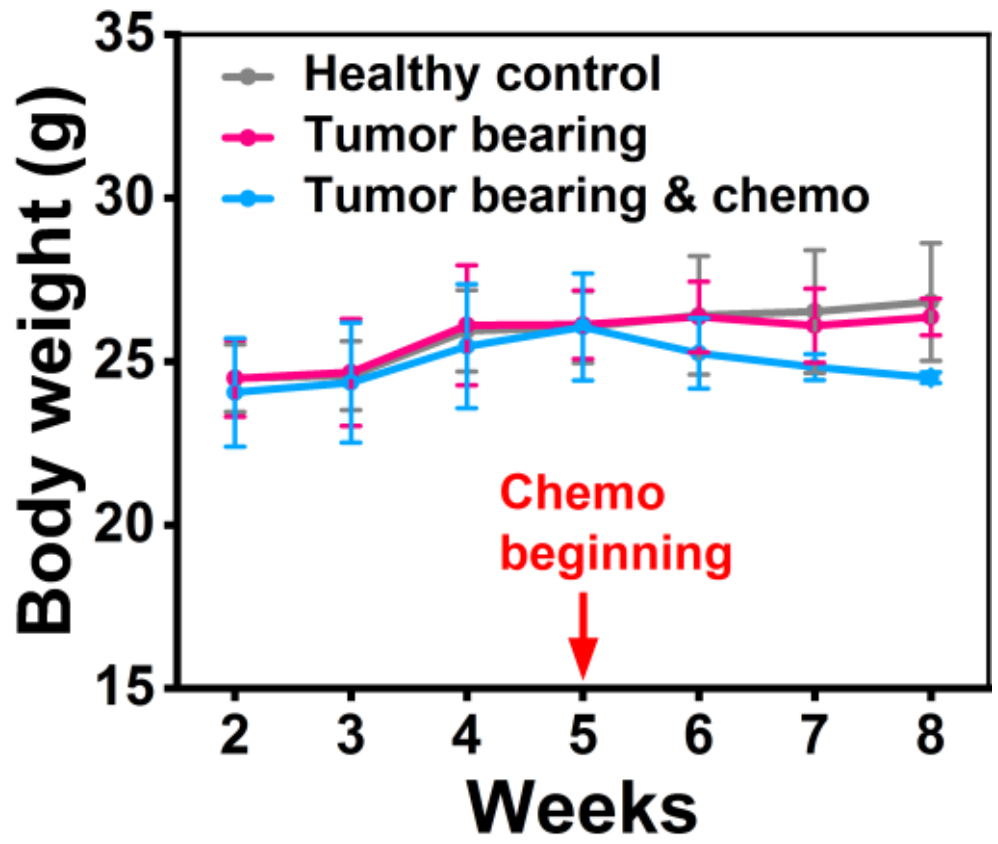


Figure S15. Changes in body weight of mice in the healthy control, tumor bearing and tumor bearing & chemo groups from 2 to 8 weeks after inoculation. The red arrow indicates the initiation time of chemotherapy intervention. Data are presented as the mean ± SD of n = 10 mice.

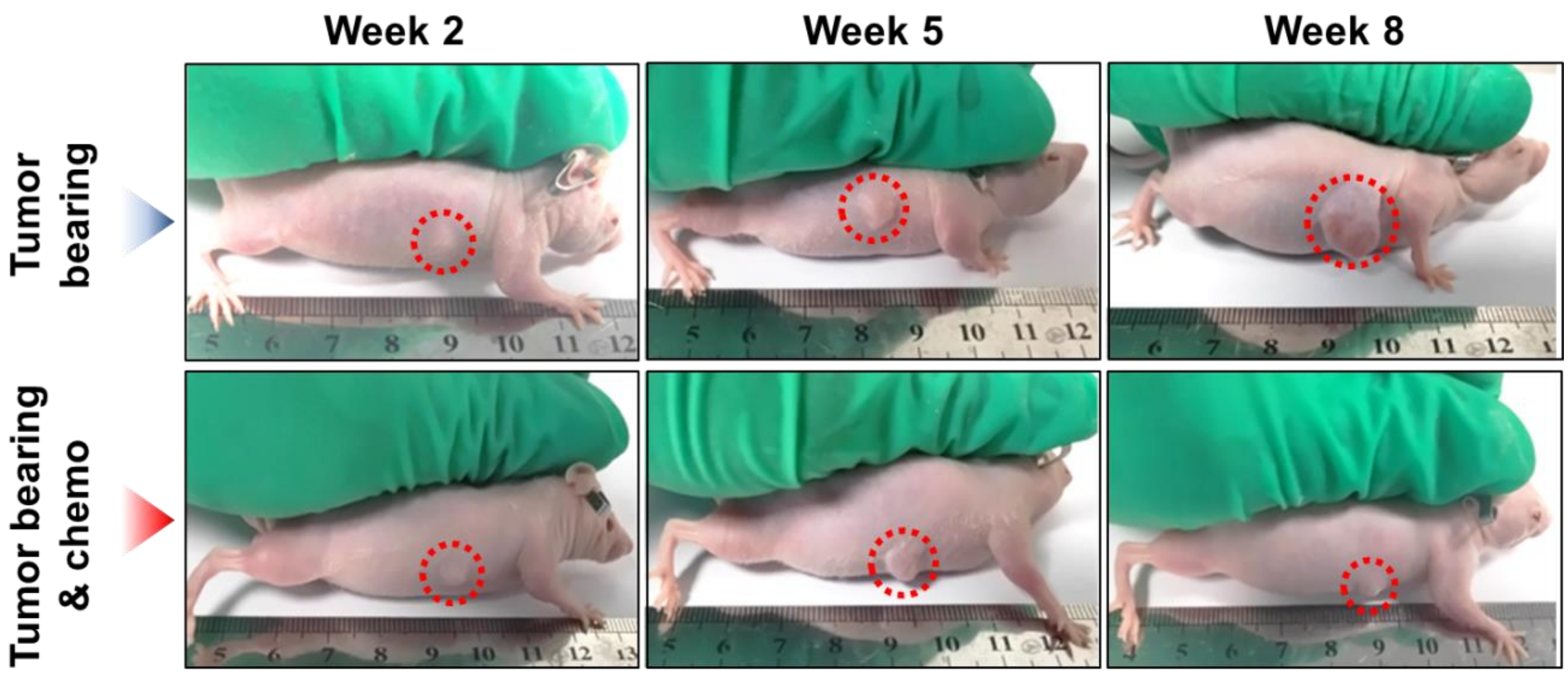


Figure S16. Representative images of mice in the tumor bearing group (top) and the tumor bearing & chemo group (bottom) at 2, 5, and 8 weeks after tumor inoculation. Red dashed circles indicate the tumors.

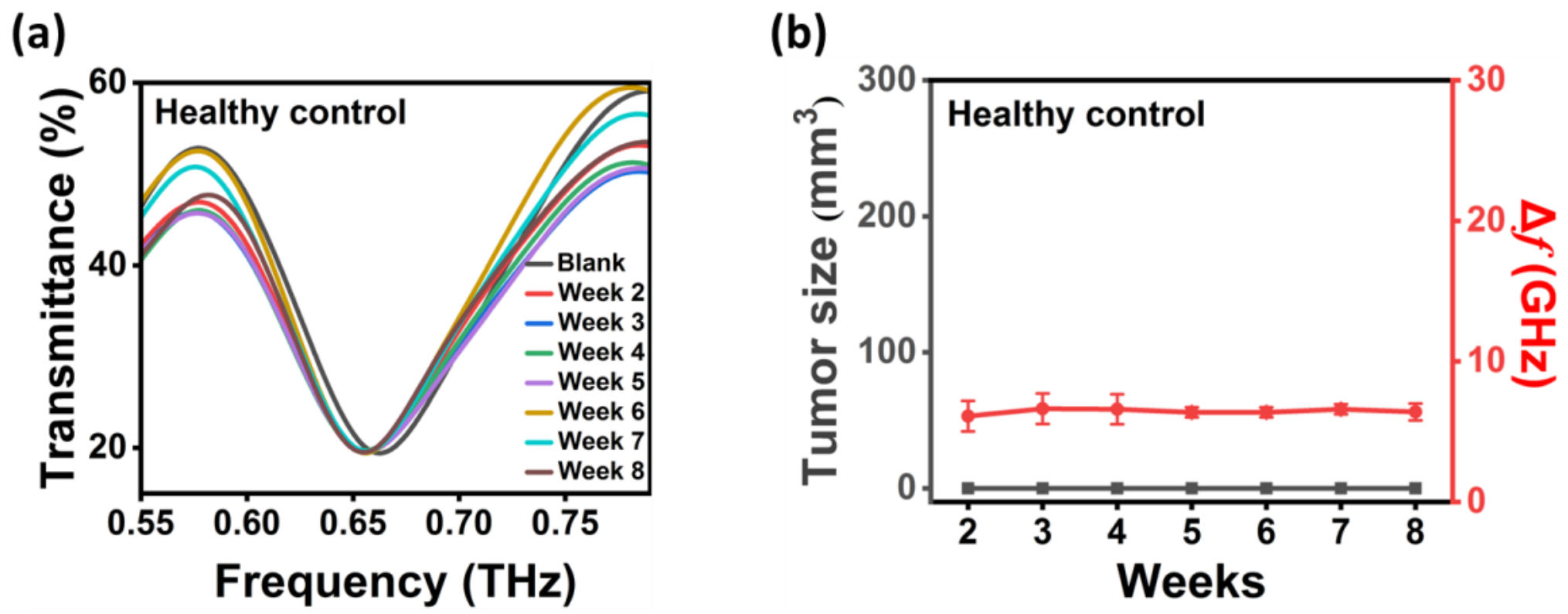


Figure S17. (a) THz transmission spectra of healthy control mice at different time points. (b) $\Delta f$ of healthy control mice at different time points. Data are presented as the mean ± SD of n = 10 mice.

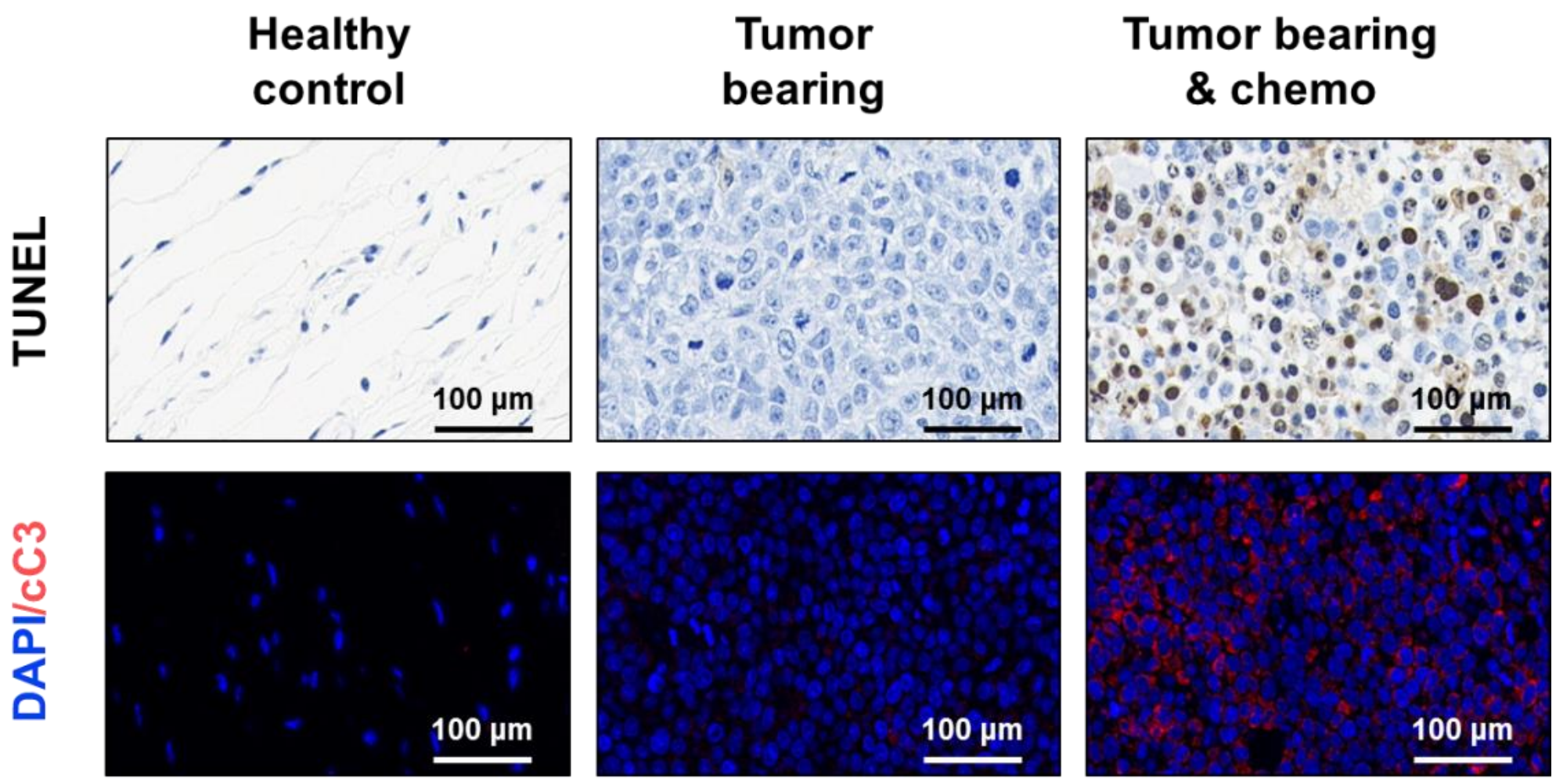


Figure S18. Representative TUNEL staining for apoptotic cells (brown; top) and immunofluorescence staining for cleaved caspase-3 (cC3, red; bottom) in normal subcutaneous tissue from healthy control mice and tumor tissue from untreated or chemotherapy-treated tumor-bearing mice. Nuclei were counterstained with DAPI (blue). Scale bars: 100 μm.

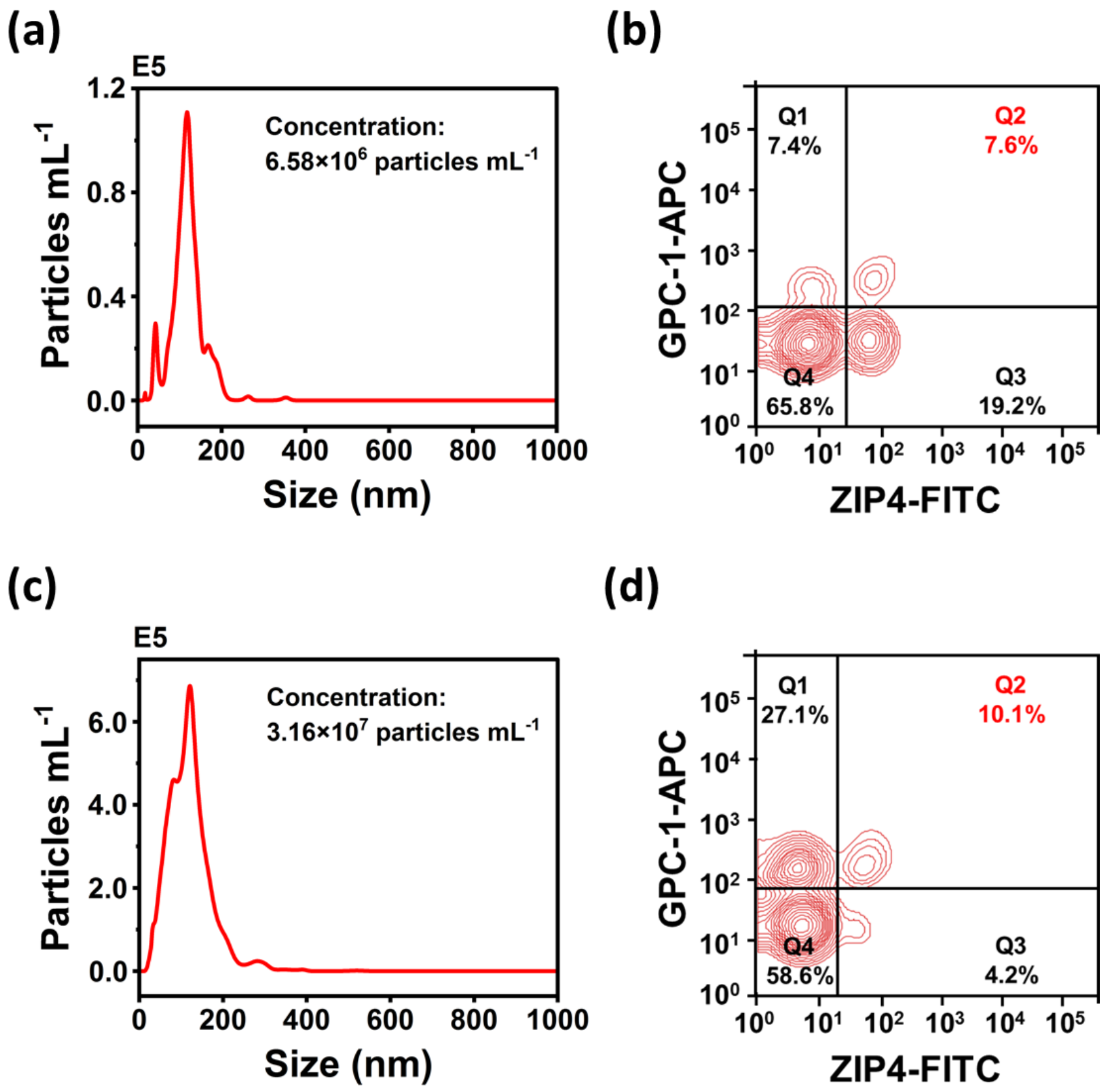


Figure S19. Quantification of exosomes isolated from the serum of tumor bearing & chemo mice before and after chemotherapy. (a) NTA of serum-derived exosomes at week 5, immediately before chemotherapy initiation. (b) Nano-flow cytometry analysis of the corresponding exosome sample following GPC-1-APC and ZIP4-FITC staining. (c) NTA of serum-derived exosomes at week 6, one week after chemotherapy initiation. (d) Nano-flow cytometry analysis of the corresponding exosome sample. The Q2 population represents GPC-$1^+$/ZIP$4^+$ double-positive exosomes.

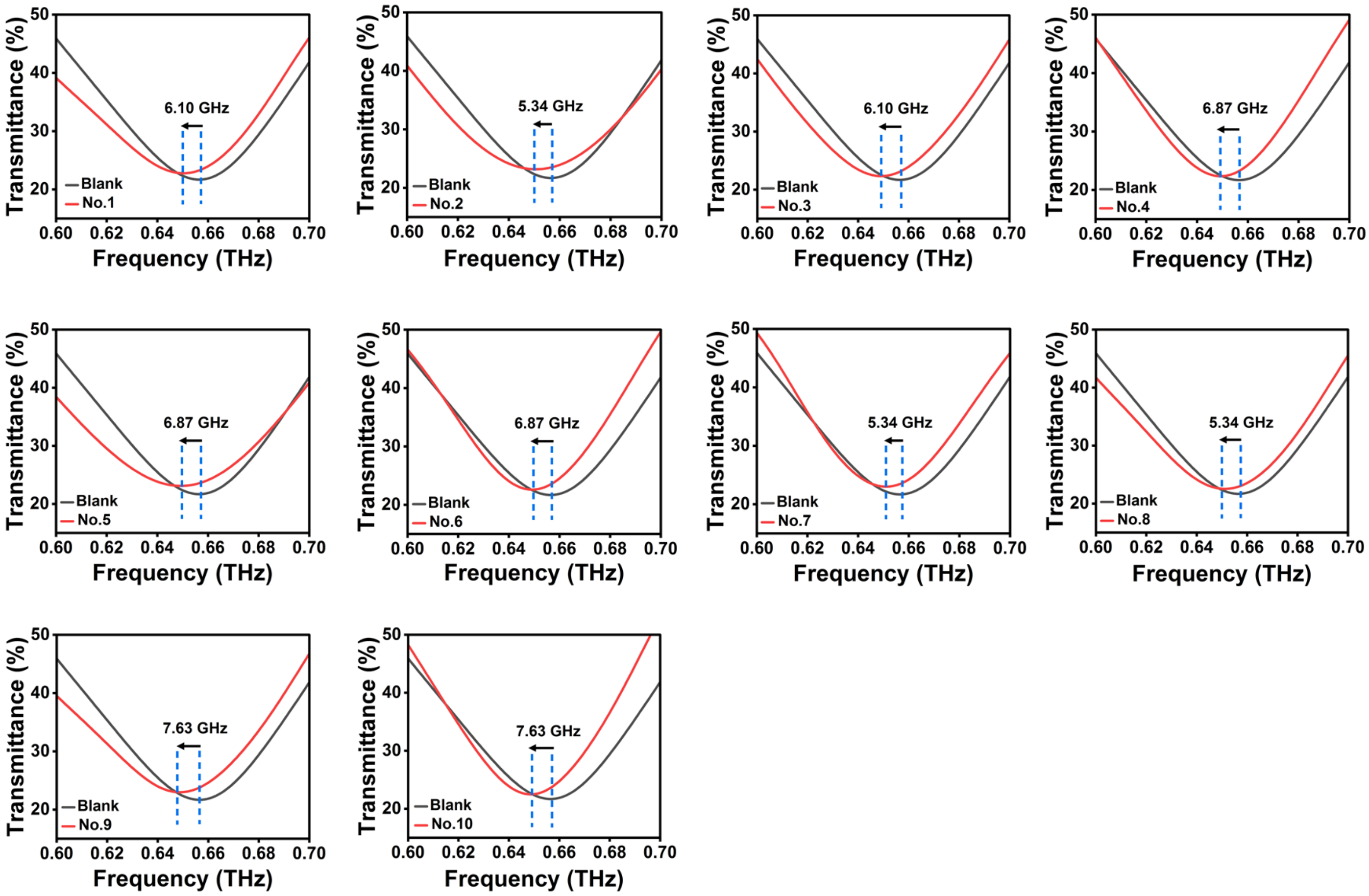


Figure S20. THz transmission spectra and frequency shift changes in serum samples from 10 mice in the healthy control group.

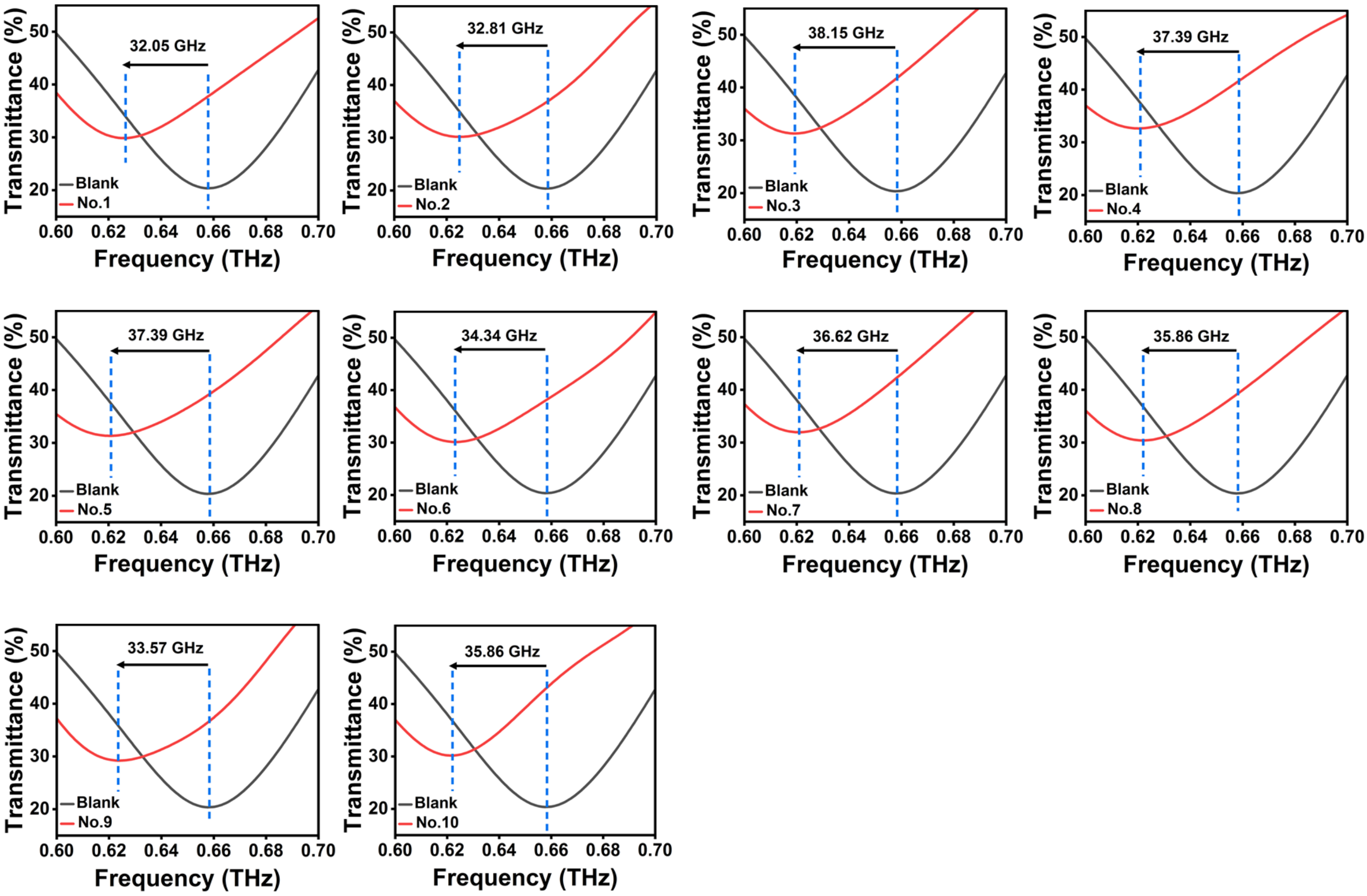


Figure S21. THz transmission spectra and frequency shift changes in serum samples from 10 mice in the tumor bearing group.

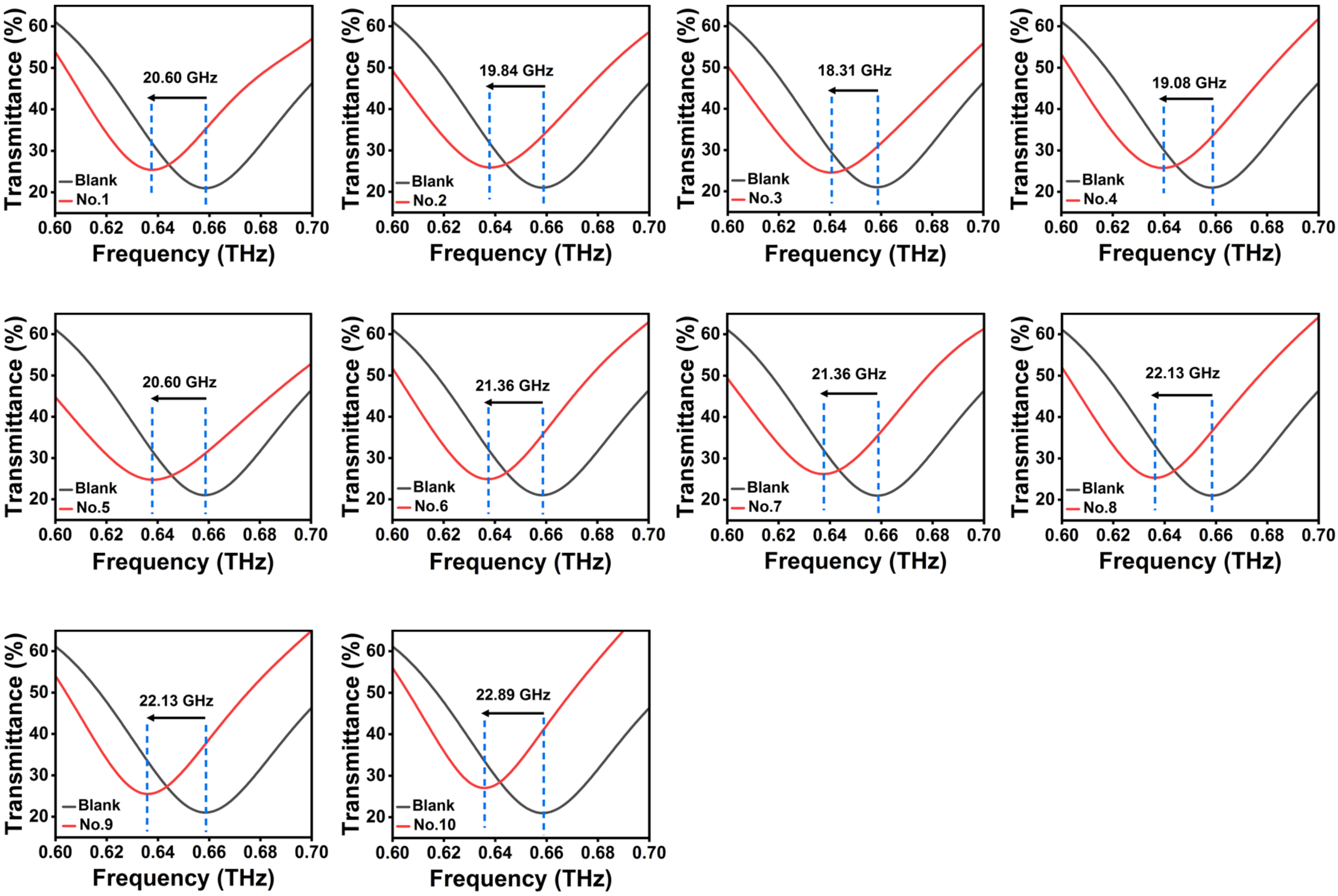


Figure S22. THz transmission spectra and frequency shift changes in serum samples from 10 mice in the tumor bearing & chemo group.

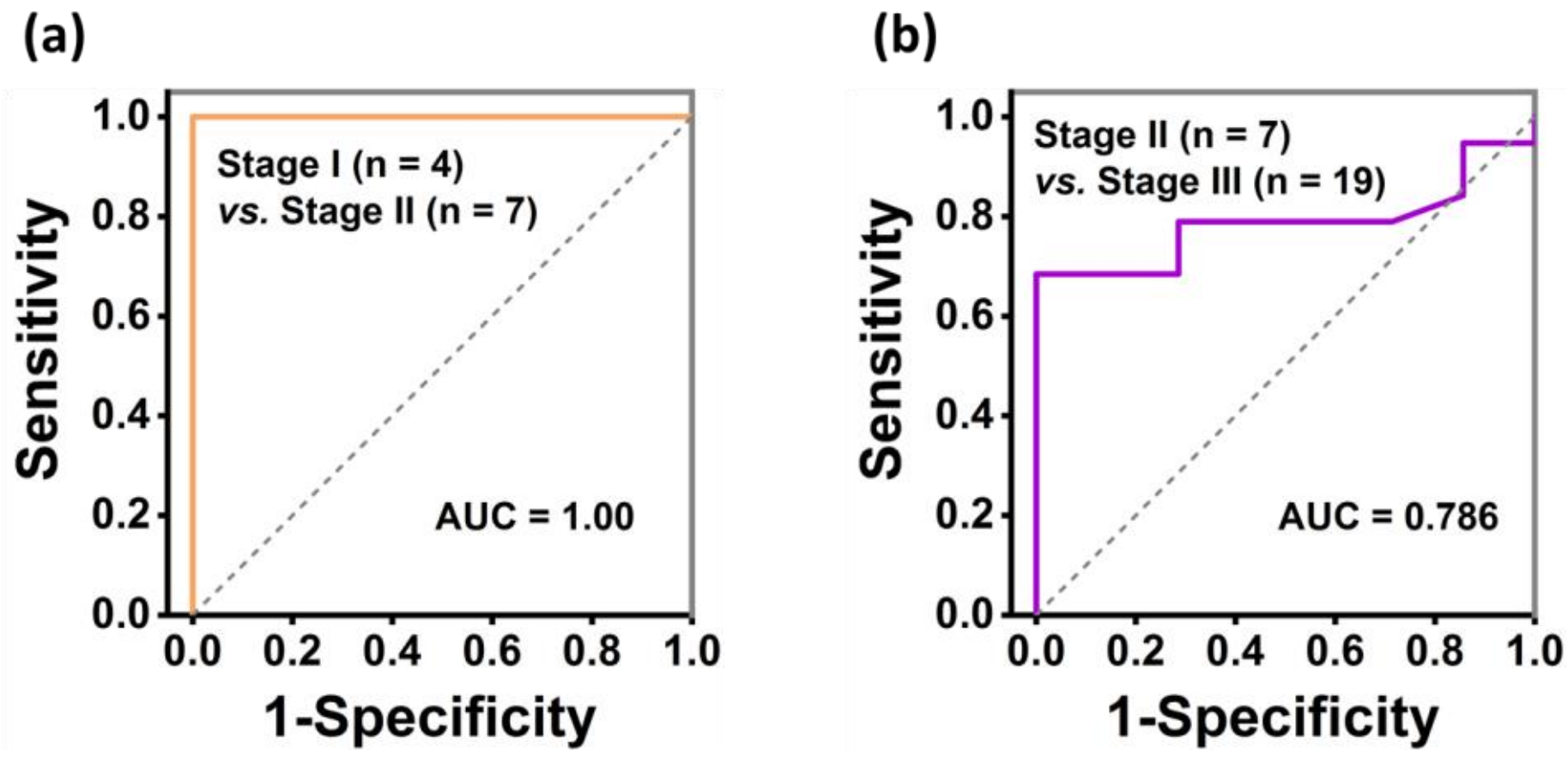


Figure S23. Pairwise ROC analyses of $\Delta f$ for adjacent-stage classification in patients with PC. (a) ROC curve for discriminating Stage I (n = 4) from Stage II (n = 7) disease. (b) ROC curve for discriminating Stage II (n = 7) from Stage III (n = 19) disease.

**Table S1. Performance comparison of various THz metasensors.**

| Resonance type | *Q* factor | Sensitivity | Frequency (THz) | Target | Ref. |
|---|---|---|---|---|---|
| Toroidal moment-based metamaterial | 9.6 (Sim.) | 27.3 GHz/RIU | 0.402 | / | [2] |
| Fano resonance | 11.674 (Sim.) | 700 GHz/RIU | 2.113 | Hemoglobin | [3] |
| Hybrid plasmonic resonance | 3.6–3.747 (Sim.) | 1,000 GHz/RIU | 0.27-0.281 | / | [4] |
| Metamaterial absorber | 121.4 (Sim.) | 37 GHz/RIU | 1.103 | / | [5] |
| Double-chain resonant cavity QBIC | 70 (Sim.) | 544 GHz/RIU | 2.1 | Lithium citrate, BSA | [6] |
| EIT effect | 63 (Sim.) | 96.2 GHz/RIU | 1.29 | Hemoglobin | [7] |
| Spoof surface plasmon polaritons resonance | 18.339 (Sim.) | 940.5 GHz/RIU | 1.996 | IBV virus | [8] |
| Toroidal resonance | 15 (Sim.) | 288 GHz/RIU | 1.76 | CEA | [9] |
| Localized surface plasmon resonance | 47.2 (Sim.) | 300 GHz/RIU | 0.708 | Isoquercitrin | [10] |
| Toroidal resonance | 45.9 (Sim.) | 403.7 GHz/RIU | 1.95 | Cancer cells | [11] |
| LC resonance | 120.69 (Exp.) | 1,600 GHz/RIU | 0.70 | BSA | [12] |
| EIT metamaterial | 6.8 (Sim.) | 331.3 GHz/RIU | 1.29 | / | [13] |
| Dual-band electric dipole resonance | 25.6 (Sim.) | 98.3 GHz/RIU | 1.76 | / | [14] |
| Fano resonance | 2.76 (Exp.) | 160 GHz/RIU | 1.269 | Water-methanol mixtures | [15] |
| Toroidal resonance | 8.78 (Exp.) | 124 GHz/RIU | 0.55 | Water-methanol mixtures | [16] |
| EIT resonance | 5.8 (Exp.) | 312 GHz/RIU | 1.132 | Fluids | [17] |
| Fano resonance-BIC | 58 (Exp.) | 420 GHz/RIU | 2.065 | Hcy | [18] |
| QBIC resonance | 50 (Exp.) | 674 GHz/RIU | 2.16 | CRP, SAA | [19] |
| ELC resonance | 4.3 (Exp.) | 74 GHz/RIU | 0.38 | Glioma cells | [20] |
| QBIC based all-dielectric resonators | 67.2 (Exp.) | 151 GHz/RIU | 0.671 | Amino acid | [21] |
| Dipole resonance | 3.5 (Exp.) | 447 GHz/RIU | 0.532 | L-proline | [22] |
| EIT resonance | 10 (Sim.), 8.5 (Exp.) | 131.05 GHz/RIU | 1.02 | Cancer cells | [23] |
| LSP resonance | 32 (Sim.) | 66 GHz/RIU | 1.55 | / | [24] |
| Guided mode resonance | 10 (Sim.) | 61 GHz/RIU | 0.60 | / | [25] |
| TD resonance-BIC | > 100 (Sim.), 16 (Exp.) | 165 GHz/RIU | 1.17 | IL-6 | [26] |
| Fano resonance-BIC | > 75 (Sim.), 6.45 (Exp.) | 24.5 GHz/RIU | 0.66 | / | [27] |
| QBIC resonance | 82 (Sim.), 7 (Exp.) | 287 GHz/RIU | 0.60 | tExos | **This work** |

**Table S2. Comparison of the performance of our THz metasensor with other biosensors for exosome detection.**

| Methodology | Disease | Recognition sites | Recognition element | Signal element | Linear range (particles $mL^{-1}$) | LOD (particles $mL^{-1}$) | Ref. |
|---|---|---|---|---|---|---|---|
| Electrochemiluminescence | / | CD63 | Aptamer | luminol/AuNPs–$Ti_3C_2$ MXene | $10^5$—$10^8$ | $3 \times 10^4$ | [28] |
| Electrochemistry | Prostate cancer | PSMA | Peptide | AgNPs | $10^5$—$10^{11}$ | $3.7 \times 10^4$ | [29] |
| Fluorescence | / | CD63 | Aptamer | QDs | $5 \times 10^5$—$1 \times 10^8$ | $5 \times 10^5$ | [30] |
| Electrochemistry | / | CD63 | Aptamer | MB | $10^6$—$10^9$ | $1 \times 10^6$ | [31] |
| Electrochemistry | Breast cancer | CD63, HER2 | Aptamer | MB, DSPE-PEG | $10^3$—$10^9$ | $8.11 \times 10^2$ | [32] |
| Electrochemistry | Gastric cancer | CD63, MUC1 | Antibody and aptamer | DNAzyme-$H_2O_2$ | $4.8 \times 10^3$—$4.8 \times 10^6$ | $9.54 \times 10^2$ | [33] |
| Electrochemistry | / | CD63 | Aptamer and cholesterol probe | MB modified hairpin DNA | $5 \times 10^4$—$1 \times 10^8$ | $1.6 \times 10^4$ | [34] |
| Electrochemistry | Ovarian cancer | MUC1 | Aptamer | MB modified DNA analogs | $7.9 \times 10^3$—$3.15 \times 10^8$ | $3 \times 10^4$ | [35] |
| Chemiluminescence | Lung adenocarcinoma | CD63, PD-L1 | Antibody | NiCo-DH-AA chemiluminescence probe | $4.75 \times 10^3$—$4.75 \times 10^8$ | $7.76 \times 10^2$ | [36] |
| Fluorescence | / | CD63 | Aptamer | FAM-labeled ssDNA | $10^5$—$10^{10}$ | $4.21 \times 10^4$ | [37] |
| Fluorescence | / | CD63 | Aptamer and cholesterol probe | Cy3 | $1.1 \times 10^7$—$1.1 \times 10^{12}$ | $2.5 \times 10^6$ | [38] |
| Fluorescence | / | CD63 | Aptamer | FAM | $2 \times 10^4$—$2 \times 10^9$ | $8.2 \times 10^3$ | [39] |
| Electrochemistry | Non-small cell lung cancer | EpCAM | Aptamer | MB | $3.45 \times 10^2$—$3.45 \times 10^7$ | $2.34 \times 10^2$ | [40] |
| SERS | Thyroid cancer | / | / | MXene-coated Au@AgNP | $0.5 \times 10^{10}$—$2 \times 10^{11}$ | $1.7 \times 10^9$ | [41] |
| SERS | Breast cancer | CD9, HER2 | Antibody | MBA | $1.92 \times 10^5$—$1.2 \times 10^8$ | $4.1 \times 10^4$ | [42] |
| Colorimetry | Breast cancer | CD9, HER2 | Antibody | Au@PtOs-$H_2O_2$ | $4.8 \times 10^6$—$6 \times 10^8$ | $2.6 \times 10^6$ | [42] |
| Photothermal method | Breast cancer | CD9, HER2 | Antibody | Au@PtOs | $9.6 \times 10^5$—$1.2 \times 10^8$ | $4.6 \times 10^5$ | [42] |
| Fluorescence | / | CD63 | Aptamer | Carbon dots | $2.5 \times 10^2$—$5.0 \times 10^8$ | $1.0 \times 10^2$ | [43] |
| SPR | Pancreatic cancer | EpCAM, GPC1, HER2 | Aptamer | / | $10^7$—$10^{10}$ | $4.68 \times 10^6$ | [44] |
| Electrochemistry | Non-small cell lung cancer | PD-L1 | Aptamer | MB | $1.2 \times 10^5$—$1.2 \times 10^{10}$ | $3.8 \times 10^4$ | [45] |
| THz | Pancreatic cancer | GPC-1, ZIP4 | Antibody | MXene | $10^3$—$10^9$ | 647 | **This work** |

**Table S3. Mechanistic comparison of THz spectroscopy with representative detection principles.**

| Attribute | THz spectroscopy | Fluorescence | Electrochemistry | SERS | SPR |
|---|---|---|---|---|---|
| **Signal generation mechanism** | Low-frequency absorption/dispersion from intermolecular/collective modes, dielectric relaxation, lattice phonons, rotations, and carrier response | Electronic absorption followed by spontaneous photon emission from an excited state | Interfacial electron/ion transfer, double-layer charging, or impedance/potential change | Plasmon-enhanced inelastic Raman scattering from molecular normal vibrational modes | Refractive-index-dependent shift of surface-plasmon resonance condition |
| **Requires chemical signal-generating label** | No (spectroscopy is label-free) | Usually yes for extrinsic fluorescence assays | Not necessarily; redox reporters are common, but EIS/capacitive /direct redox can be label-free | No (label-free SERS is possible; Raman tags are optional) | No (label-free refractive-index sensing) |
| **Photon energy/primary damage risk** | ≈ 0.4–41 meV for 0.1–10 THz; non-ionizing | Visible/UV excitation may photobleach fluorophores or photodamage samples | Faradaic reactions/electrolysis can alter analyte; non-Faradaic impedance can be milder | Laser/plasmonic heating may damage analyte at high power or resonance | Generally minimally invasive; photothermal heating possible under resonant illumination |
| **Biomolecular mode specificity/matching** | Yes for low-frequency intermolecular/collective modes | No (fluorescence matches electronic transitions, not low-frequency vibrations) | No optical mode matching; reports interfacial charge/ion processes | Measures vibrational Raman shifts (normal modes); excitation is usually non-resonant unless resonance Raman/SERRS | No intrinsic molecular vibrational specificity; mainly surface refractive-index sensing |

**Table S4. Systematic comparison of the proposed THz metasensor with state-of-the-art and mainstream exosome detection technologies.**

| Technology | Representative platform | Transduction principle | LOD | Assay time | Key strengths | Key limitations | Ref. |
|---|---|---|---|---|---|---|---|
| Digital single-molecule counting immunoassay | Simoa (Quanterix) | Enzyme-catalyzed fluorescence in femtoliter wells | $2.5\times10^{4}$–$3.4\times10^{4}$ particles $mL^{-1}$ | ~2 h | Full automation | Multistep washing; dedicated high-cost analyzer | [46] |
| Homogeneous chemiluminescence assay | ExoScreen | AlphaLISA singlet-oxygen transfer | / | 1.5–3 h | No washing; direct serum detection | Optical labels; particle-level LOD not established | [47] |
| Nanoplasmonic sensing | nPLEX | Transmission SPR through nanohole arrays | ~3,000 exosomes (absolute count) | < 30 min | Label-free; multiplexed profiling | Complex nanofabrication | [48] |
| Nano-flow cytometry | nanoFCM | Light scattering/fluorescence of single particles | $2.5\times10^{7}$ particles $mL^{-1}$ | ~50 min | Single-particle counting | Requires purified EVs; matrix interference | [49] |
| Conventional ELISA | Commercial kits | Enzyme-catalyzed colorimetry | $10^{6}$–$10^{8}$ particles $mL^{-1}$ | 4–6 h | Standardized | Low sensitivity; large sample; multistep | [50] |
| NTA | Commercial NTA | Laser scattering tracking | $10^{6}$ particles $mL^{-1}$ | ~10 min | Size + concentration | Lacks molecular specificity; vulnerable to matrix contamination | [51] |
| THz | Immunomagnetic-MXene THz metasensor | QBIC THz resonance shift (dielectric transduction) | 647 particles $mL^{-1}$ | 1–1.5 h | Dual-marker specificity; MXene amplification; | Lab-grade THz-TDS; drying step; limited throughput | **This work** |

*** LOD values from comparative technologies are reproduced from the cited literature. The LOD of the proposed THz metasensor was measured in spiked buffer samples; matrix effects from raw clinical serum may degrade practical sensing performance.**

**Table S5. Summary information of the 60-subject cohort.**

| **Subjects** | | **HDs** | **Early** | **Adv.** |
|---|---|---|---|---|
| **Number** | | 30 | 11 | 19 |
| **Age (years)** | | 57.88 ± 12.51 | 62.09 ± 7.79 | 61.16 ± 10.17 |
| **Gender** | Male | 18 | 7 | 10 |
| | Female | 12 | 4 | 9 |
| **Histopathological subtypes** | | / | PDAC | PDAC |
| **T** | T1 | / | 3 | 0 |
| | T2 | | 5 | 9 |
| | T3 | | 3 | 10 |
| | T4 | | 0 | 0 |
| **N** | N0 | / | 5 | 0 |
| | N1 | | 6 | 0 |
| | N2 | | 0 | 19 |
| **M** | M0 | / | 11 | 19 |
| | M1 | | 0 | 0 |

*** HDs, healthy donors; Early, early stage; Adv., advanced stage; PDAC, pancreatic ductal adenocarcinoma; T: describes the size and local extent of the primary tumor; T1: tumor ≤ 2 cm in greatest dimension; T2: tumor > 2 cm and ≤ 4 cm in greatest dimension; T3: tumor > 4 cm in greatest dimension; T4: tumor involving the celiac axis, superior mesenteric artery, or common hepatic artery. N: describes regional lymph node involvement; N0: no regional lymph node metastasis; N1: 1–3 regional lymph node metastases; N2: ≥ 4 regional lymph node metastases. M: indicates whether distant metastasis is present; M0: no distant metastasis; M1: with distant metastasis. Tumor staging was performed according to the 8th edition of the AJCC Cancer Staging Manual. Early stage was defined as Stage Ia–IIb [T(1–3), N(0–1), M0], and advanced stage was defined as Stage III [T(1–3), N2, M0, or T4, any N, M0] and Stage IV [any T, any N, M1]; all 19 advanced cases in this cohort were T(2–3), N2, M0 without distant metastasis. No significant differences in age or gender were observed between the healthy donor and PC patient groups ($P$ > 0.05).**

**Table S6. Δ*f* values in healthy donors and PC patients.**

| HD No. | Δ*f* (GHz) | Patient No. | Stage | Δ*f* (GHz) |
|---|---|---|---|---|
| 1 | 6.10 | 1 | I | 19.08 |
| 2 | 6.36 | 2 | II | 29.25 |
| 3 | 10.68 | 3 | I | 18.31 |
| 4 | 7.38 | 4 | II | 28.23 |
| 5 | 6.36 | 5 | II | 30.52 |
| 6 | 6.87 | 6 | II | 27.47 |
| 7 | 6.61 | 7 | II | 21.87 |
| 8 | 7.38 | 8 | II | 27.72 |
| 9 | 7.12 | 9 | II | 30.52 |
| 10 | 6.36 | 10 | I | 14.50 |
| 11 | 14.50 | 11 | I | 18.06 |
| 12 | 6.87 | 12 | III | 33.57 |
| 13 | 6.61 | 13 | III | 35.86 |
| 14 | 9.16 | 14 | III | 35.10 |
| 15 | 9.41 | 15 | III | 32.81 |
| 16 | 8.90 | 16 | III | 33.83 |
| 17 | 8.65 | 17 | III | 29.76 |
| 18 | 11.70 | 18 | III | 27.47 |
| 19 | 8.39 | 19 | III | 31.28 |
| 20 | 8.65 | 20 | III | 32.05 |
| 21 | 7.63 | 21 | III | 32.81 |
| 22 | 12.21 | 22 | III | 22.89 |
| 23 | 7.88 | 23 | III | 34.08 |
| 24 | 8.14 | 24 | III | 18.82 |
| 25 | 18.31 | 25 | III | 31.79 |
| 26 | 6.87 | 26 | III | 24.42 |
| 27 | 8.39 | 27 | III | 35.10 |
| 28 | 7.88 | 28 | III | 31.54 |
| 29 | 7.12 | 29 | III | 34.59 |
| 30 | 8.90 | 30 | III | 29.76 |

**Table S7. CA19-9 levels in healthy donors and PC patients.**

| HD No. | CA19-9 (U/mL) | Patient No. | Stage | CA19-9 (U/mL) |
|---|---|---|---|---|
| 1 | 30.55 | 1 | I | 30.45 |
| 2 | 26.75 | 2 | II | 35.19 |
| 3 | 29.25 | 3 | I | 25.81 |
| 4 | 30.76 | 4 | II | 44.17 |
| 5 | 15.08 | 5 | II | 20.75 |
| 6 | 26.38 | 6 | II | 39.79 |
| 7 | 21.58 | 7 | II | 37.62 |
| 8 | 12.54 | 8 | II | 48.38 |
| 9 | 11.53 | 9 | II | 71.67 |
| 10 | 38.67 | 10 | I | 45.73 |
| 11 | 14.23 | 11 | I | 41.19 |
| 12 | 26.39 | 12 | III | 120.97 |
| 13 | 11.35 | 13 | III | 122.32 |
| 14 | 28.97 | 14 | III | 165.72 |
| 15 | 20.23 | 15 | III | 115.89 |
| 16 | 69.80 | 16 | III | 102.92 |
| 17 | 16.46 | 17 | III | 148.53 |
| 18 | 20.87 | 18 | III | 136.64 |
| 19 | 22.77 | 19 | III | 177.53 |
| 20 | 27.75 | 20 | III | 121.86 |
| 21 | 33.90 | 21 | III | 133.44 |
| 22 | 56.48 | 22 | III | 34.61 |
| 23 | 24.85 | 23 | III | 136.15 |
| 24 | 23.69 | 24 | III | 122.45 |
| 25 | 30.92 | 25 | III | 147.77 |
| 26 | 26.13 | 26 | III | 159.67 |
| 27 | 11.90 | 27 | III | 168.42 |
| 28 | 34.74 | 28 | III | 142.31 |
| 29 | 30.74 | 29 | III | 116.37 |
| 30 | 22.14 | 30 | III | 157.44 |

**Table S8. Advantages of the THz metasensor in the early diagnosis of PC compared with existing biomarker detection methods.**

| Biomarkers | Methods | AUC | Steps | Sample volumes | Costs | Ref. |
|---|---|---|---|---|---|---|
| CEA | ELISA | 0.68* | 6+ | 50 μL | > 10 $/test | [52] |
| miRNAs | qRT–PCR | 0.971 | 6 | 250 μL | 200–300 $/test | [53] |
| ctDNA | Various ctDNA assays | 0.92 | 6+ | > 2,000 μL | > 200 $/test | [54] |
| tExo | THz metasensor | 0.991 | 4 | 10 μL | < 5 $/test | **This work** |

*** PC diagnosis, not restricted to early-stage disease.**

**Table S9. Primary antibodies used for Western blot analysis.**

| Targets | Clone numbers | Host species | Working concentrations | Validations |
|---|---|---|---|---|
| GPC-1 | EPR19285 | Rabbit monoclonal | 0.63 μg/mL | Observed band at ~55 kDa in MDA-MB-231 cell lysate |
| ZIP4 | / | Rabbit polyclonal | 0.90 μg/mL | Observed band at ~68 kDa in Colo320 cell lysate |
| CD9 | EPR23105-121 | Rabbit monoclonal | 0.53 μg/mL | Observed band at ~22 kDa in HCT 116 cell lysate |
| CD63 | EPR5702 | Rabbit monoclonal | 0.39 μg/mL | Observed band at 30–65 kDa in A375 cell lysate |
| CD81 | EPR4244 | Rabbit monoclonal | 0.23 μg/mL | Observed band at ~20 kDa in U-87 MG cell lysate |
| TSG101 | EPR7130(B) | Rabbit monoclonal | 0.23 μg/mL | Observed band at ~47 kDa in K562 cell lysate |
| Calnexin | 6F12BE10 | Mouse monoclonal | 1.00 μg/mL | Observed band at ~80 kDa in THP-1 cell lysate |

## References


1. W. Zheng, H. Ramsden, S. Ippolito, et al., "Photothermal effects control ultrafast charge transport in titanium carbide MXenes," *Nature Communications* 17 (2026): 1201, https://doi.org/10.1038/s41467-026-68831-4.

2. M. Gupta, Y. Srivastava, M. Manjappa, et al., "Sensing with toroidal metamaterial," *Applied Physics Letters* 110 (2017): 121108, https://doi.org/10.1063/1.4978672.

3. J. Wekalao, N. Mandela, G. Mwendwa, et al., "Ultra-sensitive Terahertz Hemoglobin Detection Using Graphene-Enhanced Metasurface Surface Plasmon Resonance Biosensors Optimized with K-Nearest Neighbors Regression Machine Learning," *Plasmonics* 21 (2026): 177–192, https://doi.org/10.1007/s11468-025-02816-9.

4. J. Wekalao, H. Elsayed, A. Mehaney, et al., "High-sensitivity terahertz metasurface biosensor for multi-cancer detection: a machine learning-enhanced approach using graphene-MXene-silver-copper hybrid architecture," *Materials Technology* 40 (2025): 2585986, https://doi.org/10.1080/10667857.2025.2585986.

5. D. Wang, K. Xu, S. Luo, et al., "A high Q-factor dual-band terahertz metamaterial absorber and its sensing characteristics," *Nanoscale* 15 (2023): 3398–3407, https://doi.org/10.1039/d2nr05820k.

6. B. Dong, B. Wei, D. Wei, et al., "Detection of Low-Concentration Biological Samples Based on a QBIC Terahertz Metamaterial Sensor," *Sensors* 24 (2024): 3649, https://doi.org/10.3390/s24113649.

7. W. Pan, Y. Yan, Y. Ma, et al., "A terahertz metamaterial based on electromagnetically induced transparency effect and its sensing performance," *Optics Communications* 431 (2019): 115–119, https://doi.org/10.1016/j.optcom.2018.09.014.

8. Z. El-Wasif, T. Ismail, and O. Hamdy, "Design and optimization of highly sensitive multi-band terahertz metamaterial biosensor for coronaviruses detection," *Optical and Quantum Electronics* 55 (2023): 604, https://doi.org/10.1007/s11082-023-04906-6.

9. Q. Niu, L. Fu, Y. Zhong, et al., "Sensitive and Specific Detection of Carcinoembryonic Antigens Using Toroidal Metamaterial Biosensors Integrated with Functionalized Gold Nanoparticles," *Analytical Chemistry* 95 (2023): 1123–1131, https://doi.org/10.1021/acs.analchem.2c03836.

10. J. Wekalao, J. Muheki, H. Elsayed, et al., "AI-Augmented terahertz metamaterial biosensor for rapid and accurate isoquercitrin detection in herbal medicines," *Nanocomposites* 11 (2025): 297–320, https://doi.org/10.1080/20550324.2025.2592170.

11. C. Zhang, T. Xue, J. Zhang, et al., "Terahertz meta-biosensor based on high-Q electrical resonance enhanced by the interference of toroidal dipole," *Biosensors & Bioelectronics* 214 (2022): 114493, https://doi.org/10.1016/j.bios.2022.114493.

12. H. Khand, R. Sengupta, and G. Sarusi, "Novel Octa-Structure Metamaterial Architecture for High Q-Factor and High Sensitivity in THz Impedance Spectroscopy," *Advanced Science* 11 (2024): e2407824, https://doi.org/10.1002/advs.202407824.

13. X. Zhou, L. Li, Y. Shen, et al., "Design, simulation, and experimental realization of a high-sensitivity polarization-independent electromagnetically induced transparent terahertz metamaterials," *Optics Express* 32 (2024): 33130–33143, https://doi.org/10.1364/OE.534499.

14. S. Guo, C. Li, D. Wang, et al., "Dual-band terahertz metamaterial sensor and its sensing capacity enhanced with a central-relief design," *Applied Optics* 63 (2024): 1962–1970, https://doi.org/10.1364/AO.509461.

15. M. Chen, L. Singh, N. Xu, et al., "Terahertz sensing of highly absorptive water-methanol mixtures with multiple resonances in metamaterials," *Optics Express* 25 (2017): 14089–14097, https://doi.org/10.1364/OE.25.014089.

16. J. Xu, D. Liao, M. Gupta, et al., "Terahertz Microfluidic Sensing with Dual-Torus Toroidal Metasurfaces," *Advanced Optical Materials* 9 (2021): 2100024, https://doi.org/10.1002/adom.202100024.

17. T. Lin, Y. Huang, S. Zhong, et al., "Field manipulation of electromagnetically induced transparency analogue in terahertz metamaterials for enhancing liquid sensing," *Optics and Lasers In Engineering* 157 (2022): 107127, https://doi.org/10.1016/j.optlaseng.2022.107127.

18. B. Liu, Y. Peng, Z. Jin, et al., "Terahertz ultrasensitive biosensor based on wide-area and intense light-matter interaction supported by QBIC," *Chemical Engineering Journal* 462 (2023): 142347, https://doi.org/10.1016/j.cej.2023.142347.

19. R. Wang, L. Xu, L. Huang, et al., "Ultrasensitive Terahertz Biodetection Enabled by Quasi-BIC-Based Metasensors,"

*Small* 19 (2023): e2301165, https://doi.org/10.1002/smll.202301165.
20. Q. Wang, K. Li, H. Chi, et al., "Label-free distinguish proliferative and apoptotic responses of glioma cells with terahertz metamaterials," *Sensors and Actuators: B. Chemical* 389 (2023): 133887, https://doi.org/10.1016/j.snb.2023.133887.
21. T. Lin, Y. Huang, S. Zhong, et al., "Passive trapping of biomolecules in hotspots with all-dielectric terahertz metamaterials," *Biosensors & Bioelectronics* 251 (2024): 116126, https://doi.org/10.1016/j.bios.2024.116126.
22. T. Lin, Q. Zeng, Y. Huang, et al., "Substrate-Free Terahertz Metamaterial Sensors With Customizable Configuration and High Performance," *Advanced Optical Materials* 12 (2024): 2400689, https://doi.org/10.1002/adom.202400689.
23. Z. Zhang, M. Yang, X. Yan, et al., "The Antibody-Free Recognition of Cancer Cells Using Plasmonic Biosensor Platforms with the Anisotropic Resonant Metasurfaces," *ACS Applied Materials & Interfaces* 12 (2020): 11388–11396, https://doi.org/10.1021/acsami.0c00095.
24. M. Islam, J. Sultana, M. Biabanifard, et al., "Tunable localized surface plasmon graphene metasurface for multiband superabsorption and terahertz sensing," *Carbon* 158 (2020): 559–567, https://doi.org/10.1016/j.carbon.2019.11.026.
25. M. Gupta and R. Singh, "Terahertz Sensing with Optimized $Q/V_{eff}$ Metasurface Cavities," *Advanced Optical Materials* 8 (2020): 1902025, https://doi.org/10.1002/adom.201902025.
26. R. Wang, L. Xu, J. Wang, et al., "Electric Fano resonance-based terahertz metasensors," *Nanoscale* 13 (2021): 18467–18472, https://doi.org/10.1039/d1nr04477j.
27. T. Tan, Y. Srivastava, R. Ako, et al., "Active Control of Nanodielectric-Induced THz Quasi-BIC in Flexible Metasurfaces: A Platform for Modulation and Sensing," *Advanced Materials* 33 (2021): e2100836, https://doi.org/10.1002/adma.202100836.
28. H. Zhang, Z. Wang, F. Wang, et al., "In Situ Formation of Gold Nanoparticles Decorated Ti3C2 MXenes Nanoprobe for Highly Sensitive Electrogenerated Chemiluminescence Detection of Exosomes and Their Surface Proteins," *Analytical Chemistry* 92 (2020): 5546–5553, https://doi.org/10.1021/acs.analchem.0c00469.
29. W. Cheng, Y. Sun, G. Zhao, et al., "A novel peptide-templated AgNPs nanoprobe for theranostics of prostate cancer," *Biosensors & Bioelectronics* 223 (2023): 114978, https://doi.org/10.1016/j.bios.2022.114978.
30. M. Wu, Z. Chen, Q. Xie, et al., "One-step quantification of salivary exosomes based on combined aptamer recognition and quantum dot signal amplification," *Biosensors & Bioelectronics* 171 (2021): 112733, https://doi.org/10.1016/j.bios.2020.112733.
31. Q. Zhou, A. Rahimian, K. Son, et al., "Development of an aptasensor for electrochemical detection of exosomes," *Methods* 97 (2016): 88–93, https://doi.org/10.1016/j.ymeth.2015.10.012.
32. K. Chang, Y. Fang, P. He, et al., "Employing the Anchor DSPE-PEG as a Redox Probe for Ratiometric Electrochemical Detection of Surface Proteins on Extracellular Vesicles with Aptamers," *Analytical Chemistry* 95 (2023): 16194–16200, https://doi.org/10.1021/acs.analchem.3c02948.
33. R. Huang, L. He, Y. Xia, et al., "A Sensitive Aptasensor Based on a Hemin/G-Quadruplex-Assisted Signal Amplification Strategy for Electrochemical Detection of Gastric Cancer Exosomes," *Small* 15 (2019): e1900735, https://doi.org/10.1002/smll.201900735.
34. Y. Guo, S. Liu, H. Yang, et al., "Regenerable electrochemical biosensor for exosomes detection based on the dual-recognition proximity binding-induced DNA walker," *Sensors and Actuators: B. Chemical* 349 (2021): 130765, https://doi.org/10.1016/j.snb.2021.130765.
35. Y. Deng, T. Zhou, K. Hu, et al., "An electrochemical biosensor designed with entropy-driven autocatalytic DNA circuits for sensitive detection of ovarian cancer-derived exosomes," *Biosensors & Bioelectronics* 250 (2024): 116060, https://doi.org/10.1016/j.bios.2024.116060.
36. M. Wang, J. Shu, Y. Wang, et al., "Ultrasensitive PD-L1-Expressing Exosome Immunosensors Based on a Chemiluminescent Nickel-Cobalt Hydroxide Nanoflower for Diagnosis and Classification of Lung Adenocarcinoma," *ACS Sensors* 9 (2024): 3444–3454, https://doi.org/10.1021/acssensors.4c00954.
37. H. Cui, T. Zheng, N. Qian, et al., "Aptamer-Functionalized Magnetic Ti3C2 Based Nanoplatform for Simultaneous Enrichment and Detection of Exosomes," *Small* 20 (2024): e2402434, https://doi.org/10.1002/smll.202402434.
38. D. Zhang, L. Qiao, S. Xu, et al., "Accurate identification of exosomes based on proximity-induced autonomous

assembly of DNAzyme wires," *Sensors and Actuators: B. Chemical* 383 (2023): 133581, https://doi.org/10.1016/j.snb.2023.133581.
39. Y. Yu, W. Zhang, Y. Guo, et al., "Engineering of exosome-triggered enzyme-powered DNA motors for highly sensitive fluorescence detection of tumor-derived exosomes," *Biosensors & Bioelectronics* 167 (2020): 112482, https://doi.org/10.1016/j.bios.2020.112482.
40. Y. Wang, H. Jie, H. Ye, et al., "Methylene Blue-Stained Single-Stranded DNA Aptamers as a Highly Efficient Electronic Switch for Quasi-Reagentless Exosomes Detection: An Old Dog with New Tricks," *Analytical Chemistry* 95 (2023): 18166–18173, https://doi.org/10.1021/acs.analchem.3c03715.
41. X. Sun, B. Chen, Z. Li, et al., "Accurate diagnosis of thyroid cancer using a combination of surface-enhanced Raman spectroscopy of exosome on MXene-coated gold@silver core@shell nanoparticle substrate and deep learning," *Chemical Engineering Journal* 488 (2024): 150835, https://doi.org/10.1016/j.cej.2024.150835.
42. X. Lin, P. Zhou, Q. Li, et al., ""Three-in-One" Plasmonic Au@PtOs Nanocluster Driven Lateral Flow Assay for Multimodal Cancer Exosome Biosensing," *Analytical Chemistry* 96 (2024): 10686–10695, https://doi.org/10.1021/acs.analchem.4c01580.
43. L. Li, X. Chen, Y. Pei, et al., "A label-free fluorescence aptasensor for salivary exosomes based on a nano-micro dual-scale signal amplification strategy," *Talanta* 287 (2025): 127638, https://doi.org/10.1016/j.talanta.2025.127638.
44. C. Zhai, J. Long, J. He, et al., "Precise Identification and Profiling of Surface Proteins of Ultra Rare Tumor Specific Extracellular Vesicle with Dynamic Quantitative Plasmonic Imaging," *ACS Nano* 17 (2023): 16656–16667, https://doi.org/10.1021/acsnano.3c02853.
45. M. Wang, Y. Lin, S. Wu, et al., "An electrochemical biosensor for PD-L1 positive exosomes based on ultra-thin two-dimensional covalent organic framework nanosheets coupled with CRISPR-Cas12a mediated signal amplification," *Sensors and Actuators: B. Chemical* 362 (2022): 131813, https://doi.org/10.1016/j.snb.2022.131813.
46. P. Wei, F. Wu, B. Kang, et al., "Plasma extracellular vesicles detected by Single Molecule array technology as a liquid biopsy for colorectal cancer," *Journal of Extracellular Vesicles* 9 (2020): 1809765, https://doi.org/10.1080/20013078.2020.1809765.
47. Y. Yoshioka, N. Kosaka, Y. Konishi, et al., "Ultra-sensitive liquid biopsy of circulating extracellular vesicles using ExoScreen," *Nature Communications* 5 (2014): 3591, https://doi.org/10.1038/ncomms4591.
48. H. Im, H. Shao, Y. Park, et al., "Label-free detection and molecular profiling of exosomes with a nano-plasmonic sensor," *Nature Biotechnology* 32 (2014): 490–495, https://doi.org/10.1038/nbt.2886.
49. G. Li, Q. Cai, Y. Dong, et al., "Revisiting the Nanoflow Cytometric Quantification of Extracellular Vesicles Under the Framework of ICH Q14 Guidelines," *Journal of Extracellular Biology* 4 (2025): e70050, https://doi.org/10.1002/jex2.70050.
50. L. Zhao, H. Wang, J. Fu, et al., "Microfluidic-based exosome isolation and highly sensitive aptamer exosome membrane protein detection for lung cancer diagnosis," *Biosensors & Bioelectronics* 214 (2022): 114487, https://doi.org/10.1016/j.bios.2022.114487.
51. V. Moccia, C. Centelleghe, A. Zendrini, et al., "Isolation and preliminary characterization of extracellular vesicles from bottlenose dolphin (Tursiops truncatus) and long-finned pilot whale (Globicephala melas) blow," *PLoS One* 21 (2026): e0352853, https://doi.org/10.1371/journal.pone.0352853.
52. H. Xing, J. Wang, Y. Wang, et al., "Diagnostic Value of CA 19-9 and Carcinoembryonic Antigen for Pancreatic Cancer: A Meta-Analysis," *Gastroenterology Research and Practice* 2018 (2018): 8704751, https://doi.org/10.1155/2018/8704751.
53. J. Huang, G. Gao, Y. Ge, et al., "Development of a Serum-Based MicroRNA Signature for Early Detection of Pancreatic Cancer: A Multicenter Cohort Study," *Digestive Diseases and Sciences* 69 (2024): 1263–1273, https://doi.org/10.1007/s10620-024-08338-4.
54. Z. Zheng, Z. Lu, F. Yan, et al., "The role of novel biomarkers in the early diagnosis of pancreatic cancer: A systematic review and meta-analysis," *PLoS One* 20 (2025): e0322720, https://doi.org/10.1371/journal.pone.0322720.
